# One-pot synthesis of epoxides from benzyl alcohols and aldehydes

**DOI:** 10.3762/bjoc.14.205

**Published:** 2018-09-03

**Authors:** Edwin Alfonzo, Jesse W L Mendoza, Aaron B Beeler

**Affiliations:** 1 Department of Chemistry, Boston University, Boston, Massachusetts 02215, United States

**Keywords:** Corey–Chaykovsky, epoxide, heterocycle, one-pot, ylide

## Abstract

A one-pot synthesis of epoxides from commercially available benzyl alcohols and aldehydes is described. The reaction proceeds through in situ generation of sulfonium salts from benzyl alcohols and their subsequent deprotonation for use in Corey–Chaykovsky epoxidation of aldehydes. The generality of the method is exemplified by the synthesis of 34 epoxides that were made from an array of electronically and sterically varied alcohols and aldehydes.

## Introduction

Epoxides have historically served as strategic functional groups in target-oriented synthesis [1–4]. Common examples of their utility include stereospecific ring opening [5–7], rearrangements into carbonyls [8–17], and application to cascade or domino reactions [18–19]. More recently, our group has used benzyl epoxides for the photoredox generation of carbonyl ylides which are leveraged in the synthesis of cyclic ethers [20]. This work has led us to search for a general and operationally simple method to generate benzyl epoxides. One of the most powerful methods to access epoxides is through the Corey–Chaykovsky reaction [21] which uses sulfonium ylides and their subsequent reaction with carbonyl groups. This reaction has seen major advancement since its original disclosure, particular in the area of asymmetric synthesis [22–24]. Other notable advancements include the expansion of its scope by using organic bases and a one-pot oxidation/epoxidation sequence of benzyl alcohols with manganese dioxide and an exogenous sulfonium salt [25–26]. Despite these efforts, the synthesis of epoxides using this powerful transformation still often requires multiple steps due to the need to independently synthesize the sulfonium salt. Typically, the salt is synthesized from nucleophilic displacement of benzyl halides but the work by Aggarwal and co-workers [27] has demonstrated that these can be generated from inexpensive benzyl alcohols in the presence of tetrafluoroboric acid and a thio-trapping agent. Unfortunately, isolation of the salt was still required for use in epoxidation of carbonyl groups.

Inspired by these aforementioned precedents, we hypothesized that a process, using commercially available starting materials, wherein the sulfonium salt could be generated in situ from benzyl alcohols and deprotonated would provide an efficient protocol for the synthesis of epoxides in a single reaction. Herein, we describe the realization of this methodology and its use in the synthesis of epoxides that are often unattainable by standard epoxidation methods.

## Results and Discussion

**Scheme 1:**
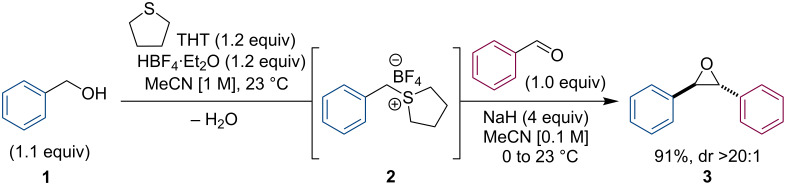
One-pot synthesis of epoxides from benzyl alcohols and aldehydes.

After evaluating numerous approaches toward the proposed reaction and subsequent optimization, we found that the sulfonium salt **2** (Scheme 1) could be generated in situ from benzyl alcohol (**1**) in the presence of slight excess of tetrafluoroboric acid in diethyl ether (HBF_4_·Et_2_O) and tetrahydrothiophene (THT). Notably, the use of acetonitrile (MeCN) as a solvent was critical for maintaining a homogeneous reaction and a successful outcome. We also observed that sodium hydride (NaH) was the only base that successfully afforded the desired epoxide **3**, typically in excellent and reproducible yields (other bases screened included KO*t*-Bu, LiHMDS, TBD [25], and KOH). Furthermore, diluting the reaction after formation of the sulfonium salt, and cooling it in an ice bath, proved essential to control the exotherm caused by the deprotonation.

**Figure 1:**
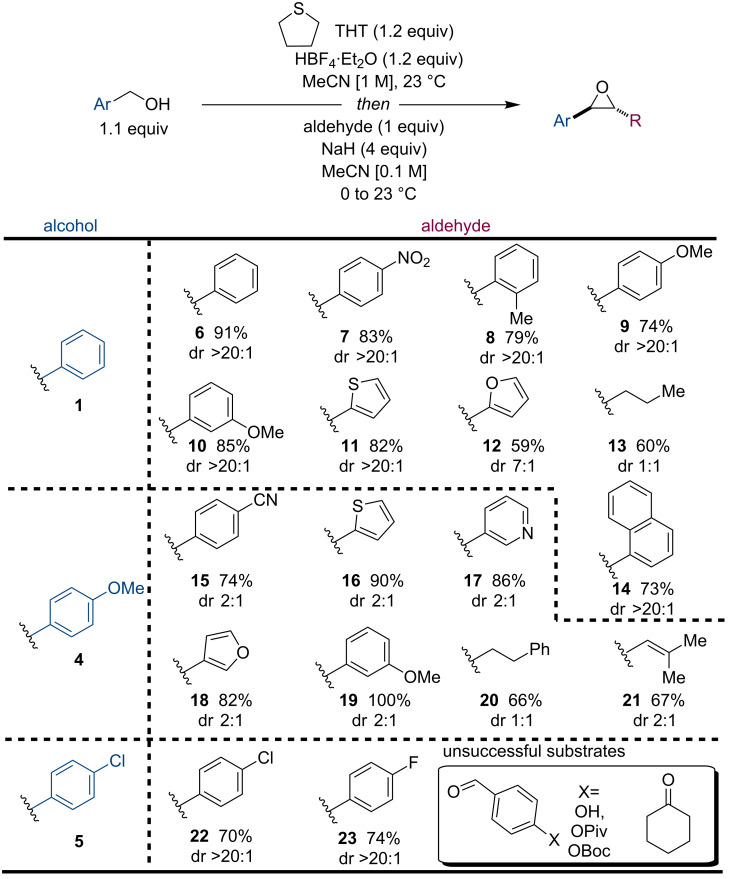
Scope of the one-pot synthesis of epoxides from benzyl alcohols and aldehydes.

Having established a robust method, we then turned our attention to evaluating the scope of the reaction using three electronically varied benzyl alcohols **1**, **4**, and **5** (Figure 1) on a preparative scale (3 mmol, 5 mmol, 10 mmol). A range of aryl aldehydes worked well including *para-*nitro (**7**), *ortho-*methyl (**8**), and *para*-methoxy groups (**9**). Other notable examples include heterocycles, such as basic pyridines, thiophenes, and furans **16**–**18**. Additionally, aliphatic **13** and **20** and alkenyl aldehydes **21** performed well providing synthetically useful quantities of the desired epoxides. Finally, epoxides containing electron-deficient aryl groups are also available with this method, as exemplified by the synthesis of compounds **22** and **23**, which were obtained in good yields on a 10 mmol scale. With respect to the reactivity of the benzyl alcohols, electron-rich alcohol **4** showed faster reaction rates and yields, presumably due to faster and more efficient formation of the sulfonium salt through a *para*-quinonemethide (*p-*QM) intermediate. Furthermore, the lower diastereomeric ratios (dr) observed for benzyl alcohol **4** may be due to competing *p-*QM formation at the betaine intermediate prior to epoxide formation through displacement of THT. This is supported by example **9** which contained a *para*-methoxy group at the aldehyde component but was isolated as a single diastereomer [28]. Functional groups that are not compatible with this method include phenols, esters, and ketones (Figure 1). The latter is most likely due to competing enolization of the ketone leading to undesired reactivity [29].

**Scheme 2:**
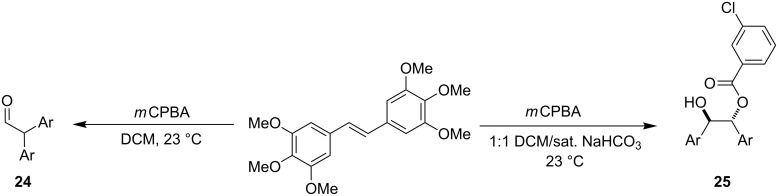
*m*CPBA epoxidation of electron-rich stilbene derivatives.

Many of the epoxides that are of interest to us are highly oxygenated on the aryl rings and can be used for the synthesis of numerous bioactive molecules [27,30–34]. Attempts at synthesizing one of these epoxides with the standard *m*CPBA epoxidation (Scheme 2) led exclusively to rearranged aldehyde **24**, presumably promoted by the carboxylic acid byproduct of *m*CPBA. Unfortunately, attempts to remedy this by using buffered conditions only led to an *m*CPBA-epoxide adduct **25** [35].

**Figure 2:**
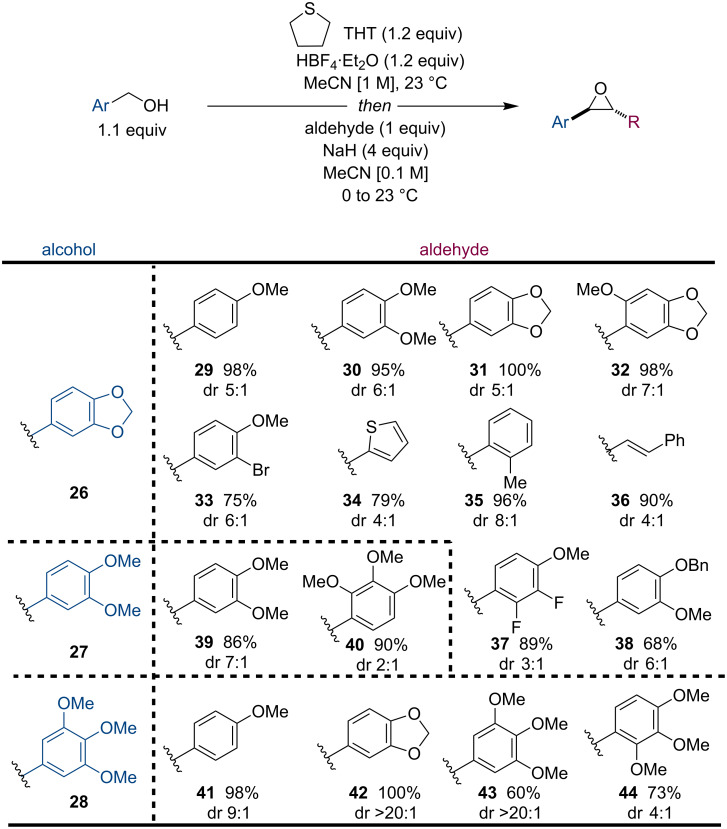
Scope of the reaction with electron-rich alcohols and aldehydes.

However, the one-pot reaction was highly successful with three electron-rich benzyl alcohols **26**, **27**, and **28** all bearing multiple oxygenation and with a large panel of electron-rich aldehydes (Figure 2). The reaction was highly successful even when both partners were poly-oxygenated (**29**–**32**). Other notable functionalities include heteroaromatics **34**, alkenes **36**, halides **33** and **37**, and a benzyl protected alcohol **38**. Furthermore, electron-donating groups were tolerated in all positions on the aryl groups (**41**–**44**). Although the Corey–Chaykovsky reaction has been well studied, nearly all the examples shown in Figure 2 represent new compounds and an extension to this methodology.

## Conclusion

In conclusion, we have developed a general and simple method to access benzylic epoxides through the Corey–Chaykovsky reaction between benzyl alcohols and aldehydes. This method provides expedient access to epoxides from commercially available materials in a step and time economical fashion. In particular, we have demonstrated its applicability to the synthesis of epoxides that were generally unattainable using the standard *m*CPBA epoxidation.

## Supporting Information

File 1 Experimental procedures and characterization for all new compounds described herein.
