# Supplementary material for: One-pot synthesis of epoxides from benzyl alcohols and aldehydes
Source: Beilstein J Org Chem. 2018 Sep 3;14:2308–12. doi: 10.3762/bjoc.14.205 (PMC6142752; doi:10.3762/bjoc.14.205)
Supplement: File 1 — Experimental procedures and characterization for all new compounds described herein. [file Beilstein_J_Org_Chem-14-2308-s001.pdf]

# Supporting Information

**for**

# One-pot synthesis of epoxides from benzyl alcohols and aldehydes

Edwin Alfonzo[1], Jesse W. L. Mendoza[1], and Aaron B. Beeler[1]*

Address: [1]Department of Chemistry, Boston University, Boston, Massachusetts 02215, United States

Email: Aaron B. Beeler - beelera@bu.edu

* Corresponding author

## Experimental procedures and characterization for all new compounds described herein

**Table of Contents**

**I. General information ……………………………………………………………………………………S1**
**II. Synthetic procedure for Corey–Chaykovsky epoxidation ………………...…....……..............….........S2**
**III. $^{1}$H, $^{13}$C NMR spectra of new compounds ……………..………………………..……….……..……..S20**

**General information:**

$^{1}$H NMR and $^{13}$C NMR spectra were recorded at ambient temperature on a Varian Agilent-500 MHz VNMRS (500 and 126 MHz, respectively), and are internally referenced to the residual protio solvent signal ($CDCl_3$: δ 7.26 and 77.0 ppm). Data for $^{1}$H NMR are reported as follows: chemical shift, multiplicity (brs = broad singlet, s = singlet, d = doublet, t = triplet, q = quartet, m = multiplet, *overlap* = overlapping peaks), coupling constants in Hz and integration. Data for $^{13}$C NMR are reported in terms of chemical shift and no special nomenclature is used for equivalent carbons. High-resolution mass spectra were obtained in the Boston University Chemical Instrumentation Center using a Waters Q-TOF APIUS mass spectrometer. Commercial reagents were purified prior to use following the guidelines of Chai and Armarego.[1] All solvents were purified according to the method of Grubbs.[2] Organic solutions were concentrated under reduced pressure on a Büchi rotary evaporator using a water bath. Chromatographic purification of products was accomplished by flash chromatography on Silicycle F60 silica gel or Sorbtech neutral alumina 32–63 µm according to the method of Still.[3] All reactions were carried out in well ventilated fume hoods. Reactions were monitored by thin-layer chromatography (TLC) using Silicycle 250 µm silica gel plates or Sorbtech neutral alumina 250 µm. Visualization of the developed chromatogram was performed by irradiation with a 254 nm ultra-violet (UV) light or treatment with aqueous potassium permanganate ($KMnO_4$) or ethanolic phosphomolybdic acid (PMA) followed by heating. Yields refer to purified compounds unless otherwise noted.

1. Chai, C.; Armarego, W. L. F. Purification of Laboratory Chemicals; 6th ed.; Butterworth Heinemann: Oxford, **2009**.
2. Pangborn, A. B.; Giardello, M. A.; Grubbs, R. H.; Rosen, R. K.; Timmers, F. J. *Organometallics* **1996**, *15*, 1518.
3. Still, W. C.; Kahn, M.; Mitra, A. *J. Org. Chem.* **1978**, *43*, 2923.

**General procedure for Corey–Chaykovsy epoxidation:**

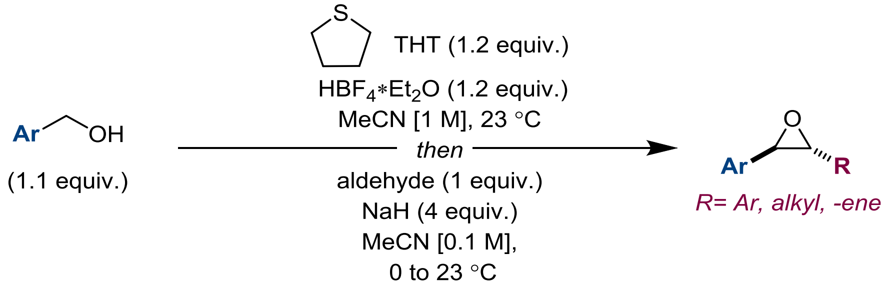


A flame-dried round bottom flask (for 3 mmol: 50 mL flask, 5 mmol: 100 mL, 10 mmol: 250 mL) with a magnetic stirring bar was charged with the benzyl alcohol (1.1 equiv), tetrahydrothiophene (THT) (1.2 equiv), and acetonitrile (MeCN) ([1 M] *with respect to (w.r.t.)* the benzyl alcohol). Thereafter, tetrafluoroboric acid diethyl ether complex ($HBF_4·OEt_2$) (1.2 equiv) was added dropwise and the reaction was left to stir until full consumption of the alcohol was observed by TLC (1:1 hexanes (Hex)/ethyl acetate (EtOAc)) or for a 12 h period. The reaction was then cooled to 0 °C (water/ice bath), diluted with MeCN ([0.1 M] *w.r.t.* aldehyde) and sodium hydride (NaH) (4 equiv) was added in small portions. After stirring for 5 minutes the aldehyde (1 equiv.) was added dropwise or in small portions (solids). The reaction was left to stir until full consumption of the aldehyde was seen by TLC (4:1 Hex/EtOAc) or for a 12 h period. Then, the reaction was cooled to 0 $^{\circ}$C and water was added dropwise until no further gas evolution was observed. The mixture was concentrated *in vacuo* and the resultant residue was introduced into a separatory funnel with the assistance of water and EtOAc. The mixture was extracted three times with EtOAc and the combined organic fractions were dried over $MgSO_4$ and concentrated in vacuo. The resultant material was purified by flash chromatography. Diastereoselectivity was determined by crude $^1$H NMR prior to purification unless otherwise noted.

Synthesis of ***(2R,3R)-2,3-diphenyloxirane (6)*** from *phenylmethanol* and *benzaldehyde*:

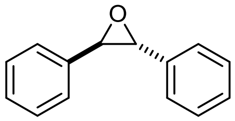


*6*
Chemical Formula: $C_{14}H_{12}O$
Exact Mass: 196.09

**Scale:** 3 mmol
**Flash Chromatography:** 10:1 Hex/EtOAc
**TLC:** $R_f$= 0.3 in 10:1 Hex/EtOAc, Stain: PMA (color:green)
**Physical state:** *white solid*
**% yield:** 91% (535 mg)
***d.r.*** >20:1
*trans:*
**$^1$H NMR (500 MHz, Chloroform-*d*)** δ 7.43 – 7.29 (m, 10H), 3.88 (s, 2H).
**$^{13}$C NMR (126 MHz, Chloroform-*d*)** δ 137.24, 128.70, 128.46, 125.64, 62.99.

Synthesis of ***(2R,3R)-2-(4-nitrophenyl)-3-phenyloxirane (7)*** from *phenylmethanol* and *4-nitrobenzaldehyde*:

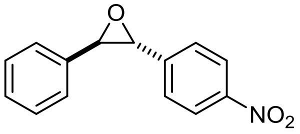


*7*
Chemical Formula: $C_{16}H_{13}NO_2$
Exact Mass: 251.09

**Scale:** 3 mmol
**Flash Chromatography:** 20:1 Hex/EtOAc → 10:1 Hex/EtOAc
**TLC:** $R_f$= 0.6 in 20:1 Hex/EtOAc, Stain: PMA (color: green)
**Physical state:** *yellow solid*
**% yield:** 83% (600 mg)
***d.r.*** >20:1
*trans:*
**$^1$H NMR (500 MHz, Chloroform-*d*)** δ 8.25 (d, *J* = 8.8 Hz, 2H), 7.52 (d, *J* = 8.8 Hz, 2H), 7.46 – 7.31 (m, 5H), 3.98 (d, *J* = 1.8 Hz, 1H), 3.86 (d, *J* = 1.8 Hz, 1H).
**$^{13}$C NMR (126 MHz, Chloroform-*d*)** δ 147.99, 144.54, 136.18, 128.96, 128.86, 126.38, 125.67, 124.03, 63.50, 61.81.

Synthesis of ***(2R,3R)-2-phenyl-3-(o-tolyl)oxirane (8)*** from *phenylmethanol* and *2-methylbenzaldehyde*:

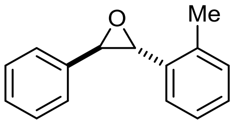


*8*
Chemical Formula: $C_{15}H_{14}O$
Exact Mass: 210.10

**Scale:** 3 mmol
**Flash Chromatography:** 20:1 Hex/EtOAc → 10:1 Hex/EtOAc
**TLC:** $R_f$= 0.5 in 20:1 Hex/EtOAc, Stain: PMA (color: green)
**Physical state:** *white solid*
**% yield:** 79% (501 mg)
***d.r.*** >20:1
*trans:*
**$^1$H NMR (500 MHz, Chloroform-*d*)** δ 7.42 – 7.29 (m, 6H), 7.29 – 7.21 (m, 2H), 7.21 – 7.15 (m, 1H), 4.00 (s, 1H), 3.77 (s, 1H), 2.36 (s, 3H).
**$^{13}$C NMR (126 MHz, Chloroform-*d*)** δ 137.42, 136.03, 135.67, 129.96, 128.73, 128.44, 127.90, 126.34, 125.61, 124.12, 62.06, 61.04, 19.06.

Synthesis of ***(2R,3R)-2-(4-methoxyphenyl)-3-phenyloxirane (9)*** from *phenylmethanol* and *4-methoxybenzaldehyde*:

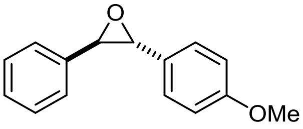


*9*
Chemical Formula: $C_{15}H_{14}O_2$
Exact Mass: 226.10

**Scale:** 3 mmol
**Flash Chromatography:** 10:1 Hex/EtOAc + 3% triethyl amine (TEA) → 10:1 Hex/EtOAc
**TLC:** $R_f$= 0.6 in 4:1 Hex/EtOAc, Stain: PMA (color: green)
**Physical state:** *white solid*
**% yield:** 74% (502 mg)
***d.r.*** >20:1
*trans:*
**$^1$H NMR (500 MHz, Chloroform-*d*)** δ 7.41 – 7.30 (m, 5H), 7.27 (d, *J* = 8.7 Hz, 2H), 6.92 (d, *J* = 8.7 Hz, 2H), 3.86 (d, *J* = 2.0 Hz, 1H), 3.83 (s, 3H), 3.82 (d, *J* = 2.0 Hz, 1H).
**$^{13}$C NMR (126 MHz, Chloroform-*d*)** δ 159.89, 137.38, 129.24, 128.67, 128.36, 126.94, 125.59, 114.16, 62.89, 62.80, 55.48.

Synthesis of ***(2R,3R)-2-(3-methoxyphenyl)-3-phenyloxirane (10)*** from *phenylmethanol* and *3-methoxybenzaldehyde*:

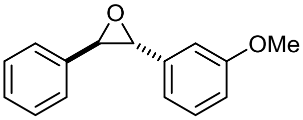


*10*
Chemical Formula: $C_{15}H_{14}O_2$
Exact Mass: 226.10

**Scale:** 3 mmol
**Flash Chromatography:** 20:1 Hex/$Et_2O$ → 10:1 Hex/$Et_2O$
**TLC:** $R_f$= 0.2 in 20:1 Hex/$Et_2O$, Stain: PMA (color: green)
**Physical state:** *clear oil*
**% yield:** 85% (577 mg)
***d.r.*** >20:1
*trans:*
**$^1$H NMR (500 MHz, Chloroform-*d*)** δ 7.46 – 7.32 (m, 5H), 7.32 – 7.24 (m, 1H), 6.99 – 6.93 (m, 1H), 6.93 – 6.86 (m, 2H), 3.86 (s, 2H), 3.83 (s, 3H).
**$^{13}$C NMR (126 MHz, Chloroform-*d*)** δ 160.08, 138.91, 137.16, 129.76, 128.69, 128.46, 125.62, 118.10, 114.24, 110.54, 62.88, 55.40, 55.39.

Synthesis of ***(2R,3S)-2-phenyl-3-(thiophen-2-yl)oxirane (11)*** from *phenylmethanol* and *2-thiophenecarboxaldehyde***:**

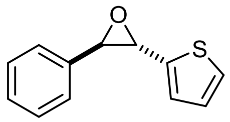

*11*
Chemical Formula: $C_{12}H_{10}OS$
Exact Mass: 202.05

**Scale:** 3 mmol
**Flash Chromatography:** 10:1 Hex/EtOAc + 3 % TEA → 10:1 Hex/EtOAc
**TLC:** $R_f$= 0.5 in 10:1 Hex/EtOAc, Stain: PMA (color: green)
**Physical state:** *red solid*
**% yield:** 82% (497 mg)
***d.r.*** >20:1
*trans:*
**$^1$H NMR (500 MHz, Chloroform-*d*)** δ 7.32-7.40 (m, 5H), 7.31 (d, *J* = 4.9 Hz, 1H), 7.15 (d, *J* =3.6, 1H), 7.00 (dd, *J* = 4.9, 3.6 Hz, 1H), 4.10 (d, *J* =1.7 Hz, 1H), 4.05 (d, *J* =1.7 Hz, 1H).
**$^{13}$C NMR (126 MHz, Chloroform-*d*)** δ 141.20, 136.66, 128.73, 128.61, 127.28, 126.11, 125.62, 125.34, 63.62, 59.68.

Synthesis of ***2-((2S,3R)-3-phenyloxiran-2-yl)furan (12)*** from *phenylmethanol* and *2-furancarboxaldehyde* as a mixture of separable diastereomer**:**

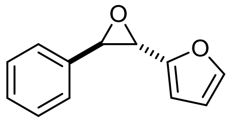

*12*
Chemical Formula: $C_{12}H_{10}O_2$
Exact Mass: 186.07

**Scale:** 3 mmol
**Flash Chromatography:** 20:1 Hex/EtOAc + 3 % TEA → 10:1 Hex/EtOAc
**TLC:** $R_f$= 0.3 in 10:1 Hex/EtOAc, Stain: PMA (color: green)
**Physical state:** *yellow oil*
**% yield:** 59% (330 mg)
***d.r.*** 7:1
*trans:*
**$^1$H NMR (500 MHz, Chloroform-*d*)** 7.25-7.60 (m, 6H), 6.49 (d, *J* =3.4 Hz, 1H), 6.39 (dd, *J* = 3.4, 2.0 Hz, 1H), 4.35 (d, *J* = 2.0 Hz, 1H) δ 3.91 (d, *J* =2.0 Hz, 1H).
**$^{13}$C NMR (126 MHz, Chloroform-*d*)** δ 149.96, 143.09, 136.58, 128.72, 128.61, 125.72, 110.93, 110.00, 59.72, 56.42.

Synthesis of ***(2R,3R)-2-phenyl-3-propyloxirane (13)*** from *phenylmethanol* and *propionaldehyde* as a mixture of inseparable diastereomer**:**

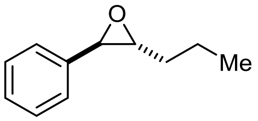

*13*
Chemical Formula: $C_{11}H_{14}O$
Exact Mass: 162.10

**Scale:** 3 mmol
**Flash Chromatography:** 20:1 Hex/$Et_2O$+ 3 % TEA → 20:1 Hex/$Et_2O$
**TLC:** $R_f$= 0.2 in 20:1 Hex/$Et_2O$, Stain: PMA (color: green)
**Physical state:** *yellow oil*
**% yield:** 60% (335 mg)
***d.r.*** 1:1
*trans:*
**$^1$H NMR (500 MHz, Chloroform-*d*)** δ 7.36-7.27 (m, 5H), 3.61 (d, 1H, *J* = 2.0 Hz), 2.95 (dt, 1H, *J* = 2.0, 5.5 Hz), 1.64-1.70 (m, 2H), 1.52-1.58 (m, 2H), 1.00 (t, 3H, *J* = 7.5 Hz).
**$^{13}$C NMR (126 MHz, Chloroform-*d*)** δ 138.05, 128.55, 128.09, 125.62, 63.17, 58.73, 34.49, 19.38, 14.11.
*cis:*
**$^1$H NMR (500 MHz, Chloroform-*d*)** δ 7.42 – 7.20 (m, 5H), 4.08 (d, *J* = 4.2 Hz, 1H), 3.22 (td, *J* = 6.0, 4.2 Hz, 1H), 1.49 – 1.32 (m, 2H), 1.28 – 1.20 (m, 2H), 0.85 (t, *J* = 7.2 Hz, 3H).
**$^{13}$C NMR (126 MHz, Chloroform-*d*)** δ 135.91, 128.55, 127.53, 126.60, 59.52, 57.51, 28.81, 19.50, 14.01.

Synthesis of ***(2R,3R)-2-(naphthalen-1-yl)-3-phenyloxirane (14)*** from *phenylmethanol* and *1-naphthaldehyde***:**

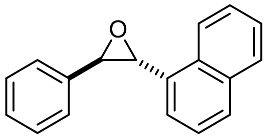

*14*
Chemical Formula: $C_{18}H_{14}O$
Exact Mass: 246.10

**Scale:** 3 mmol
**Flash Chromatography:** 20:1 Hex/EtOAc
**TLC:** $R_f$= 0.6 in 20:1 Hex/EtOAc, Stain: PMA (color: green)
**Physical state:** *yellow oil*
**% yield:** 73% (539 mg)
***d.r.*** >20:1
*trans:*
**$^1$H NMR (500 MHz, Chloroform-*d*)** δ 8.03 – 7.95 (m, 1H), 7.93 – 7.88 (m, 1H), 7.84 (d, *J* = 8.2 Hz, 1H), 7.66 – 7.58 (m, 1H), 7.55 – 7.48 (m, 3H), 7.48 – 7.37 (m, 5H), 4.51 (d, *J* = 1.9 Hz, 1H), 3.88 (d, *J* = 1.9 Hz, 1H).
**$^{13}$C NMR (126 MHz, Chloroform-*d*)** δ 137.31, 133.41, 133.37, 131.32, 128.85, 128.83, 128.59, 128.34, 126.55, 126.09, 125.75, 125.73, 123.02, 122.15, 62.15, 61.28.

Synthesis of ***4-((2R,3R)-3-(4-methoxyphenyl)oxiran-2-yl)benzonitrile (15)*** from *(4-methoxyphenyl)methanol* and *4-formylbenzonitrile* as a mixture of separable diastereomer**:**

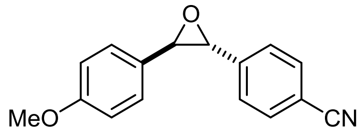

*15*
Chemical Formula: $C_{16}H_{13}NO_2$
Exact Mass: 251.09

**Scale:** 3 mmol
**Flash Chromatography:** 20:1 Hex/EtOAc + 3 % TEA → 10:1 Hex/EtOAc
**TLC:** $R_f^{trans, cis}$= 0.2, 0.1 in 10:1 Hex/EtOAc, Stain: PMA (color: green)
**Physical state:** *trans: white solid, cis: clear oil*
**% Yield:** 74% (557 mg)
***d.r.*** 2:1
*trans:*
**$^1$H NMR (500 MHz, Chloroform-*d*)** δ 7.66 (d, *J* = 8.4 Hz, 2H), 7.44 (d, *J* = 8.4 Hz, 2H), 7.26 (d, *J* = 8.7 Hz, 2H), 6.92 (d, *J* = 8.7 Hz, 2H), 3.90 (d, *J* = 1.7 Hz, 1H), 3.83 (s, 3H), 3.77 (d, *J* = 1.7 Hz, 1H).
**$^{13}$C NMR (126 MHz, Chloroform-*d*)** δ 160.19, 142.76, 132.51, 128.25, 126.98, 126.21, 118.78, 114.27, 112.01, 63.34, 61.84, 55.49.
*cis:*
**$^1$H NMR (500 MHz, Chloroform-*d*)** δ 7.47 (d, *J* = 8.2 Hz, 2H), 7.28 (d, *J* = 8.4 Hz, 2H), 7.04 (d, *J* = 8.7 Hz, 2H), 6.72 (d, *J* = 8.7 Hz, 2H), 4.38 (d, *J* = 4.2 Hz, 1H), 4.33 (d, *J* = 4.2 Hz, 1H), 3.72 (s, 3H).
**$^{13}$C NMR (126 MHz, Chloroform-*d*)** δ 159.32, 140.29, 131.75, 128.02, 127.65, 125.42, 118.79, 113.61, 111.44, 59.89, 59.12, 55.25.
**HRMS-ESI (m/z):** calculated $C_{16}H_{13}NO_2 + H^+$: 252.1025 found 252.1021

Synthesis of ***(2R,3S)-2-(4-methoxyphenyl)-3-(thiophen-2-yl)oxirane (16)*** from *(4-methoxyphenyl)methanol* and *thiophene-2-carbaldehyde* as a mixture of inseparable diastereomer**:**

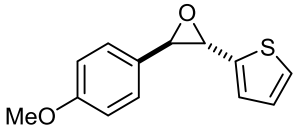

*16*
Chemical Formula: $C_{13}H_{12}O_2S$
Exact Mass: 232.06

**Scale:** 3 mmol
**Flash Chromatography:** 20:1 Hex/EtOAc+ 3 % TEA → 20:1 Hex/EtOAc
**TLC:** $R_f$= 0.4 in 10:1 Hex/EtOAc, Stain: PMA (color: green)
**Physical state:** *green oil*
**% Yield:** 90% (625 mg)
***d.r.*** 2:1
*trans:*
**$^1$H NMR (500 MHz, Chloroform-*d*)** δ 7.29 – 7.25 (*overlap*, 3H), 7.15 (dd, *J* = 3.5, 0.5 Hz, 1H), 7.01 (dd, *J* = 5.0, 3.5 Hz, 1H), 6.92 (d, *J* = 5.0, 2H), 4.10 (d, *J* = 1.9 Hz, 1H), 4.02 (d, *J* = 1.9 Hz, 1H), 3.82 (s, 3H).
**$^{13}$C NMR (126 MHz, Chloroform-*d*)** δ 160.00, 141.38, 128.37, 127.26, 126.96, 126.39, 125.96, 114.18, 63.53, 59.49, 55.47.
*cis:*
**$^1$H NMR (500 MHz, Chloroform-*d*)** δ 7.24 (d, *J* = 8.5 Hz, 2H), 7.11 (dd, *J* = 5.0, 1.3 Hz, 1H), 6.95 – 6.91 (*overlap*, 1H), 6.86 (dd, *J* = 5.0, 3.5 Hz, 1H), 6.83 (d, *J* = 8.5 Hz, 2H), 4.44 (d, *J* = 4.0 Hz, 1H), 4.37 (d, *J* = 4.0 Hz, 1H), 3.78 (s, 3H).

**$^{13}$C NMR (126 MHz, Chloroform-*d*)** δ 159.49, 137.68, 128.64, 127.44, 126.35, 125.86, 125.23, 113.54, 60.18, 56.74, 55.31.
**HRMS-ESI (m/z):** calculated $C_{13}H_{12}O_2S$+ $H^+$: 233.0636 found 233.0634.

Synthesis of ***3-((2R,3R)-3-(4-methoxyphenyl)oxiran-2-yl)pyridine (17)*** from *(4-methoxyphenyl)methanol* and *nicotinaldehyde* as a mixture of inseparable diastereomer**:**

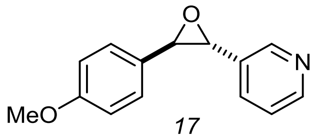


*17*
Chemical Formula: $C_{14}H_{13}NO_2$
Exact Mass: 227.09

**Scale:** 3 mmol
**Flash Chromatography:** 4:1 Hex/EtOAc+ 3 % TEA → 1:1 Hex/EtOAc
**TLC:** $R_f$= 0.2 in 4:1 Hex/EtOAc, Stain: PMA (color: green)
**Physical state:** *brown oil*
**% Yield:** 86% (587 mg)
***d.r.*** 2:1
*trans:*
**$^1$H NMR (500 MHz, Chloroform-*d*)** δ 8.62 (d, *J* = 2.0 Hz, 1H), 8.57 (dd, *J* = 4.8, 1.6 Hz, 1H), 7.61 (dt, *J* = 7.8, 2.0 Hz, 1H), 7.30 (dd, *J* = 7.8, 4.8 Hz, 1H), 7.27 (d, *J* = 8.7 Hz, 2H), 6.92 (d, *J* = 8.7 Hz, 2H), 3.89 (d, *J* = 1.8 Hz, 1H), 3.83 (d, *J* = 1.8 Hz, 1H), 3.82 (s, 3H).
**$^{13}$C NMR (126 MHz, Chloroform-*d*)** δ 160.10, 149.72, 147.90, 134.54, 132.77, 128.09, 126.96, 123.59, 114.24, 62.84, 60.60, 55.48.
*cis:*
**$^1$H NMR (500 MHz, Chloroform-*d*)** δ 8.47 (d, *J* = 2.1 Hz, 1H), 8.40 (dd, *J* = 4.8, 1.6 Hz, 1H), 7.40 (dt, *J* = 7.9, 2.1 Hz, 1H), 7.11 – 7.08 (*overlap*, 1H), 7.07 (d, *J* = 8.8 Hz, 2H), 6.72 (d, *J* = 8.7 Hz, 2H), 4.37 (d, *J* = 4.1 Hz, 1H), 4.31 (d, *J* = 4.1 Hz, 1H), 3.71 (s, 3H).
**$^{13}$C NMR (126 MHz, Chloroform-*d*)** δ 159.28, 148.99, 148.75, 133.00, 130.52, 128.47, 125.63, 122.80, 113.64, 59.52, 57.72, 55.25.
**LRMS-ESI (m/z):** calculated $C_{14}H_{13}NO_2$+ $H^+$: 228.1025 found 228.0972

Synthesis of ***3-((2R,3R)-3-(4-methoxyphenyl)oxiran-2-yl)furan (18)*** from *(4-methoxyphenyl)methanol* and *furan-3-carbaldehyde* as a mixture of inseparable diastereomer**:**

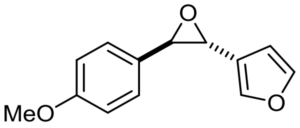


*18*
Chemical Formula: $C_{13}H_{12}O_3$
Exact Mass: 216.08

**Scale:** 3 mmol
**Flash Chromatography:** 20:1 Hex/EtOAc+ 3 % TEA → 10:1 Hex/EtOAc
**TLC:** $R_f$= 0.2 in 20:1 Hex/EtOAc, Stain: PMA (color: green)
**Physical state:** *clear oil*
**% Yield:** 82% (529 mg)
***d.r.*** 2:1
*trans:*
**$^1$H NMR (500 MHz, Chloroform-*d*)** δ 7.55 (s, 1H), 7.42 (t, *J* = 1.7 Hz, 1H), 7.26 (d, *J* = 8.7 Hz, 2H), 6.91 (d, *J* = 8.7 Hz, 2H), 6.39 (d, *J* = 1.7 Hz, 1H), 3.92 (d, *J* = 2.0 Hz, 1H), 3.82 (s, 3H), 3.80 (d, *J* = 2.0 Hz, 1H).
**$^{13}$C NMR (126 MHz, Chloroform-*d*)** δ 159.90, 143.72, 141.25, 128.05, 126.93, 123.03, 114.16, 108.12, 61.16, 56.38, 55.46.
*cis:*

**$^1$H NMR (500 MHz, Chloroform-*d*)** δ 7.23 – 7.18 (*overlap*, 3H), 7.17 (d, *J* = 0.8 Hz, 1H), 6.83 (d, *J* = 8.7 Hz, 2H), 6.03 – 5.99 (m, 1H), 4.29 (d, *J* = 4.0 Hz, 1H), 4.11 (d, *J* = 4.0 Hz, 1H), 3.79 (s, 3H).
**$^{13}$C NMR (126 MHz, Chloroform-*d*)** δ 159.31, 142.75, 141.56, 128.96, 127.02, 119.82, 113.56, 109.89, 58.98, 55.32, 53.72.
**LRMS-ESI (m/z):** calculated $C_{13}H_{12}O_3$+ $H^+$: 217.0865 found 217.0994

Synthesis of ***(2R,3R)-2-(3-methoxyphenyl)-3-(4-methoxyphenyl)oxirane (19)*** from *(4-methoxyphenyl)methanol* and *3-methoxybenzaldehyde* as a mixture of inseparable diastereomer**:**

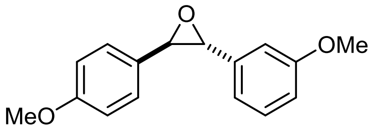


*19*
Chemical Formula: $C_{16}H_{16}O_3$
Exact Mass: 256.11

**Scale:** 5 mmol
**Flash Chromatography:** 20:1 Hex/EtOAc + 3 % TEA → 20:1 Hex/EtOAc → 10:1 Hex/EtOAc
**TLC:** $R_f$ = 0.4 in 10:1 Hex/EtOAc, Stain: PMA (color: green)
**% yield:** 100% (1.28 g)
**Physical state:** *clear oil*
***d.r.*** 2:1
*trans:*
**$^1$H NMR (500 MHz, Chloroform-*d*)** δ 7.33-7.25 (*overlap*, 3H), 6.99 – 6.84 (*overlap*, 5H), 3.85 (d, *J* = 1.9 Hz, 1H), 3.82 (s, 5H), 3.81 (d, *J* = 2.0 Hz, 1H).
**$^{13}$C NMR (126 MHz, Chloroform-*d*)** δ 159.94, 159.78, 138.94, 129.60, 128.10, 126.81, 117.94, 114.02, 113.30, 110.35, 62.68, 59.73, 55.27, 55.12.
*cis:*
**$^1$H NMR (500 MHz, Chloroform-*d*)** δ 7.15 – 7.07 (m, 3H), 6.82 – 6.69 (m, 5H), 4.30 (s, 2H), 3.73 (s, 3H), 3.69 (s, 3H).
**$^{13}$C NMR (126 MHz, Chloroform-*d*)** δ 159.11, 158.97, 136.16, 129.03, 128.87, 126.40, 119.37, 114.03, 113.47, 112.03, 62.59, 59.57, 55.34, 55.10.

Synthesis of ***(2R,3R)-2-(4-methoxyphenyl)-3-phenethyloxirane (20)*** from *(4-methoxyphenyl)methanol* and *3-phenylpropanal* as a mixture of inseparable diastereomer**:**

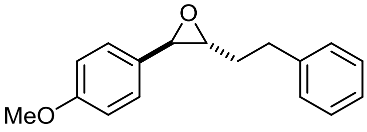


*20*
Chemical Formula: $C_{17}H_{18}O_2$
Exact Mass: 254.13

**Scale:** 5 mmol
**Flash Chromatography:** 20:1 Hex/$Et_2O$ + 3 % TEA → 20:1 Hex/$Et_2O$ → 10:1 Hex/$Et_2O$
**TLC:** $R_f$ = 0.1 in 20:1 Hex/$Et_2O$, Stain: PMA (color: green)
**% yield:** 66% (840 mg)
**Physical state:** *clear oil*
***d.r.*** 1:1
*trans:*
**$^1$H NMR (500 MHz, Chloroform-*d*)** δ 7.34 – 6.83 (*overlap*, 9H), 4.04 (d, *J* = 4.1 Hz, 1H), 3.82 (s, 3H), 3.22 (ddd, *J* = 6.6, 6.0, 4.1 Hz, 1H), 2.73 (m, 1H), 2.64 (m, 1H), 1.73 (m, 1H), 1.59 (m, 1H).
**$^{13}$C NMR (126 MHz, Chloroform-*d*)** δ 159.14, 141.29, 129.66, 128.60, 128.47, 127.65, 126.05, 113.63, 58.95, 57.46, 55.39, 32.37, 28.67.
*cis:*

**$^1$H NMR (500 MHz, Chloroform-*d*)** δ 7.34 – 6.83 (*overlap*, 9H), 3.81 (s, 3H), 3.51 (d, *J* = 2.1 Hz, 1H), 3.00 (ddd, *J* = 6.1, 5.5, 2.1 Hz, 1H), 2.90 (m, 1H), 2.79 (m, 1H), 2.09 – 1.93 (*overlap*, 2H).
**$^{13}$C NMR (126 MHz, Chloroform-*d*)** δ 159.64, 141.33, 129.53, 128.55, 128.43, 126.94, 126.16, 113.97, 62.31, 58.77, 55.41, 34.29, 32.26.
**LRMS-ESI (m/z):** calculated $C_{17}H_{18}O_2$+ $H^+$: 255.1385 found 255.1499

Synthesis of ***(2R,3R)-2-(4-methoxyphenyl)-3-(2-methylprop-1-en-1-yl)oxirane (21)*** from *(4-methoxyphenyl)methanol* and *3-methylbut-2-enal* as a mixture of inseparable diastereomer**:**

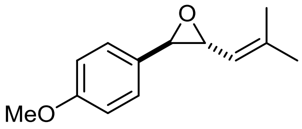


*21*
Chemical Formula: $C_{13}H_{16}O_2$
Exact Mass: 204.12

**Scale:** 5 mmol
**Flash Chromatography:** 20:1 Hex/$Et_2O$ + 3 % TEA → 20:1 Hex/$Et_2O$ → 10:1 Hex/$Et_2O$
**TLC:** $R_f$ = 0.1 in 20:1 Hex/$Et_2O$, Stain: PMA (color: green)
**% yield:** 67% (689 mg)
**Physical state:** *clear oil*
***d.r.*** 2:1
*trans:*
**$^1$H NMR (500 MHz, Chloroform-*d*)** δ 7.22 (d, *J* = 8.8 Hz, 2H), 6.89 (d, *J* = 8.8 Hz, 2H), 5.00 (dp, *J* = 8.7, 1.4 Hz, 1H), 3.81 (s, 3H), 3.72 (d, *J* = 2.1 Hz, 1H), 3.51 (dd, *J* = 8.7, 2.1 Hz, 1H), 1.80 (d, *J* = 1.4 Hz, 3H), 1.79 (d, *J* = 1.4 Hz, 3H).
**$^{13}$C NMR (126 MHz, Chloroform-*d*)** δ 159.67, 140.59, 129.67, 126.84, 122.09, 114.06, 60.04, 59.86, 55.43, 26.07, 18.54.
*cis:*
**$^1$H NMR (500 MHz, Chloroform-*d*)** δ 7.24 (d, *J* = 8.9 Hz, 2H), 6.89 (d, *J* = 8.9 Hz, 2H), δ 4.76 (dp, *J* = 8.7, 1.4 Hz, 1H), 4.18 (d, *J* = 4.2 Hz, 1H), 3.84 (dd, *J* = 8.7, 4.2 Hz, 1H), 3.81 (s, 3H), 1.81 (d, *J* = 1.4 Hz, 3H), 1.66 (d, *J* = 1.4 Hz, 3H).
**$^{13}$C NMR (126 MHz, Chloroform-*d*)** δ 159.18, 142.36, 127.82, 127.77, 117.84, 113.64, 58.94, 56.19, 55.36, 26.28, 18.62.
**LRMS-ESI (m/z):** calculated $C_{13}H_{16}O_2$+ $H^+$: 205.1229 found 205.1317

Synthesis of ***(2R,3R)-2,3-bis(4-chlorophenyl)oxirane (22)*** from *(4-chlorophenyl)methanol* and *4-chlorobenzaldehyde*:

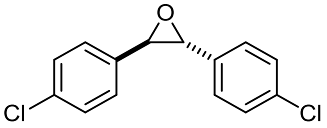


*22*
Chemical Formula: $C_{14}H_{10}Cl_2O$
Exact Mass: 264.01

**Scale:** 10 mmol
**Flash Chromatography:** 4:1 Hex/DCM
**TLC:** $R_f$ = 0.3 in 4:1 Hex/DCM, Stain: PMA (color:green)
**% yield:** 70% (1.85 g)
**Physical state:** *white solid*
***d.r.*** >20:1
**$^1$H NMR (500 MHz, Chloroform-*d*)** δ 7.36 (d, *J* = 8.7 Hz, 4H), 7.26 (d, *J* = 8.7 Hz, 4H), 3.80 (s,2H)
**$^{13}$C NMR (126 MHz, Chloroform-*d*)** δ 135.39, 134.40, 128.97, 126.95, 62.36.

Synthesis of ***(2R,3R)-2-(4-chlorophenyl)-3-(4-fluorophenyl)oxirane (23)*** from *(4-chlorophenyl)methanol* and *4-fluorobenzaldehyde*:

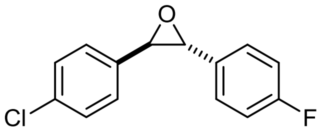

*23*
Chemical Formula: $C_{14}H_{10}ClFO$
Exact Mass: 248.04

**Scale:** 10 mmol
**Flash Chromatography:** 20:1 → 10:1 Hex/EtOAc
**TLC:** $R_f$ = 0.3 in 10:1 Hex/EtOac, Stain: PMA (color:green)
**% yield:** 74% (1.85 g)
**Physical state:** *white solid*
***d.r.*** >20:1
**$^1$H NMR (500 MHz, Chloroform-*d*)** δ 7.39 – 7.33 (m, 2H), 7.33 – 7.25 (m, 4H), 7.11 – 7.02 (m, 2H), 3.81 (s, 2H).
**$^{13}$C NMR (126 MHz, Chloroform-*d*)** δ 163.96, 161.99, 135.53, 134.34, 132.64, 132.61, 128.95, 127.35, 127.29, 126.95, 115.85, 115.68, 62.43, 62.29.

Synthesis of ***5-((2R,3R)-3-(4-methoxyphenyl)oxiran-2-yl)benzo[d][1,3]dioxole (29)*** from *benzo[d][1,3]dioxol-5-ylmethanol* and *4-methoxybenzaldehyde* as a mixture of inseparable diastereomer**:**

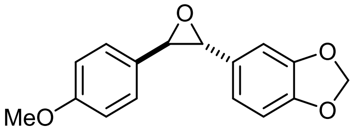

*29*
Chemical Formula: $C_{16}H_{14}O_4$
Exact Mass: 270.09

**Scale:** 5 mmol
**Flash Chromatography:** 9:1 Hex/EtOAc + 3 % TEA → 9:1 Hex/EtOAc
**TLC:** $R_f$= 0.3 in 9:1 Hex/EtOAc, Stain: PMA (color: green)
**% yield:** 98% (1.325 g)
**Physical state**: *clear oil*
***d.r.*** 5:1
*trans:*
**$^1$H NMR (500 MHz, Chloroform-*d*)** δ 7.25 (d, *J* = 8.7 Hz, 2H), 6.91 (d, *J* = 8.7 Hz, 2H), 6.84 (dd, *J* = 8.0, 1.6 Hz, 1H), 6.82 – 6.77 (*overlap*, 2H), 5.97 (s, 2H), 3.82 (s, 3H), 3.78 (d, *J* = 1.9 Hz, 1H), 3.77 (d, *J* = 1.9 Hz, 1H).
**$^{13}$C NMR (126 MHz, Chloroform-*d*)** δ 159.89, 148.20, 147.80, 131.38, 129.17, 126.90, 119.64, 114.16, 108.44, 105.58, 101.30, 62.78, 62.70, 55.49.
*cis:*
**$^1$H NMR (500 MHz, Chloroform-*d*)** δ 7.09 (d, *J* = 8.6 Hz, 2H), 6.75 (d, *J* = 8.8 Hz, 2H), 6.68 (dd, *J* = 8.0, 1.6 Hz, 1H), 6.66 – 6.61 (m, 2H), 5.88 (q, *J* = 1.5 Hz, 2H), 4.24 (q, *J* = 4.2 Hz, 2H), 3.74 (s, 3H).
**$^{13}$C NMR (126 MHz, Chloroform-*d*)** δ 159.11, 147.35, 147.03, 128.59, 128.23, 126.52, 120.61, 113.48, 107.96, 107.50, 101.03, 59.82, 59.71, 55.28.
**HRMS-ESI (m/z):** calculated $C_{16}H_{14}O_4 + H^+$: 271.0970 found 271.0988.

Synthesis of **5-((2R,3R)-3-(3,4-dimethoxyphenyl)oxiran-2-yl)benzo[*d*][1,3]dioxole (30)** from *benzo[d][1,3]dioxol-5-ylmethanol* and *3,4-dimethoxybenzaldehyde* as a mixture of separable diastereomer but only the major compound was characterized**:**

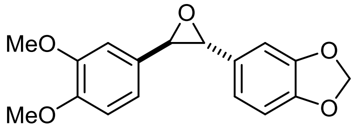

*30*
Chemical Formula: $C_{17}H_{16}O_5$
Exact Mass: 300.10

**Scale:** 10 mmol
**Flash Chromatography:** 4:1 Hex/DCM + 3 % TEA → 4:1 Hex/DCM + 1 % MeOH
**TLC:** $R_f$= 0.3 in 4:1 Hex/DCM + 1 % MeOH, Stain: PMA (color: green)
**% yield:** 95% (2.85 g)
**Physical state**: *white solid*
***d.r.*** 6:1
**$^1$H NMR (500 MHz, Chloroform-*d*)** δ 6.92 (dd, *J* = 8.2, 1.9 Hz, 1H), 6.84 – 6.77 (overlap, 5H), 5.97 (s, 2H), 3.90 (s, 3H), 3.89 (s, 3H), 3.77 (s, 2H).
**$^{13}$C NMR (126 MHz, Chloroform-*d*)** δ 149.29, 149.13, 148.06, 147.69, 131.10, 129.53, 119.51, 118.28, 111.07, 108.31, 107.81, 105.42, 101.16, 62.70, 62.68, 55.98, 55.89.
**HRMS-ESI (m/z):** calculated $C_{17}H_{16}O_5 + H^+$: 301.1076 found 301.1082.

Synthesis of ***(2R,3R)-2,3-bis(benzo[d][1,3]dioxol-5-yl)oxirane (31)*** from *benzo[d][1,3]dioxol-5-ylmethanol* and *benzo[d][1,3]dioxole-5-carbaldehyde* as a mixture of separable diastereomer but only the major compound was characterized:

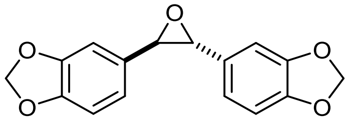

*31*
Chemical Formula: $C_{16}H_{12}O_5$
Exact Mass: 284.07

**Scale:** 6.45 mmol
**Flash Chromatography:** 4:1 Hex/EtOAc + 3 % TEA → 4:1 Hex/EtOAC
**TLC:** $R_f$= 0.3 in 4:1 Hex/EtOAC, Stain: PMA (color: green)
**% yield:** 100% (1.85 g)
**Physical state:** *white solid*
***d.r.*** 5:1
**$^1$H NMR (500 MHz, Chloroform-*d*)** δ 6.83 (dd, *J* = 8.0, 1.6 Hz, 2H), 6.80 (d, *J* = 8.0 Hz, 2H), 6.78 (d, *J* = 1.6 Hz, 2H), 5.97 (s, 5H), 3.74 (s, 2H).
**$^{13}$C NMR (126 MHz, Chloroform-*d*)** δ 148.20, 147.85, 131.14, 119.66, 108.44, 105.55, 101.31, 62.77.

Synthesis of ***5-((2R,3R)-3-(benzo[d][1,3]dioxol-5-yl)oxiran-2-yl)-6-methoxybenzo[d][1,3]dioxole (32)*** from *benzo[d][1,3]dioxol-5-ylmethanol* and *6-methoxybenzo[d][1,3]dioxole-5-carbaldehyde* as a mixture of inseparable diastereomer**:**

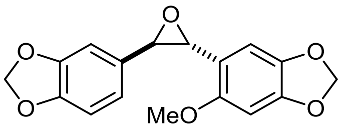

*32*
Chemical Formula: $C_{17}H_{14}O_6$
Exact Mass: 314.08

**Scale:** 3 mmol
**Flash Chromatography:** 4:1 Hex/EtOAc + 3 % TEA → 4:1 Hex/EtOAc
**TLC:** $R_f$= 0.4 in 4:1 Hex/EtOAc, Stain: PMA (color: green)
**% yield:** 98% (922 mg)
**Physical state:** *yellow solid*
***d.r.*** 7:1
**$^1$H NMR (500 MHz, Chloroform-*d*)** δ 6.85 – 6.78 (m, 2H), 6.77 (d, *J* = 1.5 Hz, 1H), 6.54 – 6.49 (m, 2H), 5.97 (*overlap*, 4H), 3.90 (s, 3H), 3.73 (d, *J* = 1.8 Hz, 1H), 3.71 (d, *J* = 1.8 Hz, 1H).
**$^{13}$C NMR (126 MHz, Chloroform-*d*)** δ 149.24, 148.20, 147.88, 143.97, 135.38, 131.86, 131.00, 119.67, 108.45, 105.53, 104.91, 101.76, 101.32, 99.67, 62.88, 62.83, 56.69.
*cis:*
**$^1$H NMR (500 MHz, Chloroform-*d*)** δ 6.73 – 6.64 (m, 2H), 6.63 (d, *J* = 1.6 Hz, 1H), 6.38 – 6.33 (m, 2H), 5.91 – 5.87 (*overlap*, 4H), 4.22 (d, *J* = 4.1 Hz, 1H), 4.20 (d, *J* = 4.1 Hz, 1H), 3.78 (s, 3H).
**$^{13}$C NMR (126 MHz, Chloroform-*d*)** δ 148.53, 147.40, 147.14, 143.31, 134.73, 128.98, 128.29, 120.56, 107.98, 107.41, 106.24, 101.56, 101.27, 101.08, 59.89, 59.82, 56.49.
**HRMS-ESI (m/z):** calculated $C_{16}H_{14}O_4 + Na^+$: 337.0688 found 337.0702

Synthesis of ***5-((2R,3R)-3-(3-bromo-4-methoxyphenyl)oxiran-2-yl)benzo[d][1,3]dioxole (33)*** from *benzo[d][1,3]dioxol-5-ylmethanol* and *3-bromo-4-methoxybenzaldehyde* as a mixture of separable diastereomer but only the major compound was characterized:

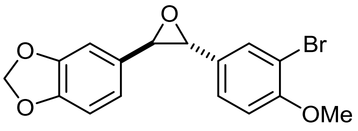

*33*
Chemical Formula: $C_{16}H_{13}BrO_4$
Exact Mass: 348.00

**Scale:** 3 mmol
**Flash Chromatography:** 10:1 Hex/EtOAc + 3 % TEA → 10:1 Hex/EtOAc
**TLC:** $R_f$= 0.3 in 10:1 Hex/EtOAc, Stain: PMA (color: green)
**% yield:** 75% (790 mg)
**Physical state:** *off-white solid*
***d.r.*** 6:1
**$^1$H NMR (500 MHz, Chloroform-*d*)** δ 7.50 (d, *J* = 2.1 Hz, 1H), 7.24 (dd, *J* = 8.4, 2.1 Hz, 1H), 6.89 (d, *J* = 8.4 Hz, 1H), 6.85 – 6.79 (overlap, 2H), 6.77 (d, *J* = 1.4 Hz, 1H), 5.98 (s, 2H), 3.91 (s, 3H), 3.77 – 3.72 (m, 2H).
**$^{13}$C NMR (126 MHz, Chloroform-*d*)** δ 155.91, 148.09, 147.79, 130.75, 130.62, 130.29, 125.84, 119.57, 111.94, 111.80, 108.33, 105.39, 101.19, 62.71, 61.65, 56.34.
**HRMS-ESI (m/z):** calculated $C_{16}H_{13}BrO_4 + H^+$: 350.0075 found 350.0195.

Synthesis of ***5-((2R,3S)-3-(thiophen-2-yl)oxiran-2-yl)benzo[d][1,3]dioxole (34)*** from *benzo[d][1,3]dioxol-5-ylmethanol* and *thiophene-2-carbaldehyde* as a mixture of separable diastereomer but only the major compound was characterized:

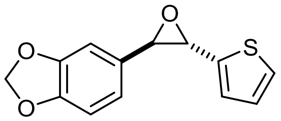


*34*
Chemical Formula: $C_{13}H_{10}O_3S$
Exact Mass: 246.04

**Scale:** 3 mmol
**Flash Chromatography:** 20:1 Hex/$Et_2O$ + 3 % TEA → 20:1 Hex/$Et_2O$
**TLC:** $R_f$= 0.2 in 20:1 Hex/$Et_2O$, Stain: PMA (color: green)
**% yield:** 79% (583 mg)
**Physical state:** *clear oil*
***d.r.*** 4:1
**$^1$H NMR (500 MHz, Chloroform-*d*)** δ 7.29 (dd, *J* = 5.0, 1.1 Hz, 1H), 7.14 (dd, *J* = 3.5, 1.1 Hz, 1H), 7.01 (dd, *J* = 5.0, 3.5 Hz, 1H), 6.85 (dd, *J* = 8.0, 1.6 Hz, 1H), 6.81 (d, *J* = 8.0 Hz, 1H), 6.78 (d, *J* = 1.6 Hz, 1H), 5.98 (s, 2H), 4.05 (d, *J* = 1.9 Hz, 1H), 3.98 (d, *J* = 1.9 Hz, 1H).
**$^{13}$C NMR (126 MHz, Chloroform-*d*)** δ 148.23, 147.99, 141.13, 130.60, 127.28, 126.06, 125.32, 119.77, 108.48, 105.56, 101.35, 63.61, 59.51.
**HRMS-ESI (m/z):** calculated $C_{13}H_{10}O_3S + H^+$: 247.0429 found 247.0420.

Synthesis of ***5-((2R,3R)-3-(o-tolyl)oxiran-2-yl)benzo[d][1,3]dioxole (35)*** from *benzo[d][1,3]dioxol-5-ylmethanol* 2-methylbenzaldehyde as a mixture of separable diastereomer but only the major compound was characterized:

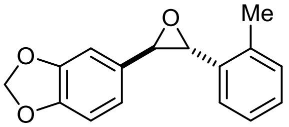


*35*
Chemical Formula: $C_{16}H_{14}O_3$
Exact Mass: 254.09

**Scale:** 3 mmol
**Flash Chromatography:** 20:1 Hex/$Et_2O$ + 3 % TEA → 20:1 Hex/$Et_2O$
**TLC:** $R_f$ = 0.2 in 20:1 Hex/$Et_2O$, Stain: PMA (color: green)
**% yield:** 96% (728 mg)
**Physical state:** *clear oil*
***d.r.*** 8:1
**$^1$H NMR (500 MHz, Chloroform-*d*)** δ 7.36 – 7.29 (m, 1H), 7.28 – 7.21 (m, 2H), 7.21 – 7.14 (m, 1H), 6.88 (dd, *J* = 7.9, 1.7 Hz, 1H), 6.85 – 6.80 (m, 2H), 5.99 (s, 2H), 3.95 (d, *J* = 2.0 Hz, 1H), 3.69 (d, *J* = 2.0 Hz, 1H), 2.36 (s, 3H).
**$^{13}$C NMR (126 MHz, Chloroform-*d*)** δ 148.22, 147.87, 135.96, 135.60, 131.39, 129.94, 127.87, 126.33, 124.05, 119.65, 108.50, 105.58, 101.32, 62.04, 60.89, 19.04.
**HRMS-ESI (m/z):** calculated $C_{16}H_{14}O_3 + H^+$: 255.1021 found 255.1022.

Synthesis of ***5-((2R,3R)-3-((E)-styryl)oxiran-2-yl)benzo[d][1,3]dioxole (36)*** from *benzo[d][1,3]dioxol-5-ylmethanol* and *cinnamaldehyde* as a mixture of inseparable diastereomer:

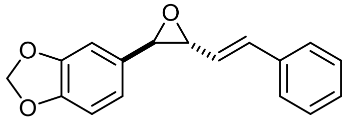

*36*
Chemical Formula: $C_{17}H_{14}O_3$
Exact Mass: 266.09

**Scale:** 3 mmol
**Flash Chromatography:** 20:1 Hex/$Et_2O$ + 3 % TEA → 20:1 Hex/$Et_2O$
**TLC:** $R_f$ = 0.3 in 20:1 Hex/$Et_2O$, Stain: PMA (color: green)
**% yield:** 90% (715 mg)
**Physical state:** *clear oil*
***d.r.*** 4:1
*trans:*
**$^1$H NMR (500 MHz, Chloroform-*d*)** δ 7.40 (m, 2H), 7.34 (m, 2H), 7.30 – 7.25 (*overlap*, 2H), 6.84 – 6.78 (m, 2H), 6.76 (d, *J* = 16.0 Hz, 1H), 6.05 (dd, *J* = 16.0, 7.7 Hz, 1H), 5.97 (s, 2H), 3.82 (d, *J* = 1.9 Hz, 1H), 3.48 (dd, *J* = 7.7, 1.9 Hz, 1H).
**$^{13}$C NMR (126 MHz, Chloroform-*d*)** δ 148.19, 147.81, 136.15, 134.52, 131.07, 128.80, 128.28, 126.62, 126.20, 119.66, 108.44, 105.57, 101.29, 63.08, 60.87.
*cis:*
**$^1$H NMR (500 MHz, Chloroform-*d*)** δ 7.40 (m, 2H), 7.34 (m, 2H), 7.30 – 7.25 (*overlap*, 2H), 6.84 – 6.76 (*overlap*, 3H), 5.97 (*overlap*, 2H), 5.76 (dd, *J* = 15.9, 8.7 Hz, 1H), 4.25 (d, *J* = 4.1 Hz, 1H), 3.79 (dd, *J* = 8.7, 4.1 Hz, 1H).
**$^{13}$C NMR (126 MHz, Chloroform-*d*)** δ 147.79, 147.35, 137.04, 136.26, 129.19, 128.67, 128.23, 126.65, 123.17, 120.00, 108.33, 107.02, 101.23, 60.27, 59.43.
**HRMS-ESI (m/z):** calculated $C_{17}H_{14}O_3 + H^+$: 267.1021 found 267.1022.

Synthesis of ***5-((2R,3R)-3-(2,3-difluoro-4-methoxyphenyl)oxiran-2-yl)benzo[d][1,3]dioxole (37)*** from *benzo[d][1,3]dioxol-5-ylmethanol* and *2,3-difluoro-4-methoxybenzaldehyde* as a mixture of inseparable diastereomer:

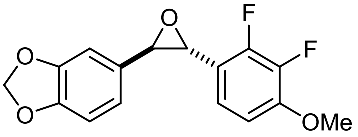

*37*
Chemical Formula: $C_{16}H_{12}F_2O_4$
Exact Mass: 306.07

**Scale:** 4 mmol
**Flash Chromatography:** 20:1 Hex/EtOAc + 3 % TEA → 10:1 Hex/EtOAc
**TLC:** $R_f$ = 0.2 in 10:1 Hex/EtOAc, Stain: PMA (color: green)
**% yield:** 89% (1.09 g)
**Physical state:** *off-white solid*
***d.r.*** 3:1
*trans:*
**$^1$H NMR (500 MHz, Chloroform-*d*)** δ 6.96 (m, 1H), 6.91 – 6.70 (m, 4H), 5.97 (s, 2H), 4.02 (d, *J* = 1.9 Hz, 1H), 3.91 (s, 3H), 3.78 (d, *J* = 1.9 Hz, 1H).
**$^{13}$C NMR (126 MHz, Chloroform-*d*)** δ 151.28 (d, *J* = 11.4 Hz), 149.30 (d, *J* = 11.4 Hz), 148.97 (dd, *J* = 8.0, 3.2 Hz), 148.26, 147.26, 142.01 (d, *J* = 13.9 Hz), 140.04 (d, *J* = 14.0 Hz), 130.47, 119.87, 119.52 (t, *J* = 4.5 Hz), 118.33 (d, *J* = 10.6 Hz), 108.49, 108.44 (d, *J* = 3.3 Hz), 105.63, 101.37, 62.20 (d, *J* = 1.3 Hz), 56.81, 56.69 (dd, *J* = 5.0, 3.1 Hz).
*cis:*

**$^1$H NMR (500 MHz, Chloroform-*d*)** δ 6.92 – 6.84 (m, 1H), 6.71 – 6.56 (m, 4H), 5.88 (s, 2H), 4.33 (d, *J* = 4.2 Hz, 1H), 4.30 (d, *J* = 4.2 Hz, 1H), 3.83 (s, 3H).
**$^{13}$C NMR (126 MHz, Chloroform-*d*)** δ 150.73 (d, *J* = 11.3 Hz), 148.76 (d, *J* = 11.3 Hz), 148.61 (dd, *J* = 7.9, 3.3 Hz), 147.41, 147.26, 141.66 (d, *J* = 13.6 Hz), 139.69 (d, *J* = 13.8 Hz), 127.90, 122.22 (t, *J* = 4.6 Hz), 120.29, 115.83 (d, *J* = 11.6 Hz), 108.02, 107.47 (d, *J* = 3.3 Hz), 107.04, 101.10, 59.19, 56.58, 55.39 (t, *J* = 3.6 Hz).
**HRMS-ESI (m/z):** calculated $C_{16}H_{12}F_2O_4$+ $H^+$: 307.0782 found 307.0775.

Synthesis of ***5-((2R,3R)-3-(4-(benzyloxy)-3-methoxyphenyl)oxiran-2-yl)benzo[d][1,3]dioxole (38)*** from *benzo[d][1,3]dioxol-5-ylmethanol* and *4-(benzyloxy)-3-methoxybenzaldehyde* as a mixture of separable diastereomer but only the major compound was characterized:

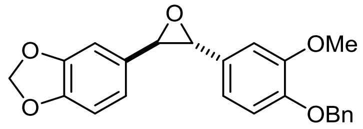


*38*
Chemical Formula: $C_{23}H_{20}O_5$
Exact Mass: 376.13

**Scale:** 5 mmol
**Flash Chromatography:** 20:1 Hex/EtOAc + 3 % TEA → 4:1 Hex/EtOAc
**TLC:** $R_f$ = 0.5 in 4:1 Hex/EtOAc, Stain: PMA (color: green)
**% yield:** 68% (1.32 g)
**Physical state:** *white solid*
***d.r.*** 6:1
**$^1$H NMR (500 MHz, Chloroform-*d*)** δ 7.48 – 7.41 (m, 2H), 7.40 – 7.33 (m, 2H), 7.33 – 7.28 (m, 1H), 6.90 – 6.77 (m, 6H), 5.97 (s, 2H), 5.17 (s, 2H), 3.91 (s, 3H), 3.76 (m, 2H).
**$^{13}$C NMR (126 MHz, Chloroform-*d*)** δ 150.10, 148.34, 148.18, 147.81, 137.11, 131.22, 130.22, 128.68, 127.98, 127.34, 119.64, 118.31, 114.03, 108.50, 108.43, 105.54, 101.29, 71.18, 62.80, 62.79, 56.13.
**HRMS-ESI (m/z):** calculated $C_{23}H_{20}O_5$ + $Na^+$: 399.1208 found 399.1198.

Synthesis of ***(2R,3R)-2,3-bis(3,4-dimethoxyphenyl)oxirane (39)*** from *(3,4-dimethoxyphenyl)methanol* and *3,4-dimethoxybenzaldehyde* as a mixture of separable diastereomer but only the major compound was characterized:

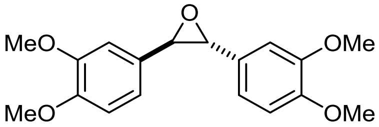


*39*
Chemical Formula: $C_{18}H_{20}O_5$
Exact Mass: 316.13

**Scale:** 6.45 mmol
**Flash Chromatography:** 4:1 Hex/DCM + 3 % TEA → 1:1 Hex/DCM + 1 % MeOH
**TLC:** $R_f$= 0.4 in 4:1 Hex/DCM, Stain: PMA (color: green)
**% yield:** 86% (1.757 g)
**Physical state:** *white solid*
***d.r.*** 7:1
**$^1$H NMR (500 MHz, Chloroform-*d*)** δ 6.93 (dd, *J* = 8.2, 1.9 Hz, 2H), 6.87 (d, *J* = 8.2 Hz, 2H), 6.84 (d, *J* = 2.0 Hz, 2H), 3.90 (s, 6H), 3.89 (s, 6H), 3.81 (s, 2H).
**$^{13}$C NMR (126 MHz, Chloroform-*d*)** δ 149.49, 149.31, 129.82, 118.44, 111.30, 108.06, 62.92, 56.17, 56.07.

Synthesis of ***(2R,3R)-2-(3,4-dimethoxyphenyl)-3-(2,3,4-trimethoxyphenyl)oxirane (40)*** from *(3,4-dimethoxyphenyl)methanol* and *2,3,4-trimethoxybenzaldehyde* as a mixture of inseparable diastereomer:

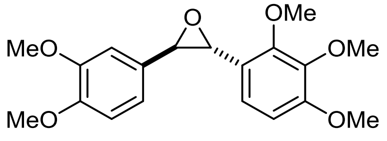


*40*
Chemical Formula: $C_{19}H_{22}O_6$
Exact Mass: 346.14

**Scale:** 5 mmol
**Flash Chromatography:** 4:1 Hex/EtOAc + 3 % TEA→ 4:1 Hex/EtOAc
**TLC:** $R_f$ = 0.1 in 4:1 Hex/EtOAc
**% yield:** 90% (1.55 g)
**Physical state:** *white solid*
***d.r.*** *2:1*
*trans:*
**$^{1}$H NMR (500 MHz, Chloroform-*d*)** δ 6.94 (d, *J* = 8.6 Hz, 1H), 6.89 – 6.83 (m, 3H), 6.70 (d, *J* = 8.6 Hz, 1H), 4.09 (d, *J* = 2.0 Hz, 1H), 3.90 (s, 3H), 3.89 (s, 3H), 3.88 (s, 3H), 3.87 (s, 3H), 3.87 (s, 3H), 3.77 (d, *J* = 2.0 Hz, 1H).
**$^{13}$C NMR (126 MHz, Chloroform-*d*)** δ 153.65, 152.66, 149.38, 149.20, 142.03, 129.89, 123.35, 122.70, 119.65, 111.19, 108.11, 107.64, 62.36, 61.61, 61.04, 58.41, 56.18, 56.09, 56.02.
*cis:*
**$^{1}$H NMR (500 MHz, Chloroform-*d*)** δ 6.93 (d, *J* = 8.4 Hz, 1H), 6.80 (d, *J* = 8.4 Hz, 1H), 6.70 (dd, *J* = 8.5, 2.0 Hz, 1H), 6.58 (d, *J* = 2.0 Hz, 1H), 6.53 (d, *J* = 8.5 Hz, 1H), 4.35 (d, *J* = 4.1 Hz, 1H), 4.28 (d, *J* = 4.1 Hz, 1H), 3.79 (s, 3H), 3.79 (s, 3H), 3.77 (s, 3H), 3.68 (s, 6H).
**$^{13}$C NMR (126 MHz, Chloroform-*d*)** δ 153.42, 152.14, 148.87, 148.30, 141.67, 127.43, 120.84, 119.65, 118.54, 110.53, 109.74, 106.46, 60.93, 60.85, 59.41, 57.69, 56.04, 55.87, 55.73.
**HRMS-ESI (m/z):** calculated $C_{19}H_{22}O_6 + H^+$: 347.1495 found 347.1491.

Synthesis of ***(2R,3R)-2-(4-methoxyphenyl)-3-(3,4,5-trimethoxyphenyl)oxirane (41)*** from *(3,4,5-trimethoxyphenyl)methanol* and *4-methoxybenzaldehyde* as a mixture of separable diastereomer but only the major compound was characterized:

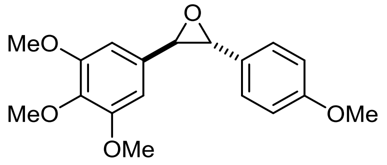


*41*
Chemical Formula: $C_{18}H_{20}O_5$
Exact Mass: 316.13

**Scale:**10 mmol
**Flash Chromatography:** 9:1 Hex/EtOAc + 3 % TEA → 4:1 Hex/EtOAc
**TLC:** $R_f$= 0.4 in 4:1 Hex/EtOAc, Stain: PMA (color: green)
**% yield:** 98% (3.24 g)
**Physical state**: *clear oil*
***d.r.*** 9:1
**$^{1}$H NMR (500 MHz, Chloroform-*d*)** δ 7.26 (d, *J* = 8.7 Hz, 2H), 6.92 (d, *J* = 8.7 Hz, 2H), 6.57 (s, 2H), 3.87 (s, 6H), 3.85 (s, 3H), 3.82 (*overlap*, 4H), 3.77 (d, *J* = 1.7 Hz, 1H).
**$^{13}$C NMR (126 MHz, Chloroform-*d*)** δ 159.94, 153.69, 137.95, 133.07, 129.03, 126.91, 114.18, 102.17, 62.95, 62.83, 61.02, 56.25, 55.49.
**HRMS-ESI (m/z):** calculated $C_{18}H_{20}O_5 + H^+$: 317.1389 found 317.1393.

Synthesis of ***5-((2R,3R)-3-(3,4,5-trimethoxyphenyl)oxiran-2-yl)benzo[d][1,3]dioxole (42)*** from *(3,4,5-trimethoxyphenyl)methanol* and *benzo[d][1,3]dioxole-5-carbaldehyde*:

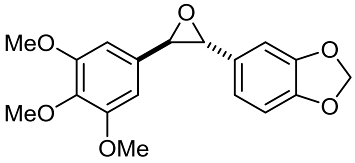


*42*
Chemical Formula: $C_{18}H_{18}O_6$
Exact Mass: 330.11

**Scale:** 10 mmol
**Flash Chromatography:** 9:1 + 3 % TEA → 4:1 Hex/EtOAc
**TLC:** $R_f$= 0.3 in 4:1 Hex/EtOAc, Stain: PMA (c:green)
**% yield:** 100% (3.3 g)
**Physical state:** *clear oil*
***d.r.*** >20:1
**$^{13}$C NMR (126 MHz, Chloroform-*d*)** δ 153.71, 148.24, 147.92, 138.02, 132.85, 131.01, 119.71, 108.48, 105.55, 102.19, 101.34, 62.95, 62.91, 61.04, 56.26.
**$^{1}$H NMR (500 MHz, Chloroform-*d*)** δ 6.87 – 6.77 (m, 3H), 6.56 (s, 2H), 5.97 (s, 2H), 3.87 (s, 6H), 3.85 (s, 3H), 3.78 (d, *J* = 1.9 Hz, 1H), 3.74 (d, *J* = 1.9 Hz, 1H).
**HRMS-ESI (m/z):** calculated $C_{18}H_{18}O_6 + H^+$: 331.1182 found 331.1180.

Synthesis of ***(2R,3R)-2,3-bis(3,4,5-trimethoxyphenyl)oxirane (43)*** from *(3,4,5-trimethoxyphenyl)methanol* and *3,4,5-trimethoxybenzaldehyde* as a mixture of separable diastereomer but only the major compound was characterized:

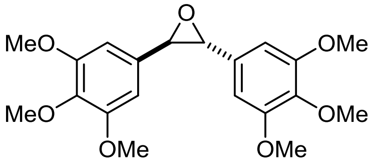


*43*
Chemical Formula: $C_{20}H_{24}O_7$
Exact Mass: 376.15

**Scale:** 5 mmol
**Flash Chromatography:** 4:1 Hex/DCM + 3% TEA→ 4:1 Hex/DCM + 1% MeOH → 3:1 Hex/DCM + 1% MeOH → 3:1 Hex/DCM + 3% MeOH
**TLC:** $R_f$ = 0.1 in 4:1 Hex/DCM + 3% MeOH
**% yield:** 60% (1.123 g)
**Physical state:** *white solid*
***d.r.*** >20:1 *(determined after purification)*
**$^{1}$H NMR (500 MHz, Chloroform-*d*)** δ 6.57 (s, 4H), 3.87 (s, 12H), 3.85 (s, 6H), 3.78 (s, 2H).
**$^{13}$C NMR (126 MHz, Chloroform-*d*)** δ 153.70, 138.05, 132.69, 102.18, 63.08, 61.00, 56.24.
**HRMS-ESI (m/z):** calculated $C_{20}H_{24}O_7 + Na^+$: 399.1420 found 399.1432

Synthesis of ***(2R,3R)-2-(2,3,4-trimethoxyphenyl)-3-(3,4,5-trimethoxyphenyl)oxirane (44)*** from *(3,4,5-trimethoxyphenyl)methanol* and *2,3,4-trimethoxybenzaldehyde* as a mixture of inseparable diastereomer:

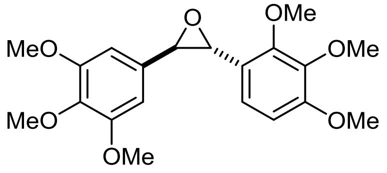


*44*
Chemical Formula: $C_{20}H_{24}O_7$
Exact Mass: 376.15

**Scale:** 5 mmol
**Flash Chromatography:** 10:1 Hex/$Et_2O$ + 3 % TEA→ 3:1 Hex/$Et_2O$
**TLC:** $R_f$ = 0.2 in 4:1 Hex/EtOAc
**% yield:** 73% (1.38 g)
**Physical state:** *clear oil*
***d.r.*** 4:1
*trans:*
**$^1$H NMR (500 MHz, Chloroform-*d*)** δ 6.93 (d, *J* = 8.7 Hz, 1H), 6.69 (d, *J* = 8.7 Hz, 1H), 6.58 (s, 2H), 4.05 (d, *J* = 1.9 Hz, 1H), 3.89 (s, 3H), 3.88 (s, 3H), 3.87 (*overlap*, 9H), 3.85 (s, 3H), 3.77 (d, *J* = 2.0 Hz, 1H).
**$^{13}$C NMR (126 MHz, Chloroform-*d*)** δ 153.73, 153.64, 152.68, 142.02, 137.94, 133.06, 123.07, 119.69, 107.63, 102.30, 62.44, 61.62, 61.02, 60.98, 58.53, 56.23, 56.18.
*cis:*
**$^1$H NMR (500 MHz, Chloroform-*d*)** δ 6.94 (d, *J* = 8.5 Hz, 1H), 6.53 (d, *J* = 8.5 Hz, 1H), 6.37 (s, 2H), 4.35 (d, *J* = 4.3 Hz, 1H), 4.26 (d, *J* = 4.3 Hz, 1H), 3.79 (s, 3H), 3.76 (s, 3H), 3.75 (s, 3H), 3.70 (*overlap*, 9H).
**$^{13}$C NMR (126 MHz, Chloroform-*d*)** δ 153.50, 152.76, 152.10, 141.64, 137.36, 130.44, 122.68, 120.62, 106.42, 103.94, 60.92, 60.84, 59.58, 57.69, 56.23, 56.06, 56.04.
**HRMS-ESI (m/z):** calculated $C_{20}H_{24}O_7$ + $Na^+$: 399.1420 found 399.1411.

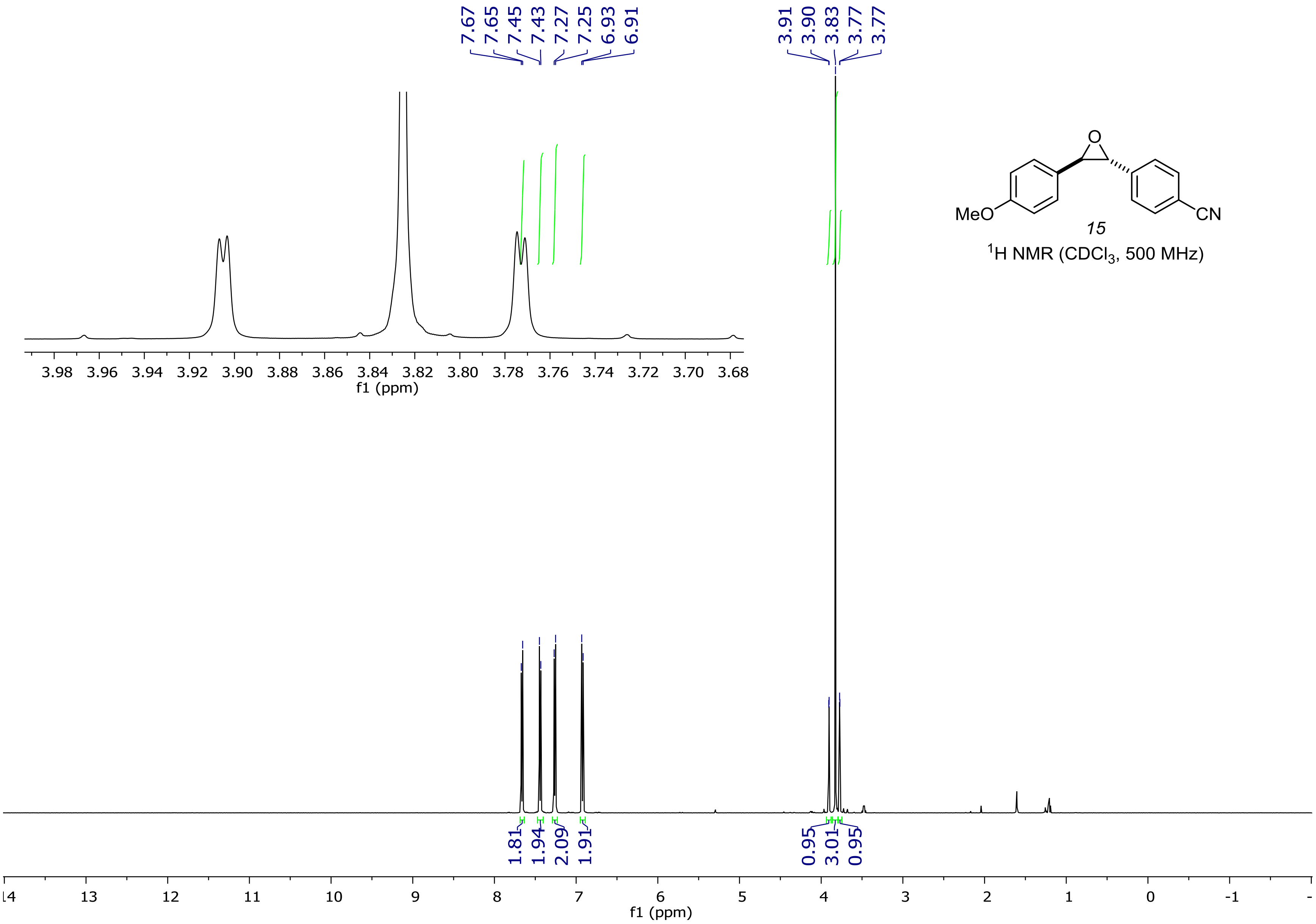
7.67
7.65
7.45
7.43
7.27
7.25
6.93
6.91
3.91
3.90
3.83
3.77
3.77
MeO
CN
O
15
1H NMR (CDCl3, 500 MHz)
1.81
1.94
2.09
1.91
0.95
3.01
0.95
f1 (ppm)

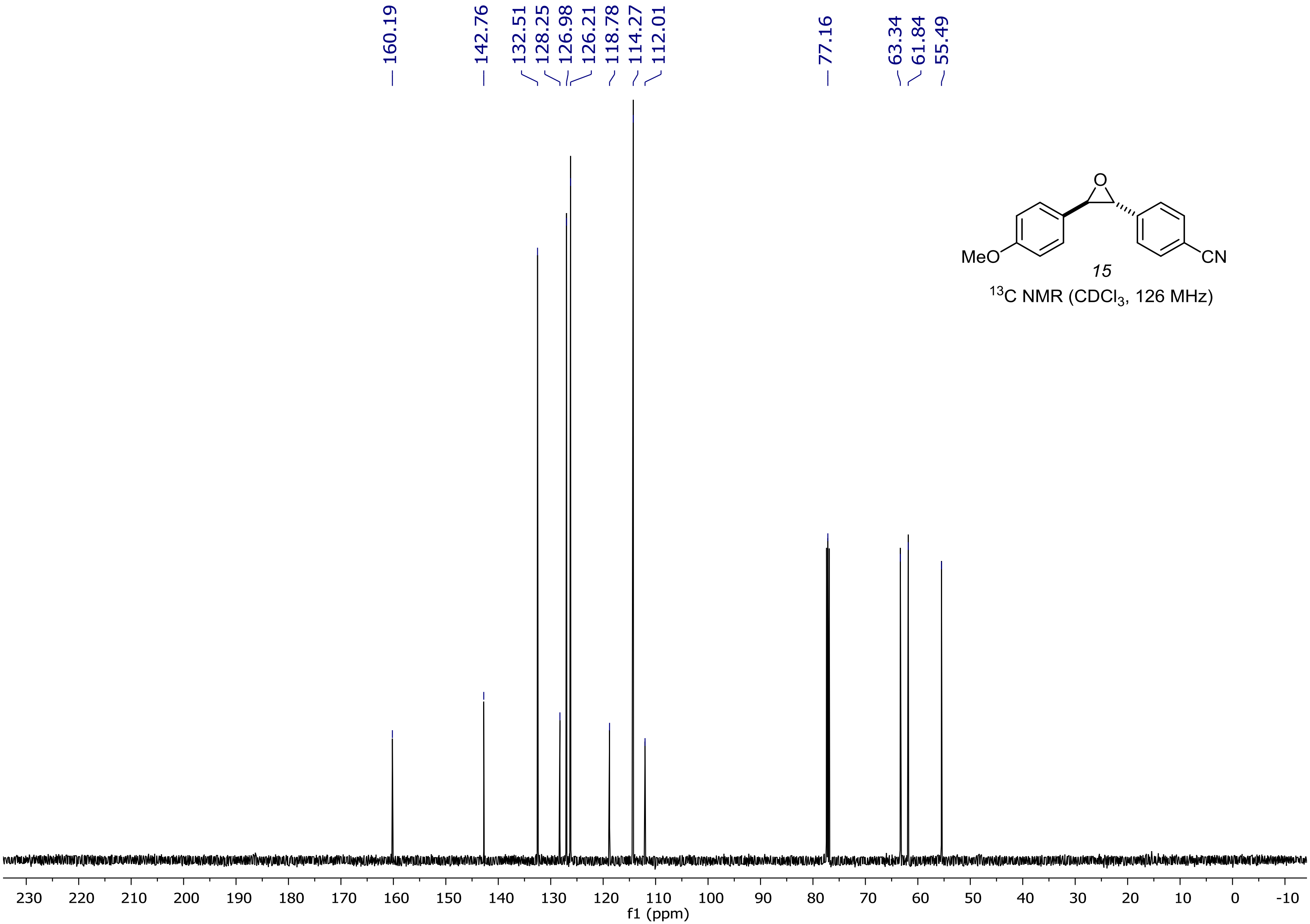
160.19
142.76
132.51
128.25
126.98
126.21
118.78
114.27
112.01
77.16
63.34
61.84
55.49
MeO
O
CN
15
13C NMR (CDCl3, 126 MHz)
230 220 210 200 190 180 170 160 150 140 130 120 110 100 90 80 70 60 50 40 30 20 10 0 -10
f1 (ppm)

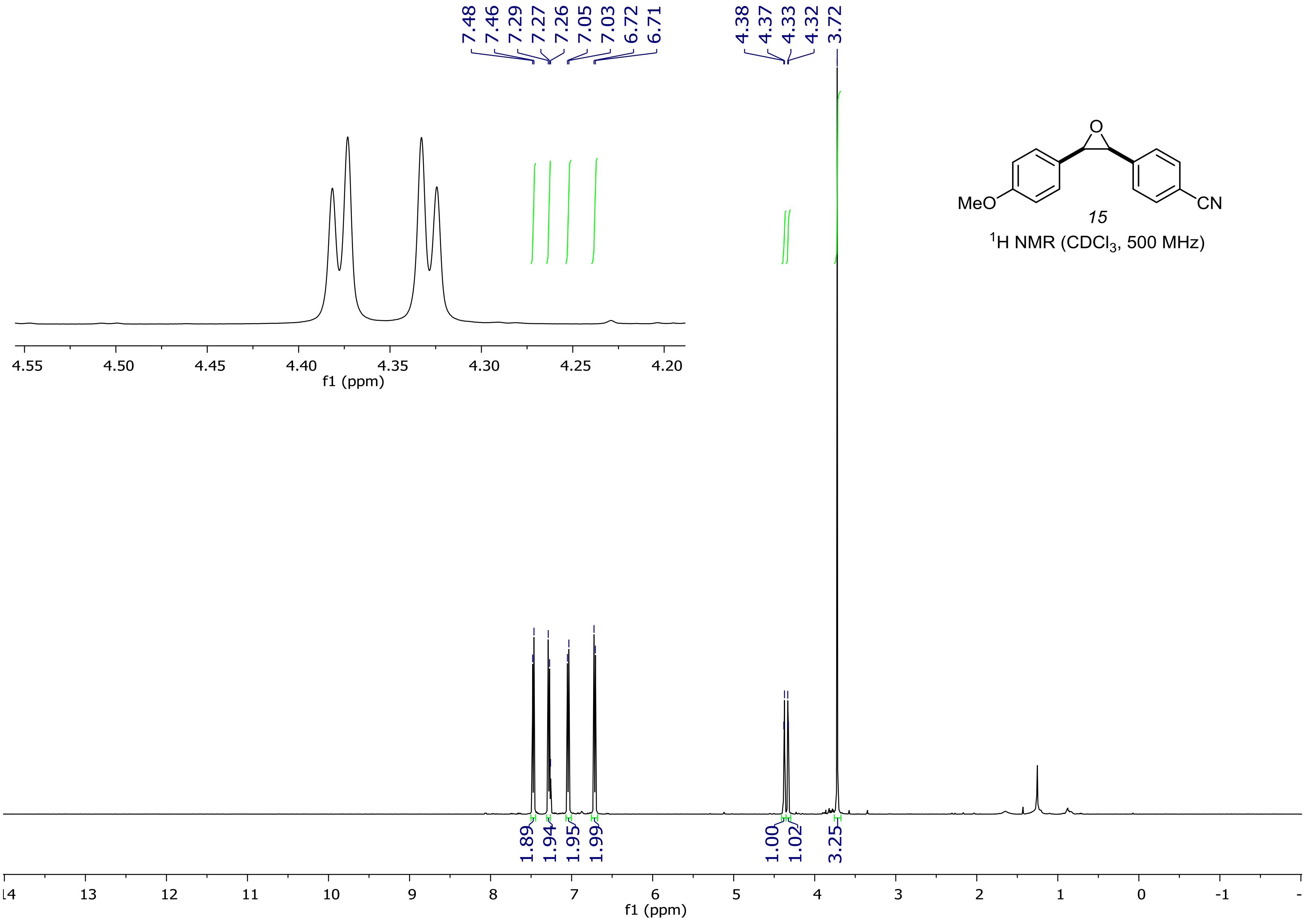
7.48
7.46
7.29
7.27
7.26
7.05
7.03
6.72
6.71
4.38
4.37
4.33
4.32
3.72
MeO
CN
O
15
$^{1}$H NMR ($CDCl_3$, 500 MHz)
4.55
4.50
4.45
4.40
4.35
4.30
4.25
4.20
f1 (ppm)
1.89
1.94
1.95
1.99
1.00
1.02
3.25
13
12
11
10
9
8
7
6
5
4
3
2
1
0
-1
f1 (ppm)

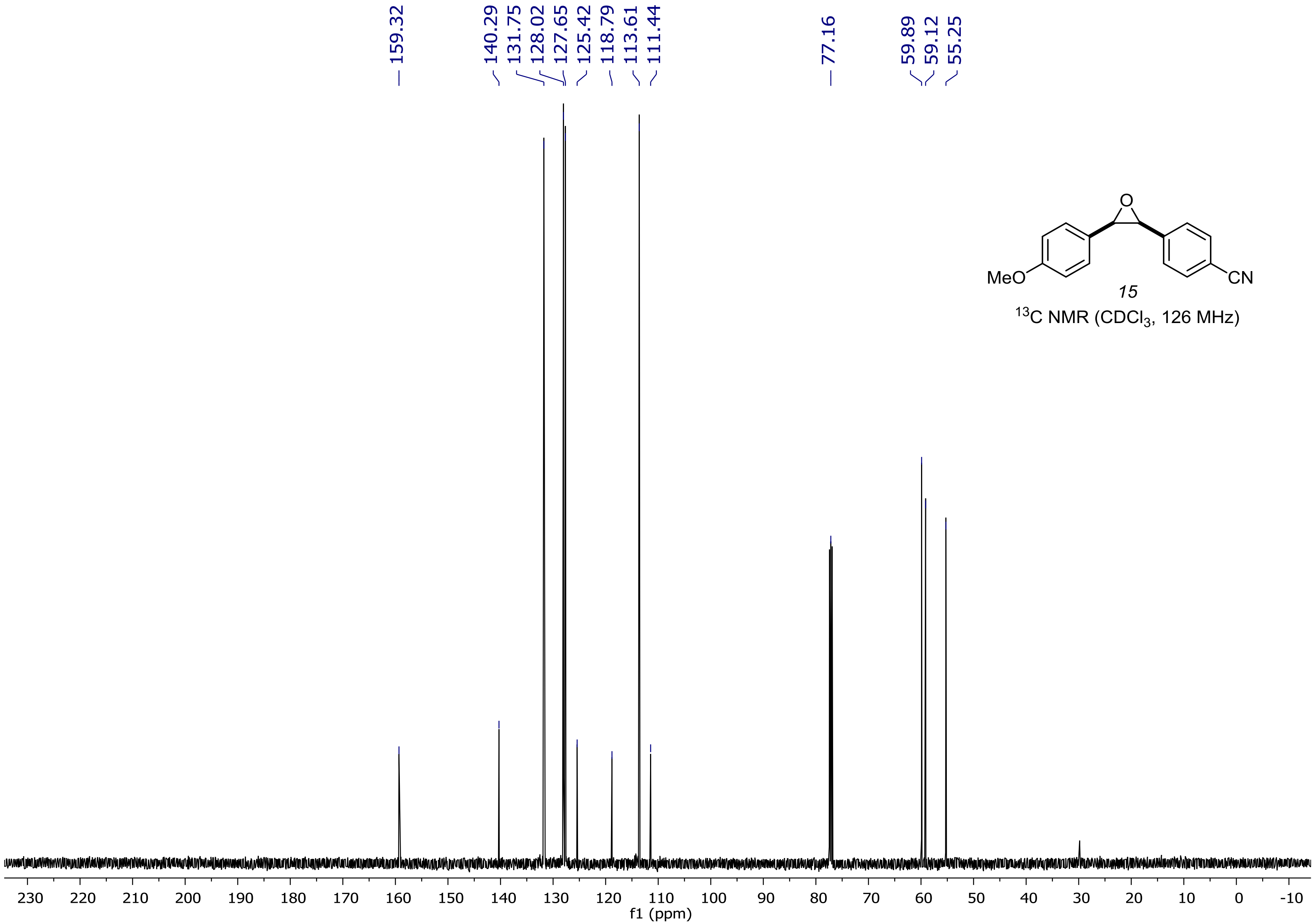


*15*

$^{13}$C NMR (CDCl$_3$, 126 MHz)

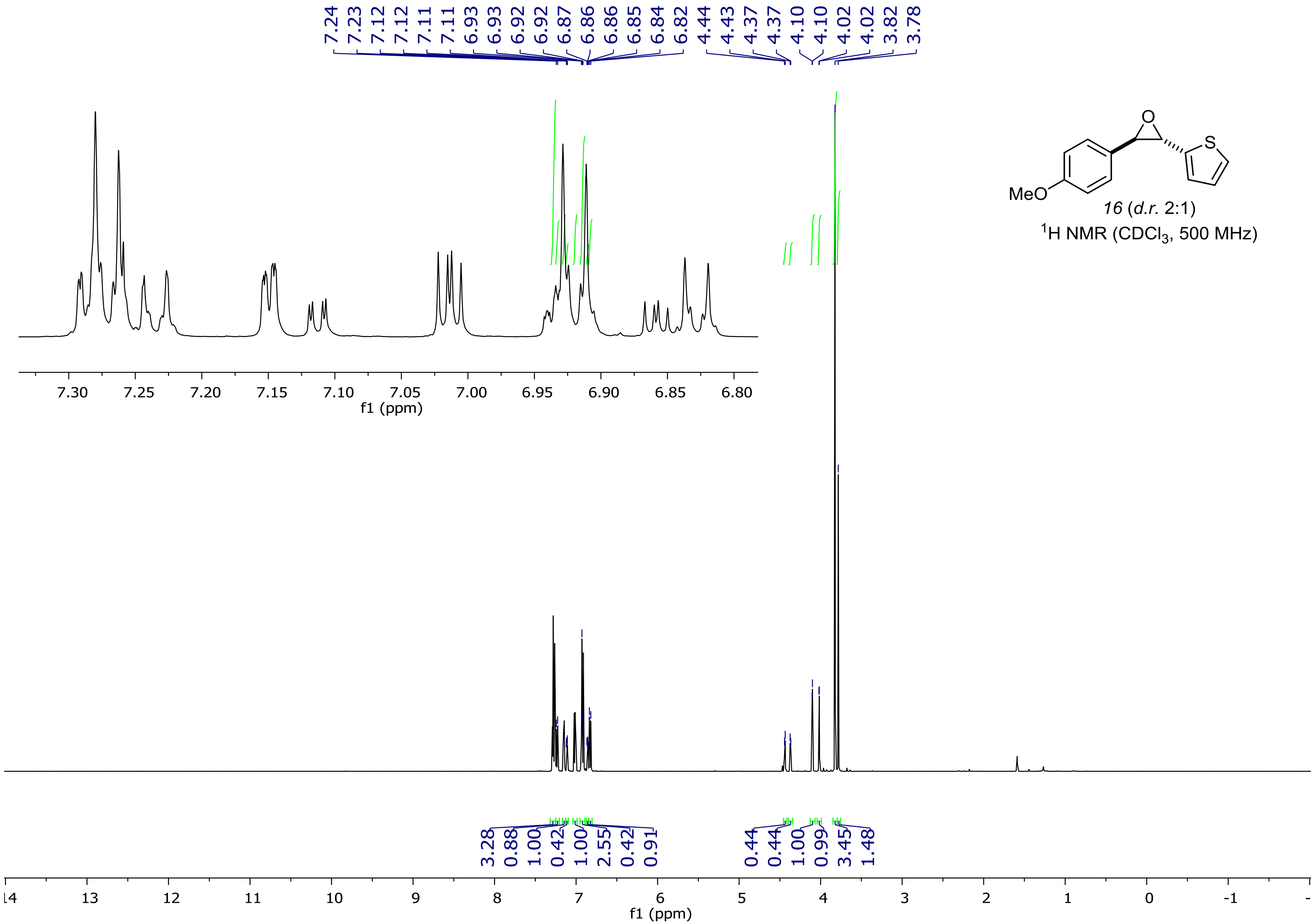


*16* (*d.r.* 2:1)

$^1$H NMR ($CDCl_3$, 500 MHz)

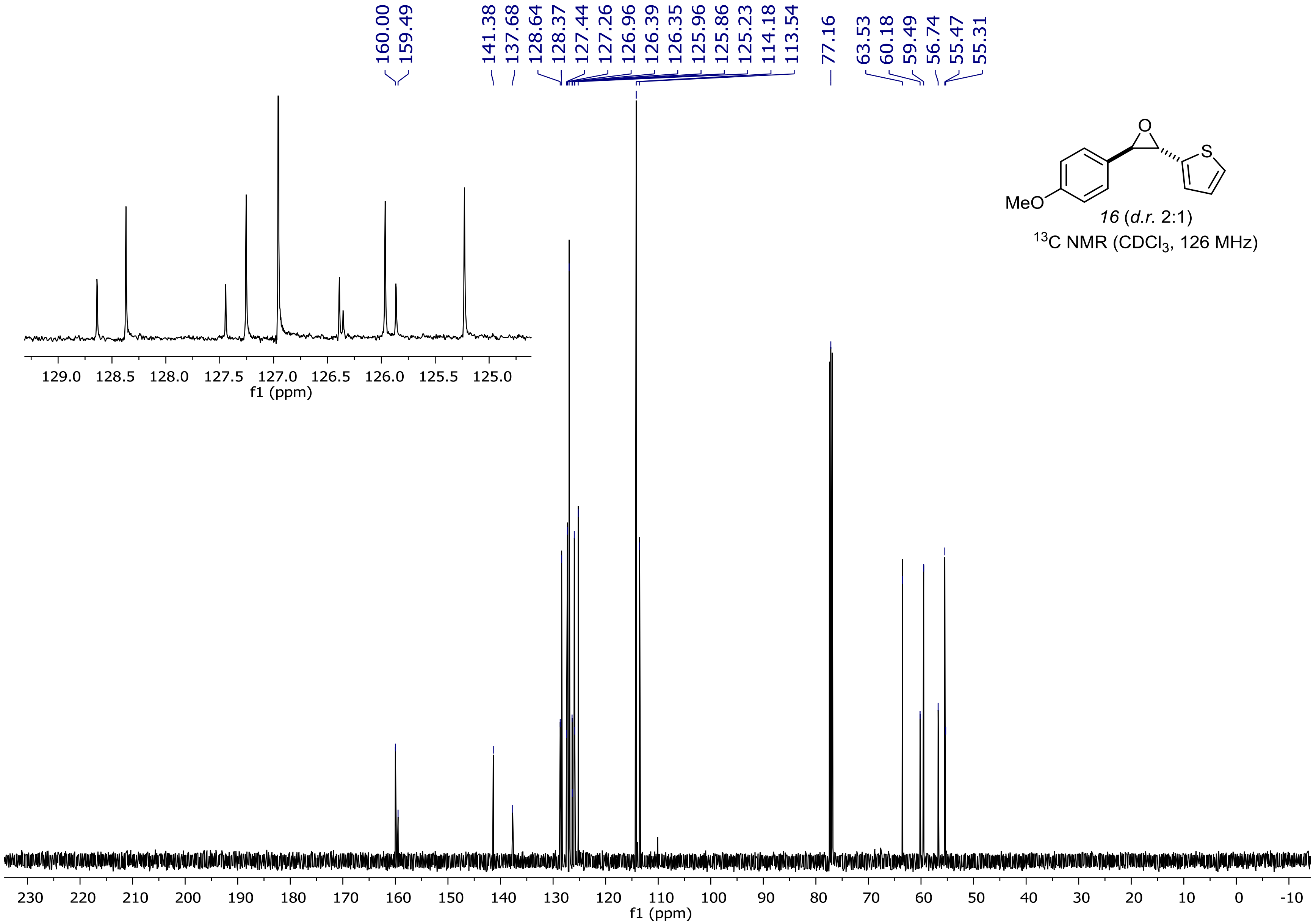


*16* (*d.r.* 2:1)

$^{13}C$ NMR ($CDCl_3$, 126 MHz)

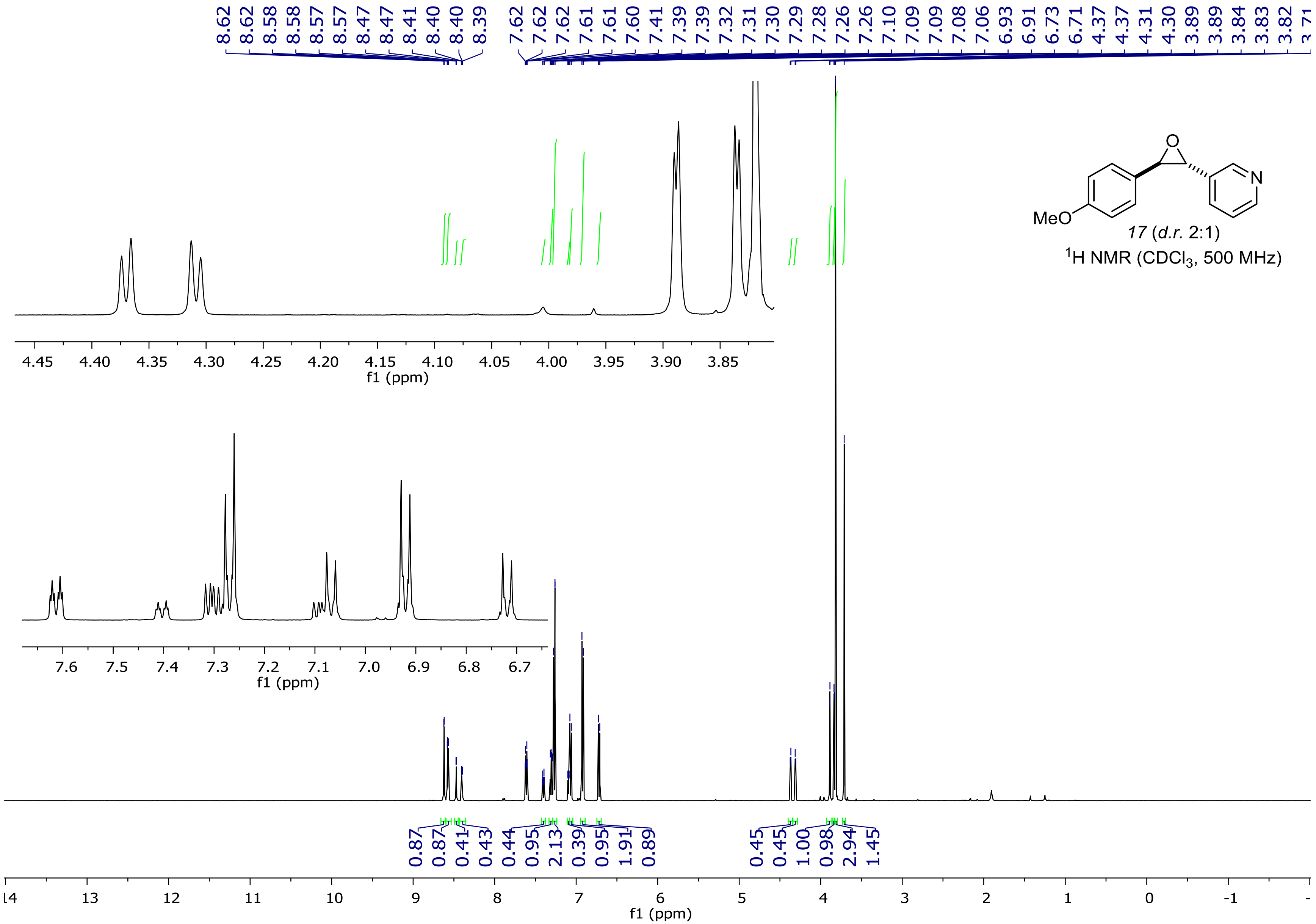
8.62 8.62 8.58 8.58 8.57 8.57 8.47 8.47 8.41 8.40 8.40 8.39 7.62 7.62 7.62 7.61 7.61 7.60 7.41 7.39 7.39 7.32 7.31 7.30 7.29 7.28 7.26 7.26 7.10 7.09 7.09 7.08 7.06 6.93 6.91 6.73 6.71 4.37 4.37 4.31 4.30 3.89 3.89 3.84 3.83 3.82 3.71
MeO
O
N
17 (d.r. 2:1)
1H NMR (CDCl3, 500 MHz)
4.45 4.40 4.35 4.30 4.25 4.20 4.15 4.10 4.05 4.00 3.95 3.90 3.85
f1 (ppm)
7.6 7.5 7.4 7.3 7.2 7.1 7.0 6.9 6.8 6.7
f1 (ppm)
0.87 0.87 0.41 0.43 0.44 0.95 2.13 0.39 0.95 1.91 0.89 0.45 0.45 1.00 0.98 2.94 1.45
13 12 11 10 9 8 7 6 5 4 3 2 1 0 -1
f1 (ppm)

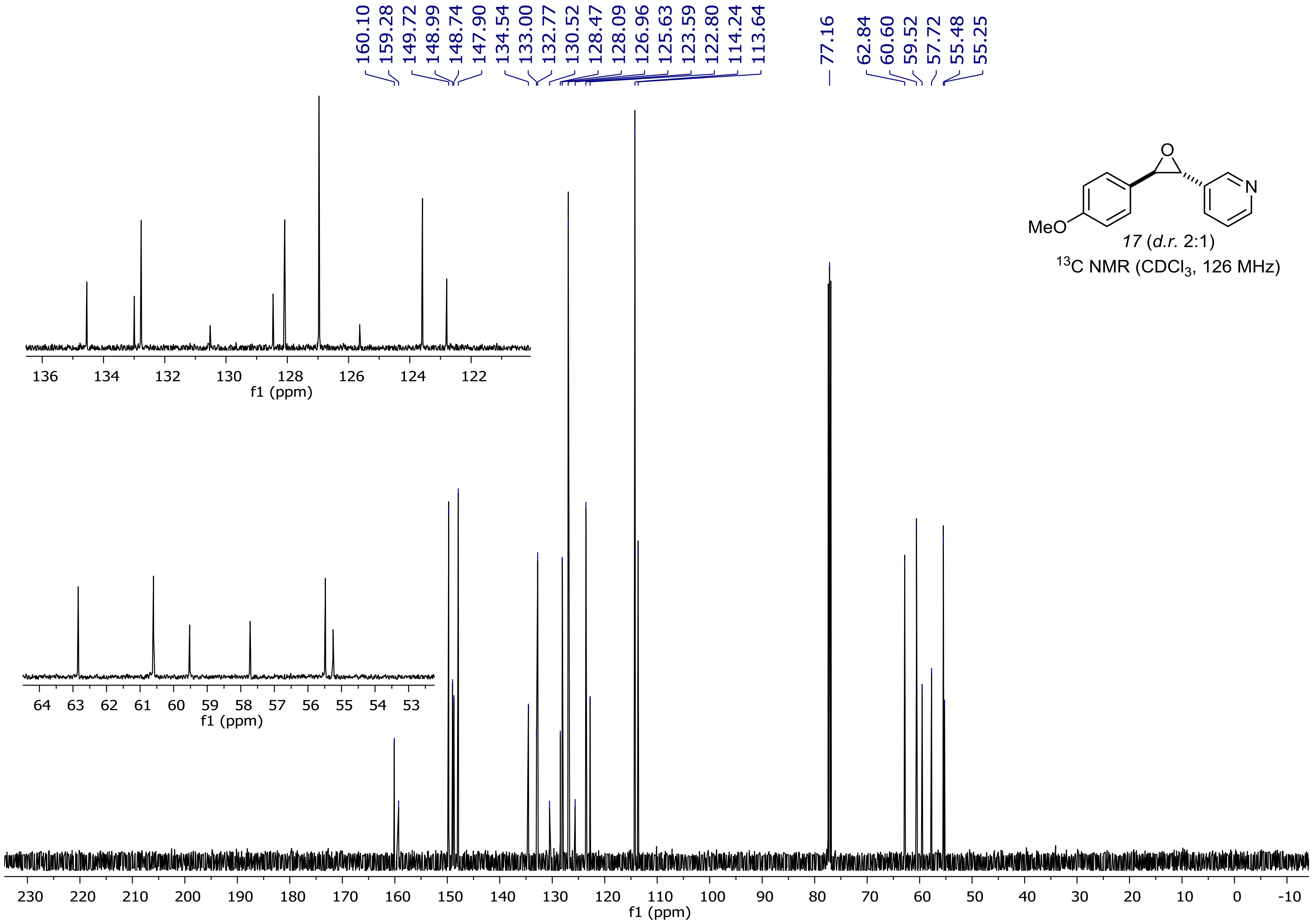
160.10
159.28
149.72
148.99
148.74
147.90
134.54
133.00
132.77
130.52
128.47
128.09
126.96
125.63
123.59
122.80
114.24
113.64
77.16
62.84
60.60
59.52
57.72
55.48
55.25
MeO
O
N
17 (d.r. 2:1)
13C NMR (CDCl3, 126 MHz)
f1 (ppm)

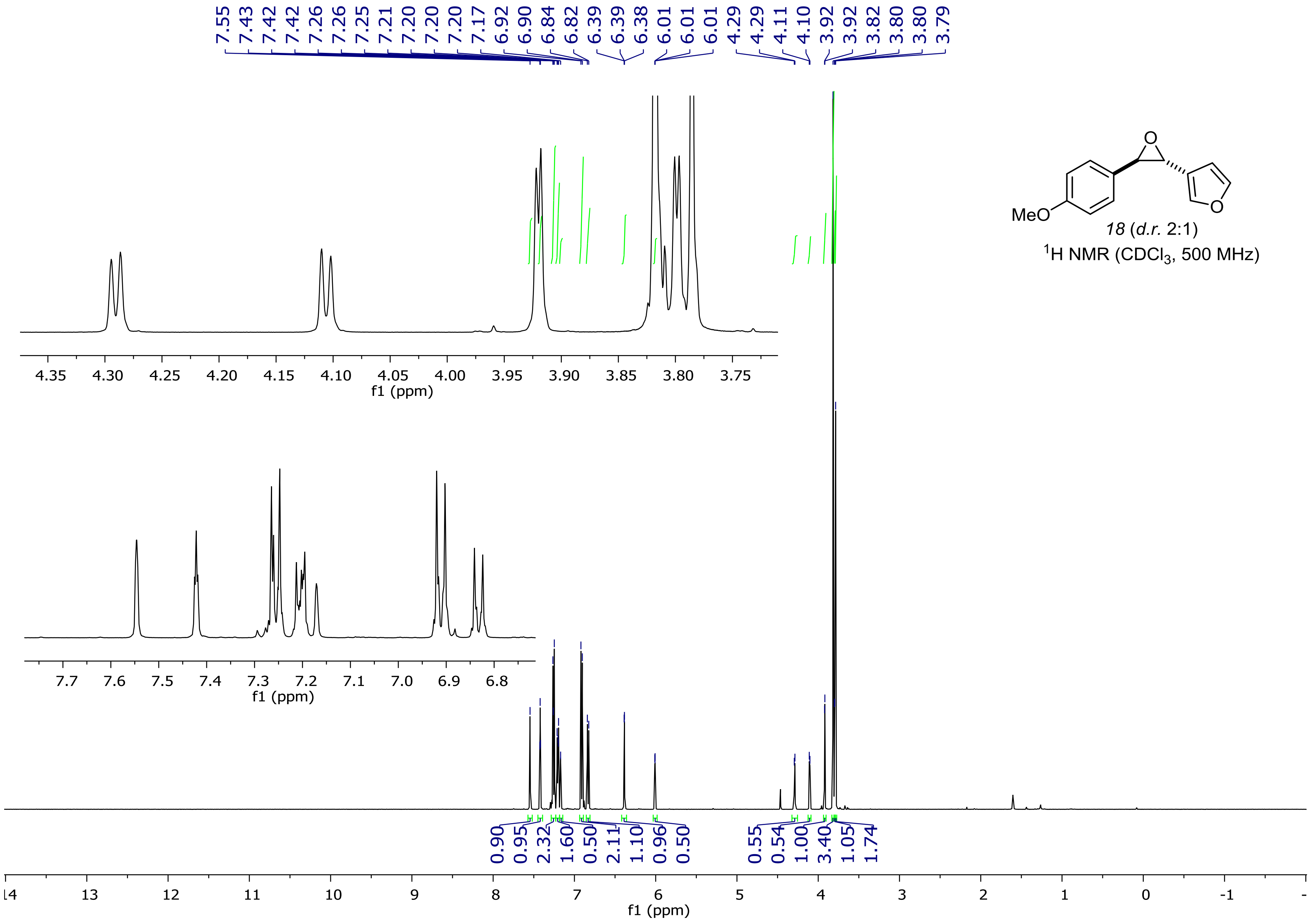
7.55 7.43 7.42 7.42 7.26 7.26 7.25 7.21 7.20 7.20 7.20 7.17 6.92 6.90 6.84 6.82 6.39 6.39 6.38 6.01 6.01 6.01 4.29 4.29 4.11 4.10 3.92 3.92 3.82 3.80 3.80 3.79
MeO
O
18 (d.r. 2:1)
1H NMR (CDCl3, 500 MHz)
0.90 0.95 2.32 1.60 0.50 2.11 1.10 0.96 0.50 0.55 0.54 1.00 3.40 1.05 1.74
f1 (ppm)

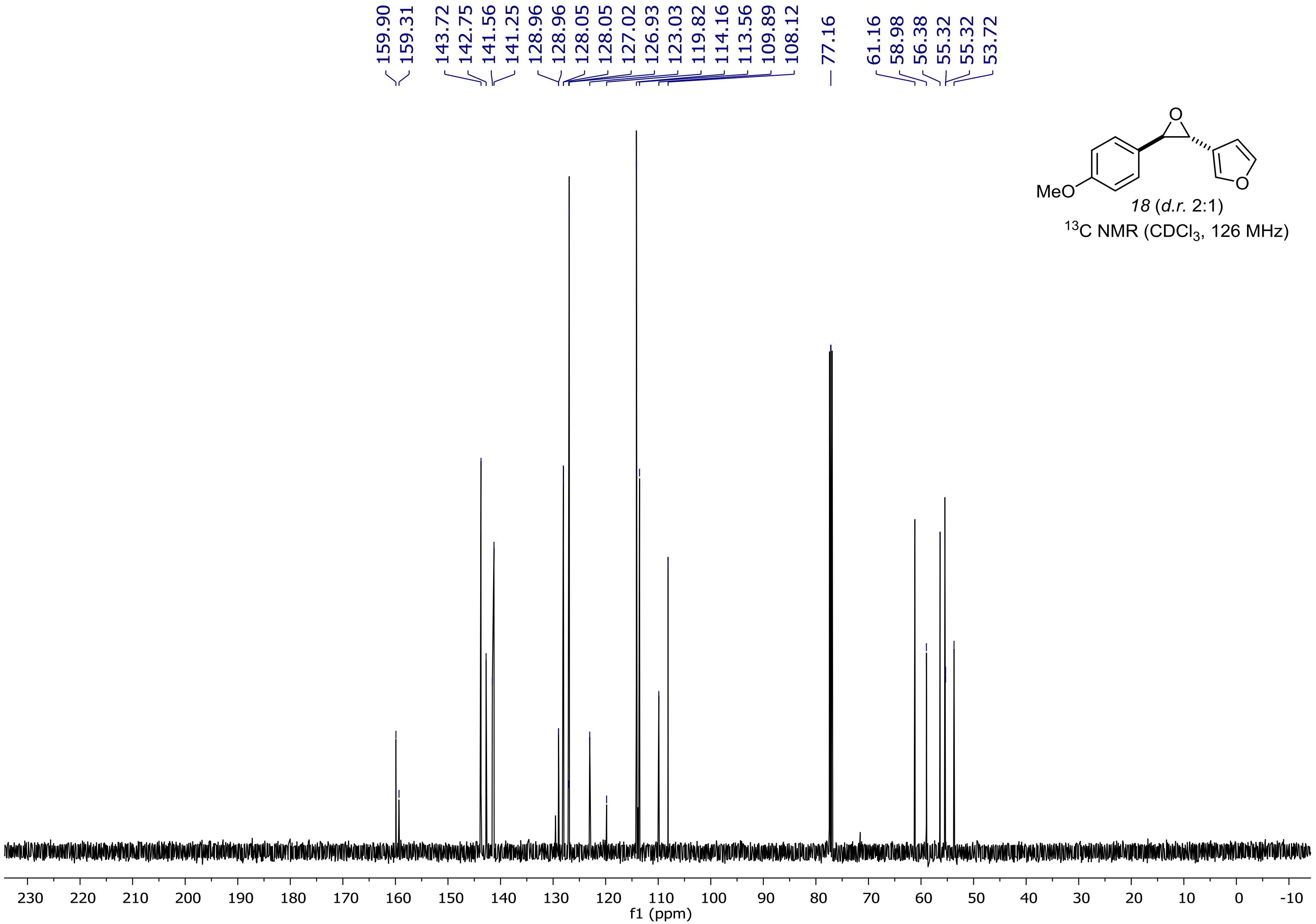
159.90
159.31
143.72
142.75
141.56
141.25
128.96
128.96
128.05
128.05
127.02
126.93
123.03
119.82
114.16
113.56
109.89
108.12
77.16
61.16
58.98
56.38
55.32
55.32
53.72
MeO
O
18 (d.r. 2:1)
13C NMR (CDCl3, 126 MHz)
230 220 210 200 190 180 170 160 150 140 130 120 110 100 90 80 70 60 50 40 30 20 10 0 -10
f1 (ppm)

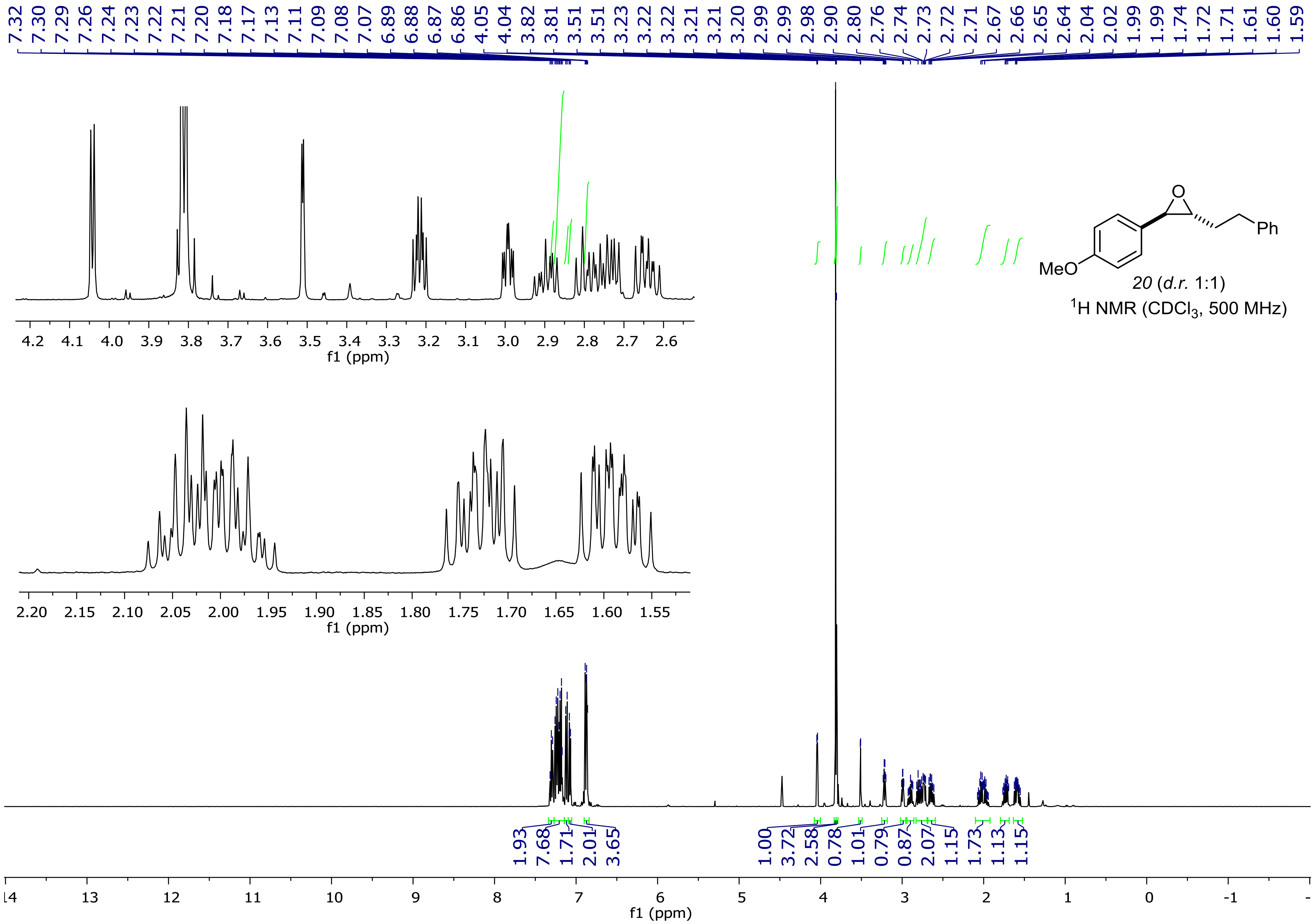
MeO
Ph
O
20 (d.r. 1:1)
¹H NMR (CDCl₃, 500 MHz)
f1 (ppm)

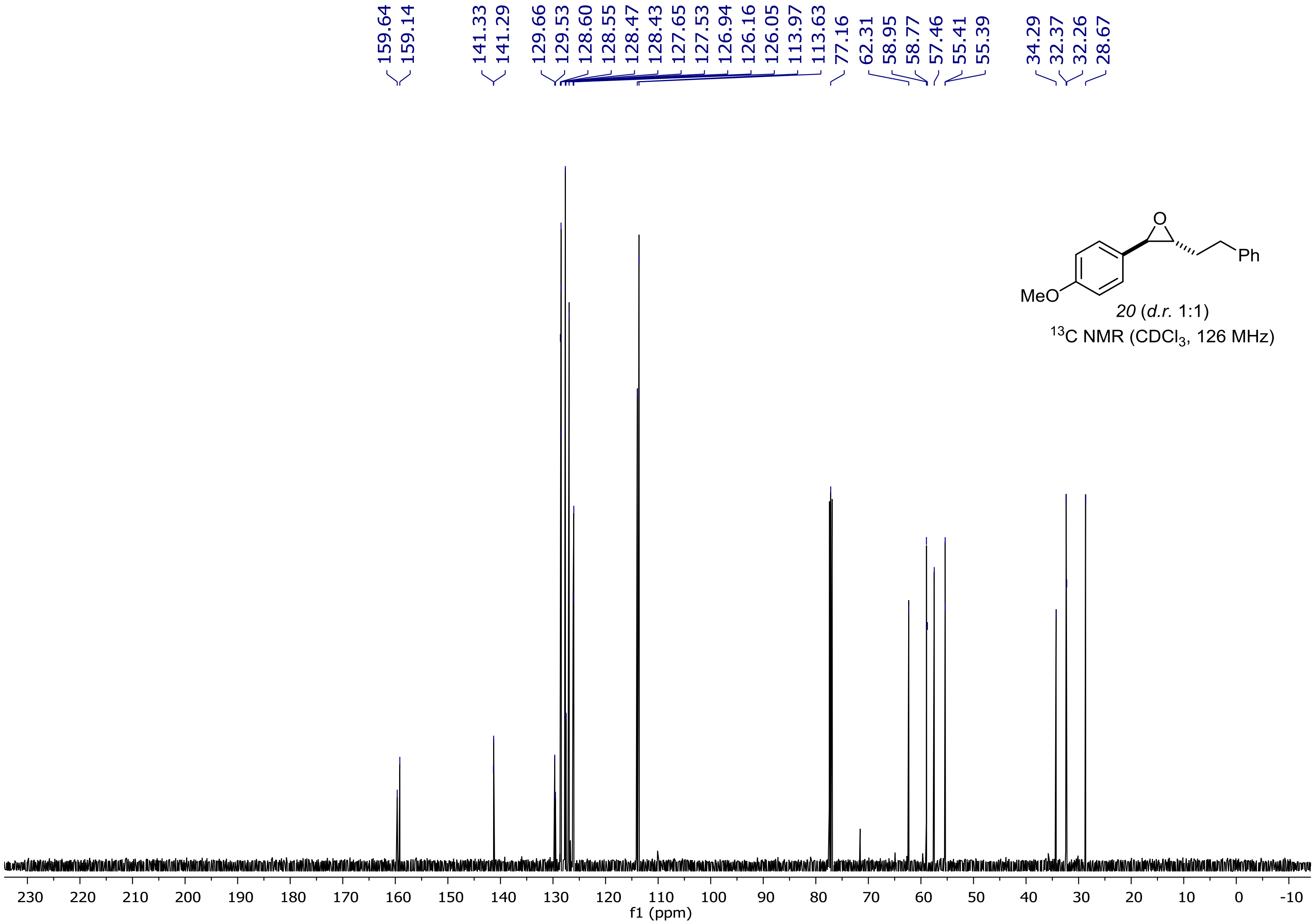
159.64
159.14
141.33
141.29
129.66
129.53
128.60
128.55
128.47
128.43
127.65
127.53
126.94
126.16
126.05
113.97
113.63
77.16
62.31
58.95
58.77
57.46
55.41
55.39
34.29
32.37
32.26
28.67
O
Ph
MeO
20 (d.r. 1:1)
13C NMR (CDCl3, 126 MHz)
230 220 210 200 190 180 170 160 150 140 130 120 110 100 90 80 70 60 50 40 30 20 10 0 -10
f1 (ppm)

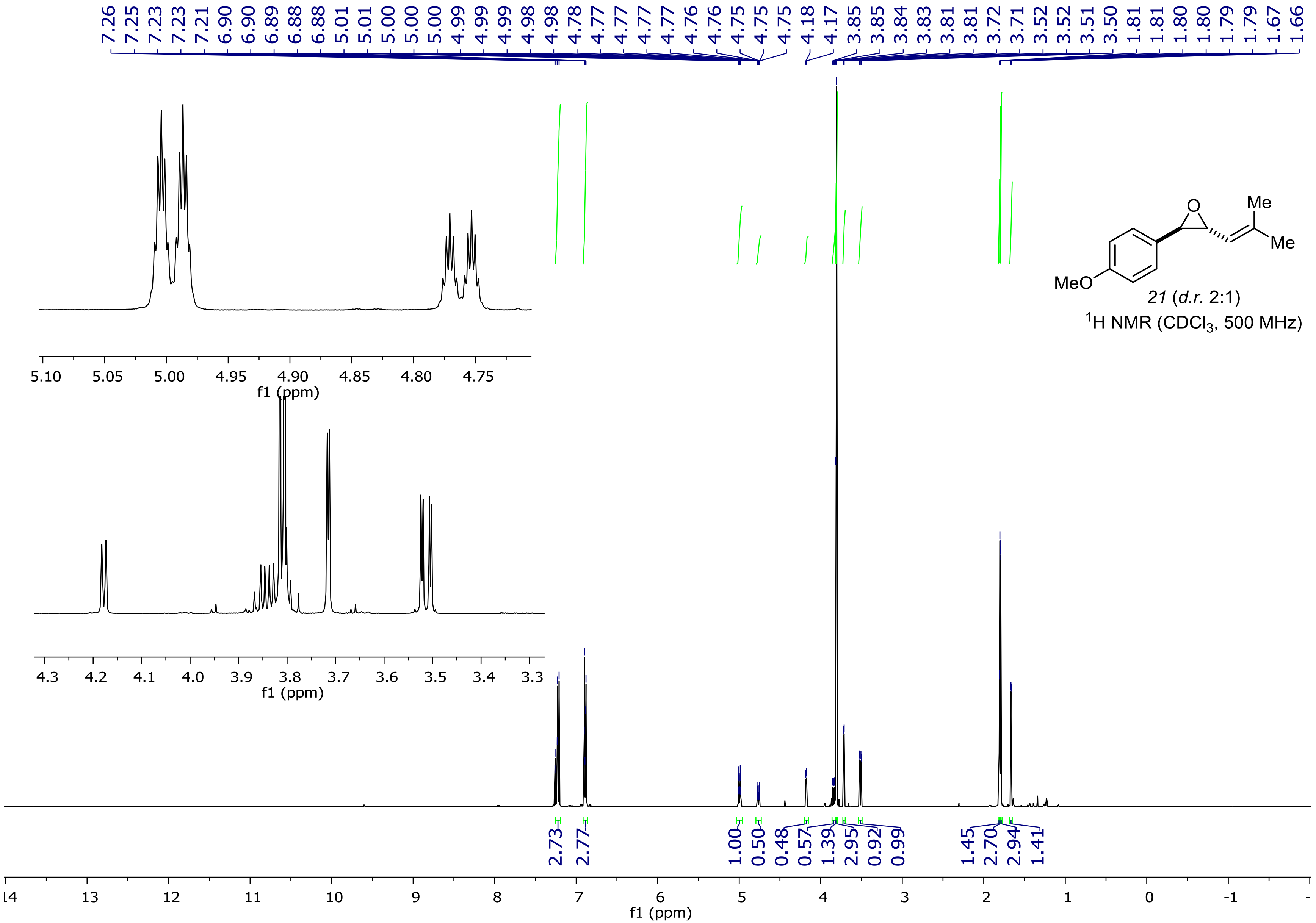
7.26 7.25 7.23 7.23 7.21 6.90 6.90 6.89 6.88 6.88 5.01 5.01 5.00 5.00 4.99 4.99 4.99 4.98 4.98 4.78 4.77 4.77 4.77 4.77 4.76 4.76 4.75 4.75 4.75 4.18 4.17 3.85 3.85 3.84 3.83 3.81 3.81 3.72 3.71 3.52 3.52 3.51 3.50 1.81 1.81 1.80 1.80 1.79 1.79 1.67 1.66
MeO
O
Me
Me
21 (d.r. 2:1)
$^1$H NMR ($CDCl_3$, 500 MHz)
2.73 2.77 1.00 0.50 0.48 0.57 1.39 2.95 0.92 0.99 1.45 2.70 2.94 1.41
f1 (ppm)

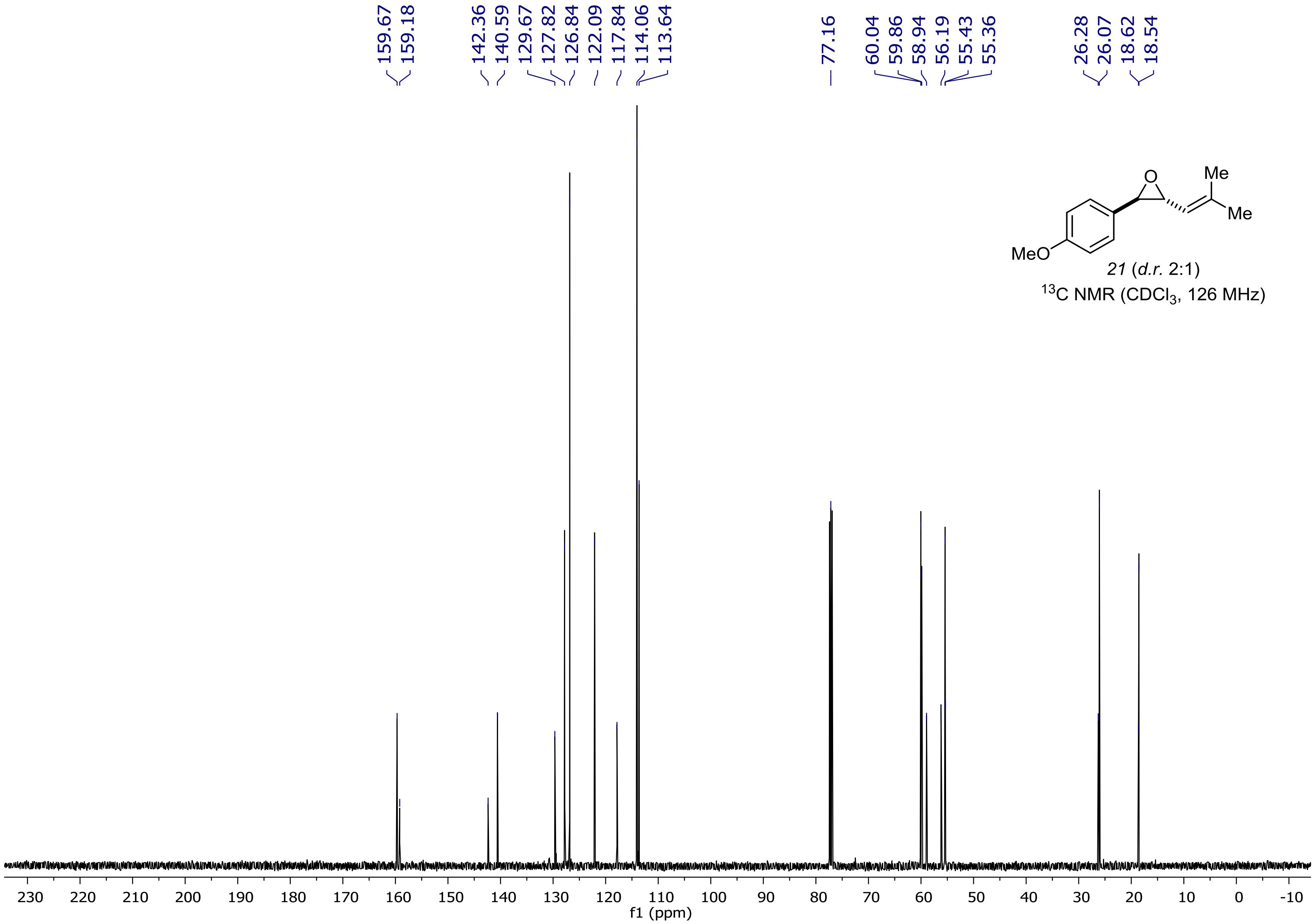


*21* (*d.r.* 2:1)
$^{13}$C NMR ($CDCl_3$, 126 MHz)

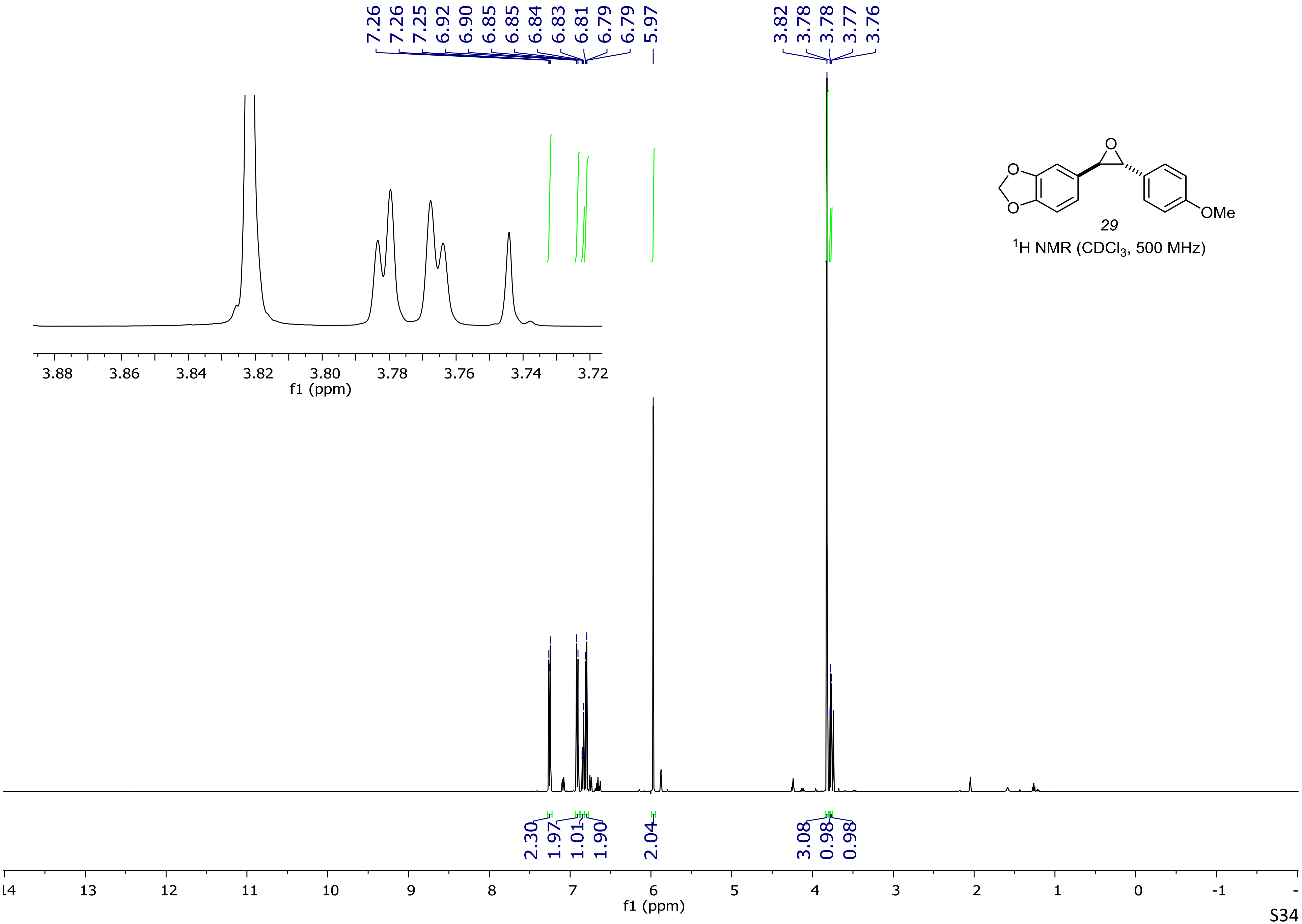


29

$^{1}$H NMR ($CDCl_3$, 500 MHz)

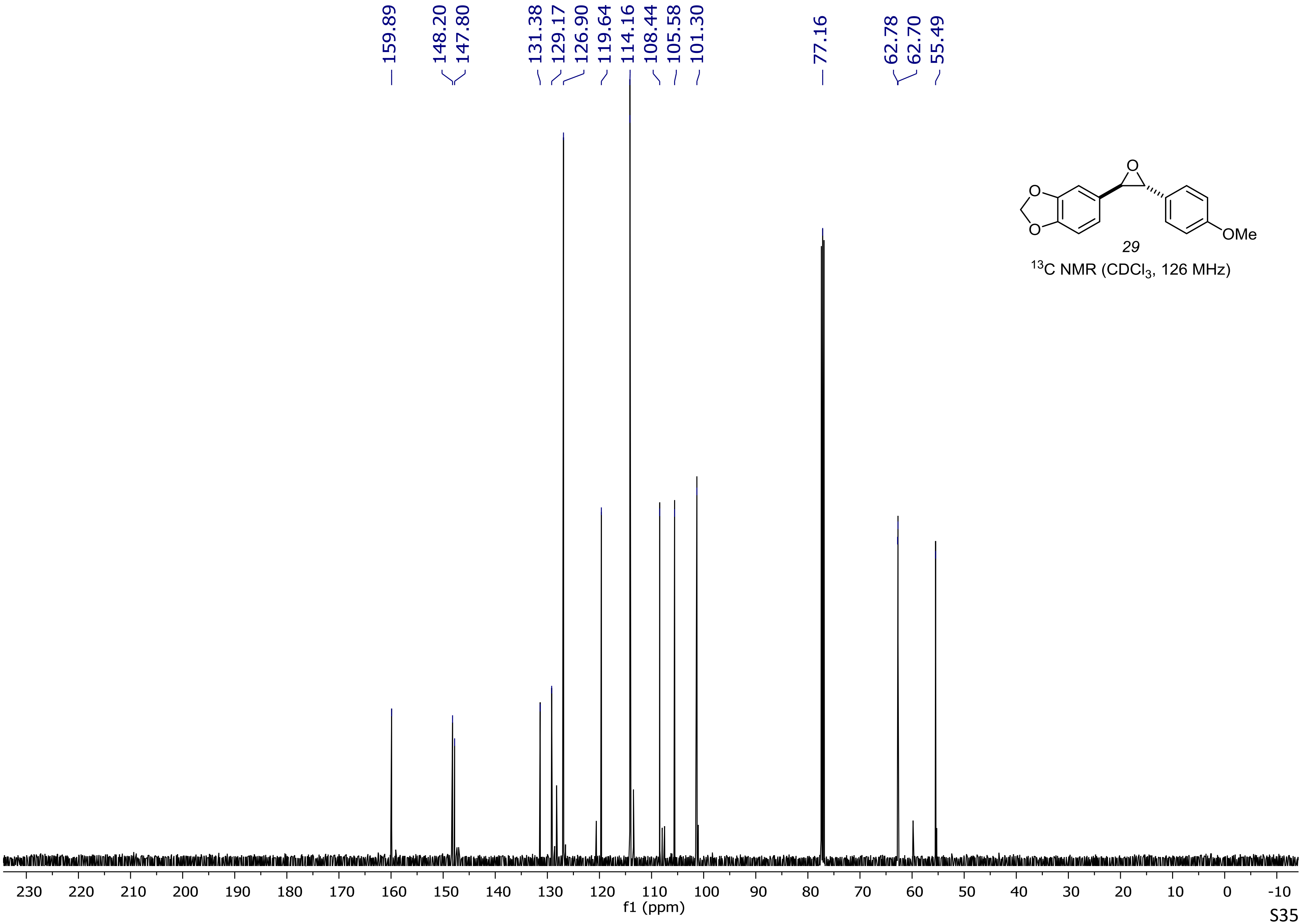
159.89
148.20
147.80
131.38
129.17
126.90
119.64
114.16
108.44
105.58
101.30
77.16
62.78
62.70
55.49
O
O
O
OMe
29
13C NMR (CDCl3, 126 MHz)
230 220 210 200 190 180 170 160 150 140 130 120 110 100 90 80 70 60 50 40 30 20 10 0 -10
f1 (ppm)

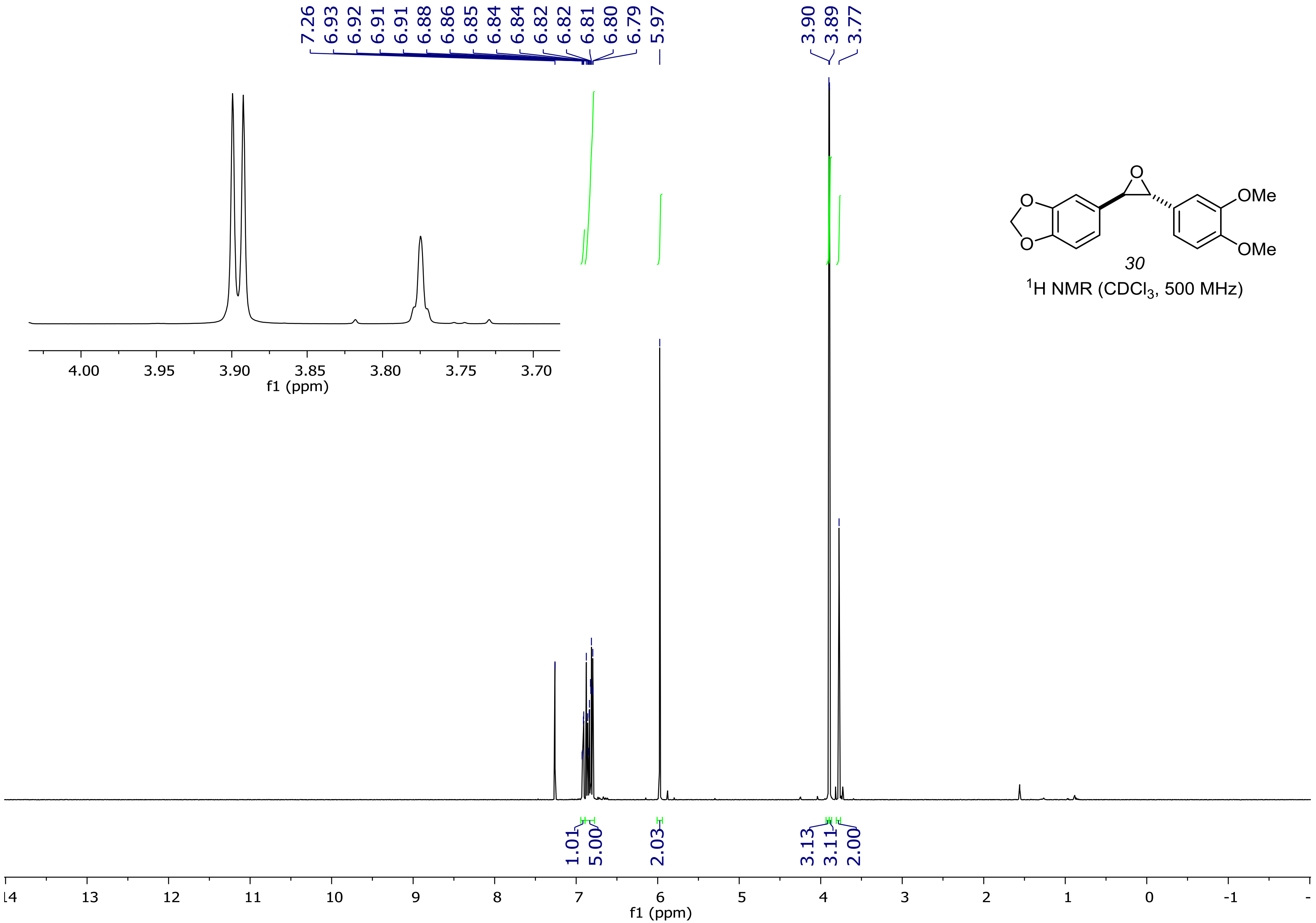
7.26
6.93
6.92
6.91
6.91
6.88
6.86
6.85
6.84
6.84
6.82
6.82
6.81
6.80
6.79
5.97
3.90
3.89
3.77
OMe
OMe
O
O
O
30
$^1$H NMR ($CDCl_3$, 500 MHz)
4.00
3.95
3.90
3.85
3.80
3.75
3.70
f1 (ppm)
1.01
5.00
2.03
3.13
3.11
2.00
14
13
12
11
10
9
8
7
6
5
4
3
2
1
0
-1
f1 (ppm)

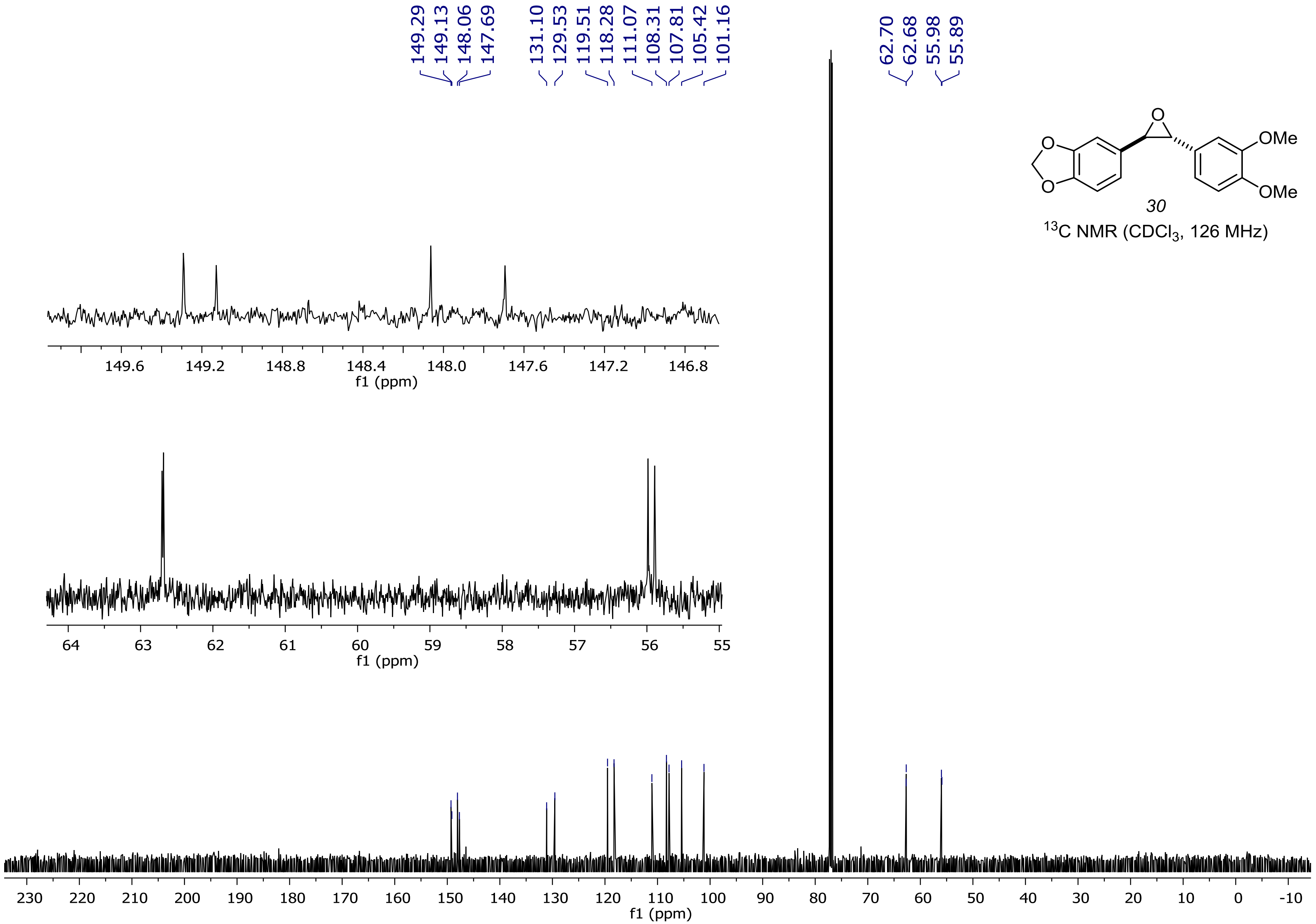
149.29
149.13
148.06
147.69
131.10
129.53
119.51
118.28
111.07
108.31
107.81
105.42
101.16
62.70
62.68
55.98
55.89
OMe
OMe
30
$^{13}C$ NMR ($CDCl_3$, 126 MHz)
f1 (ppm)

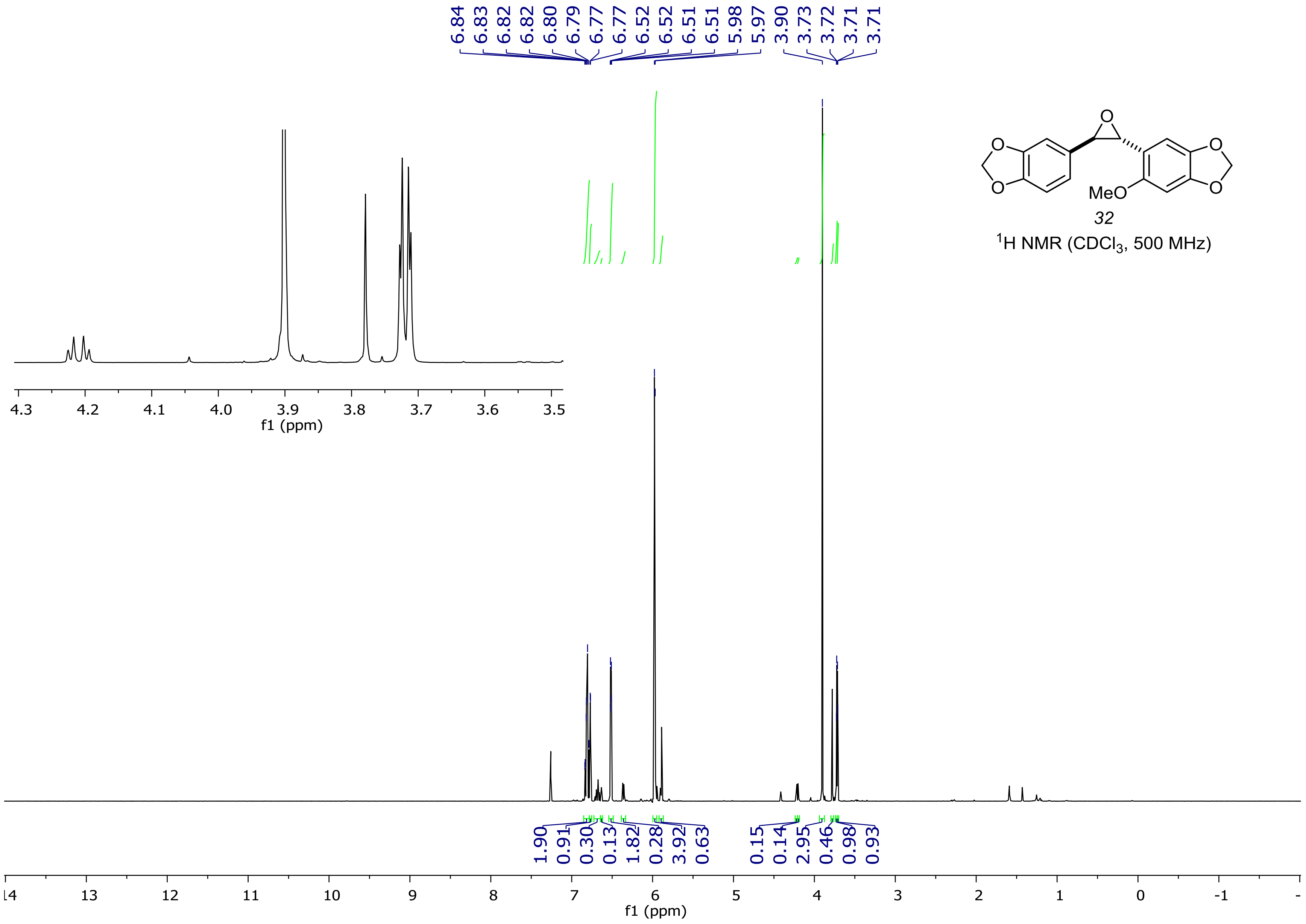


*32*

$^1$H NMR ($CDCl_3$, 500 MHz)

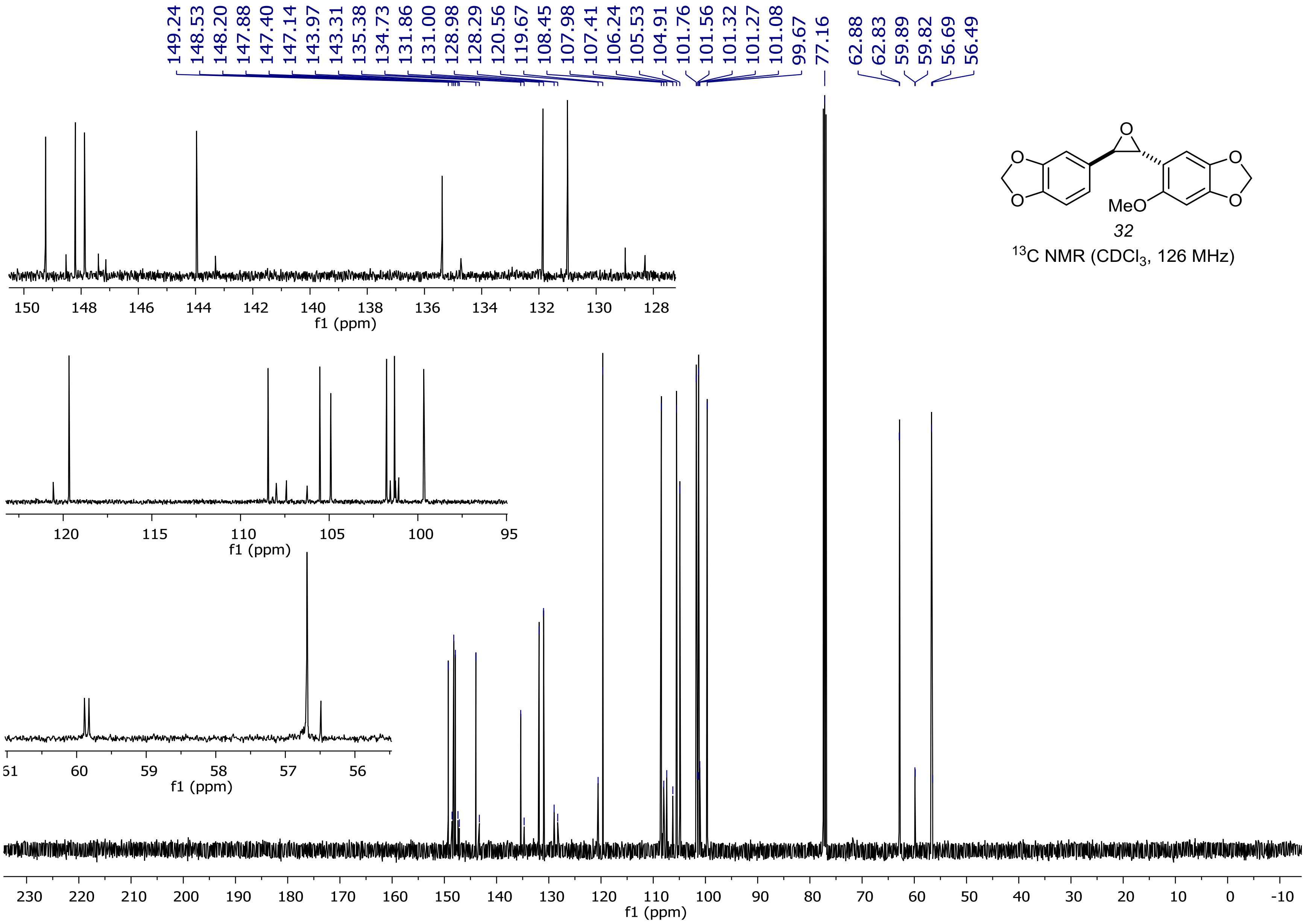


*32*

$^{13}C$ NMR ($CDCl_3$, 126 MHz)

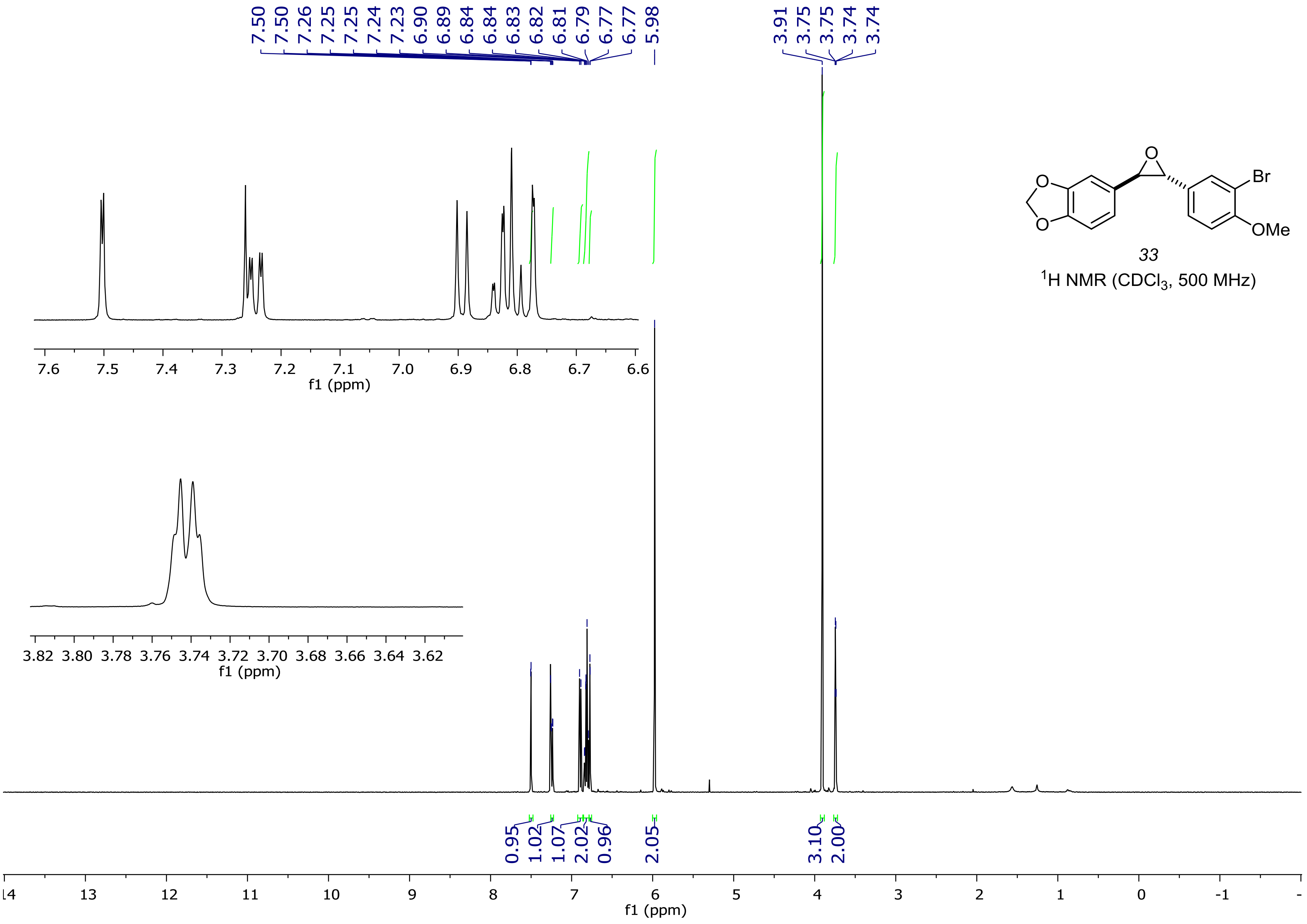

33

$^1$H NMR ($CDCl_3$, 500 MHz)

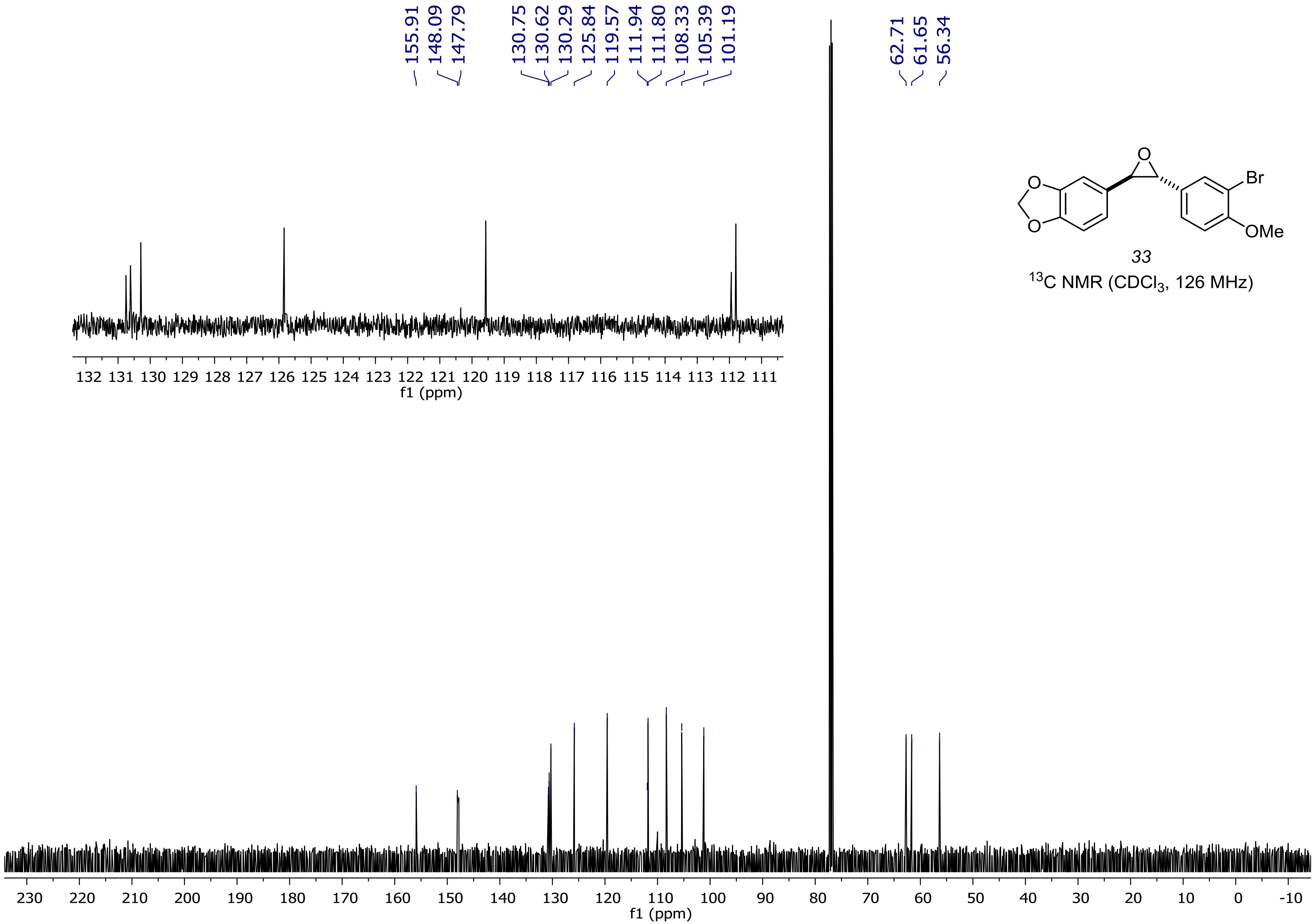


*33*

$^{13}$C NMR ($CDCl_3$, 126 MHz)

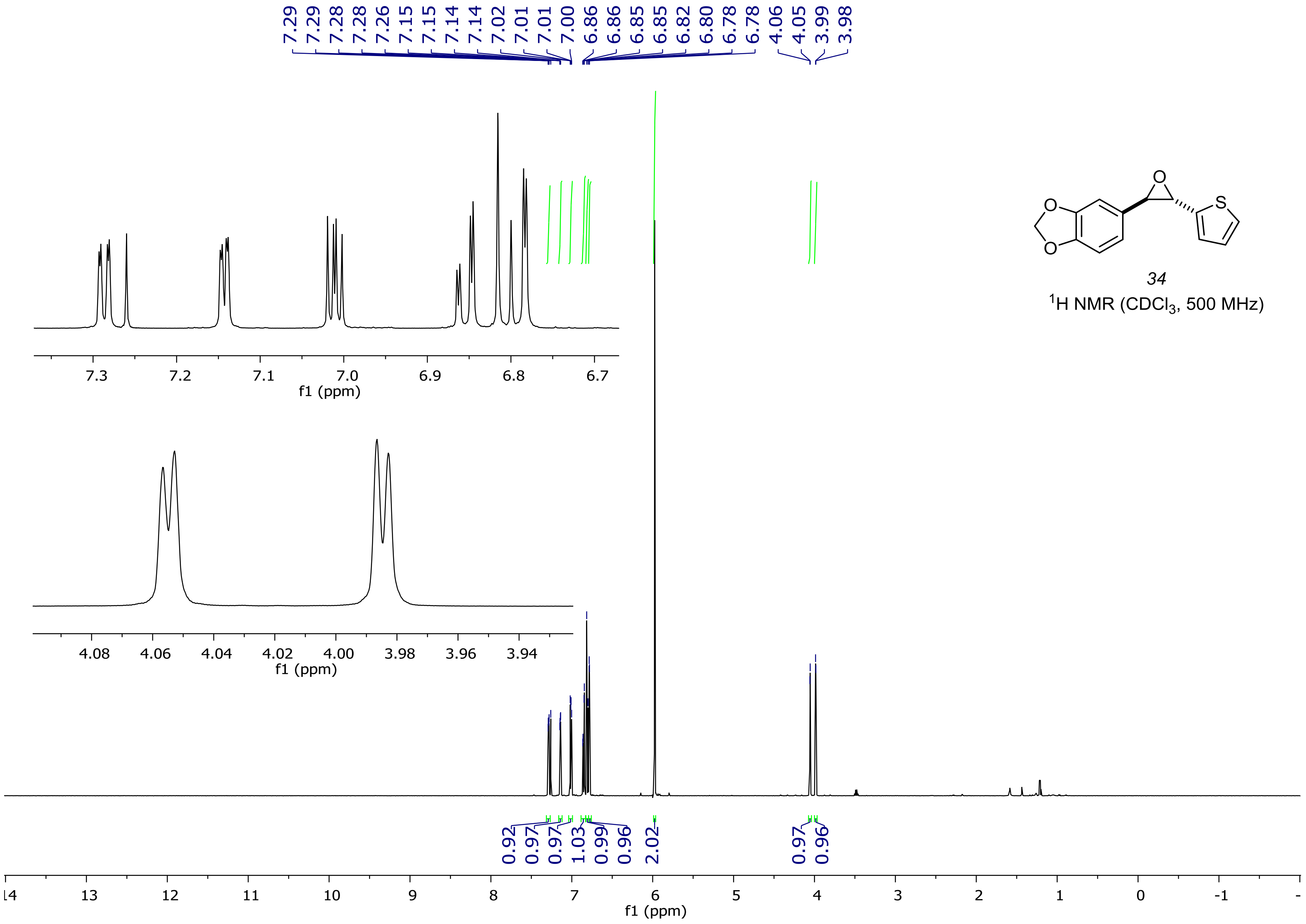


*34*

$^1$H NMR ($CDCl_3$, 500 MHz)

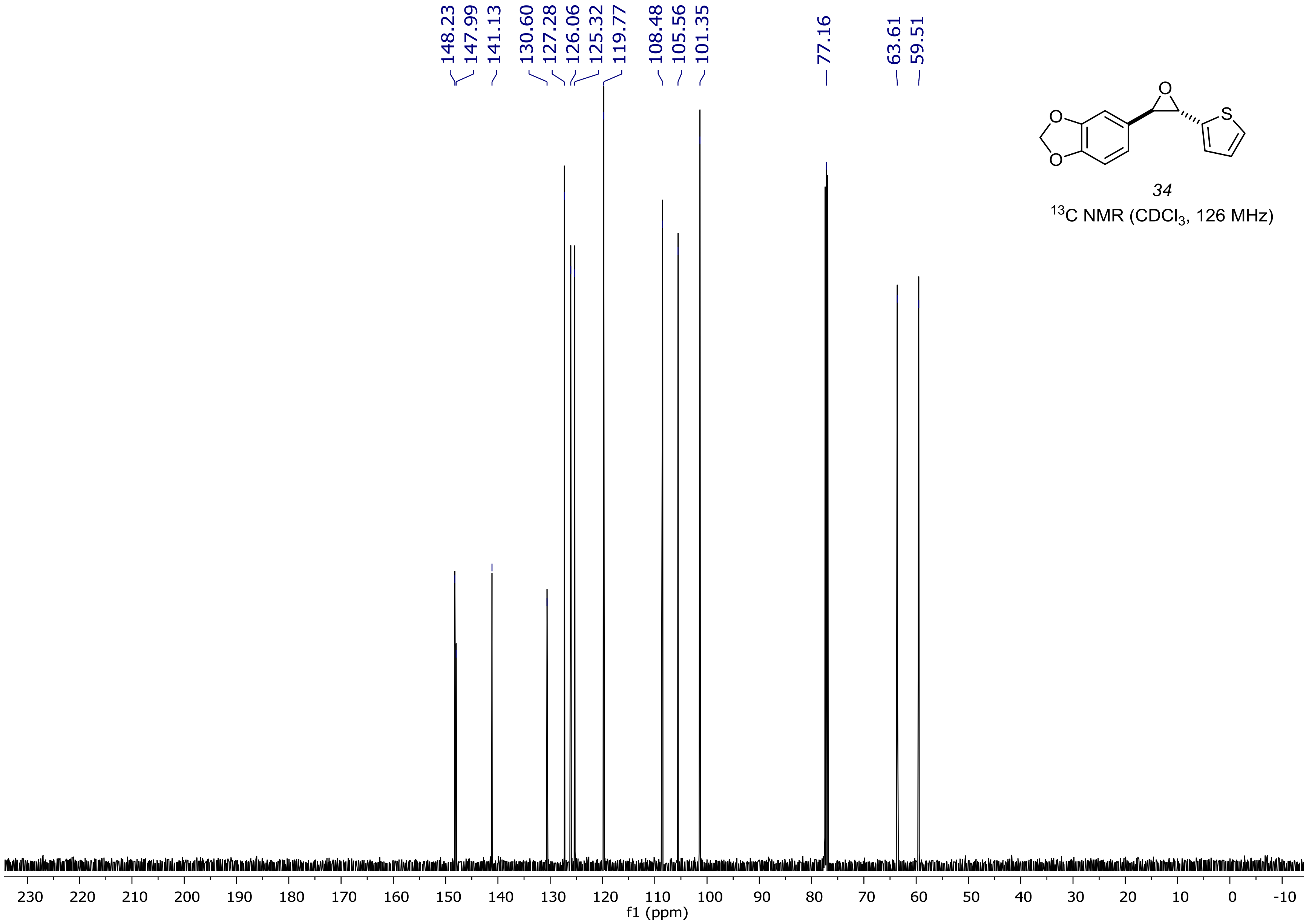


*34*

$^{13}$C NMR ($CDCl_3$, 126 MHz)

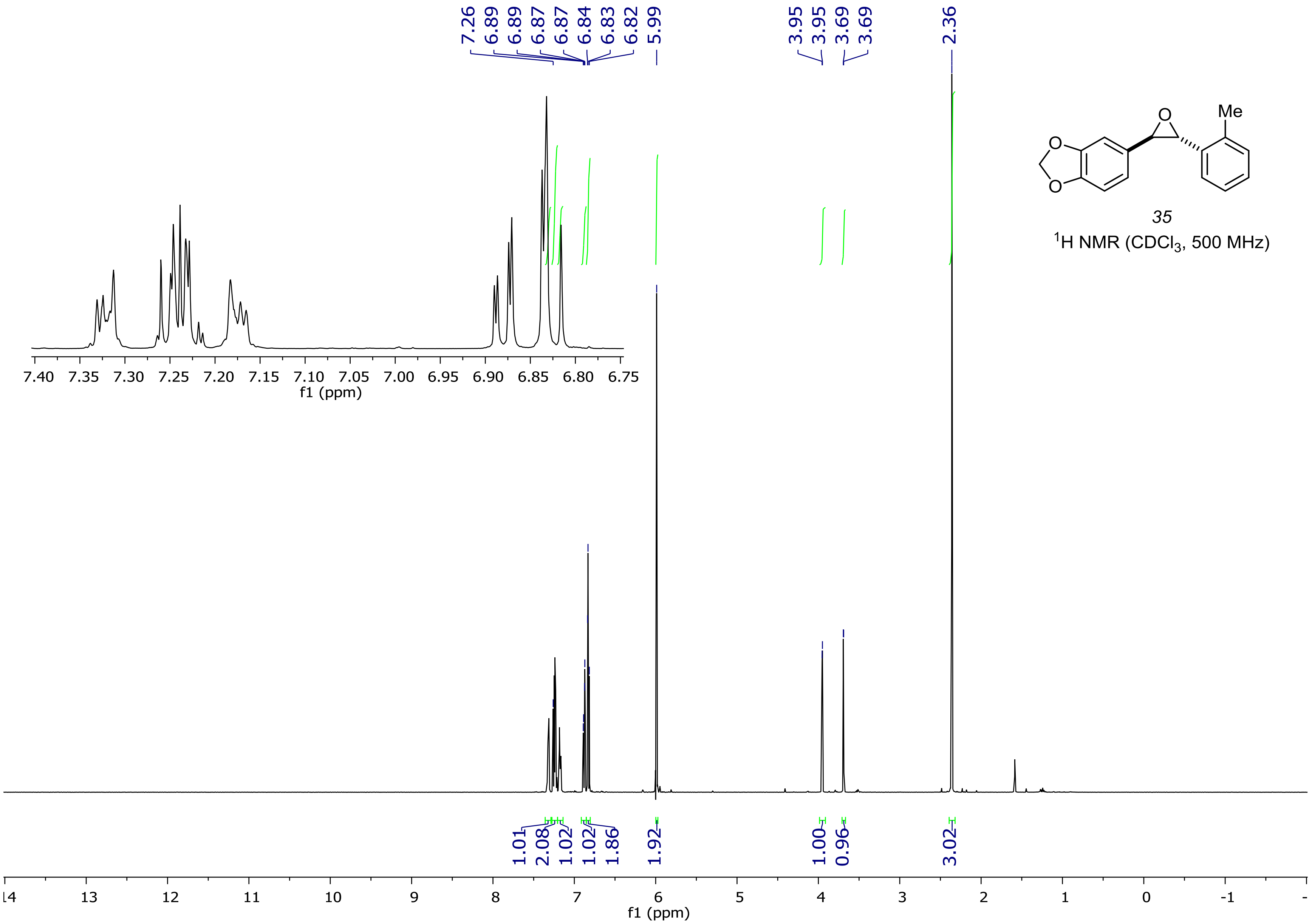


*35*

$^1$H NMR ($CDCl_3$, 500 MHz)

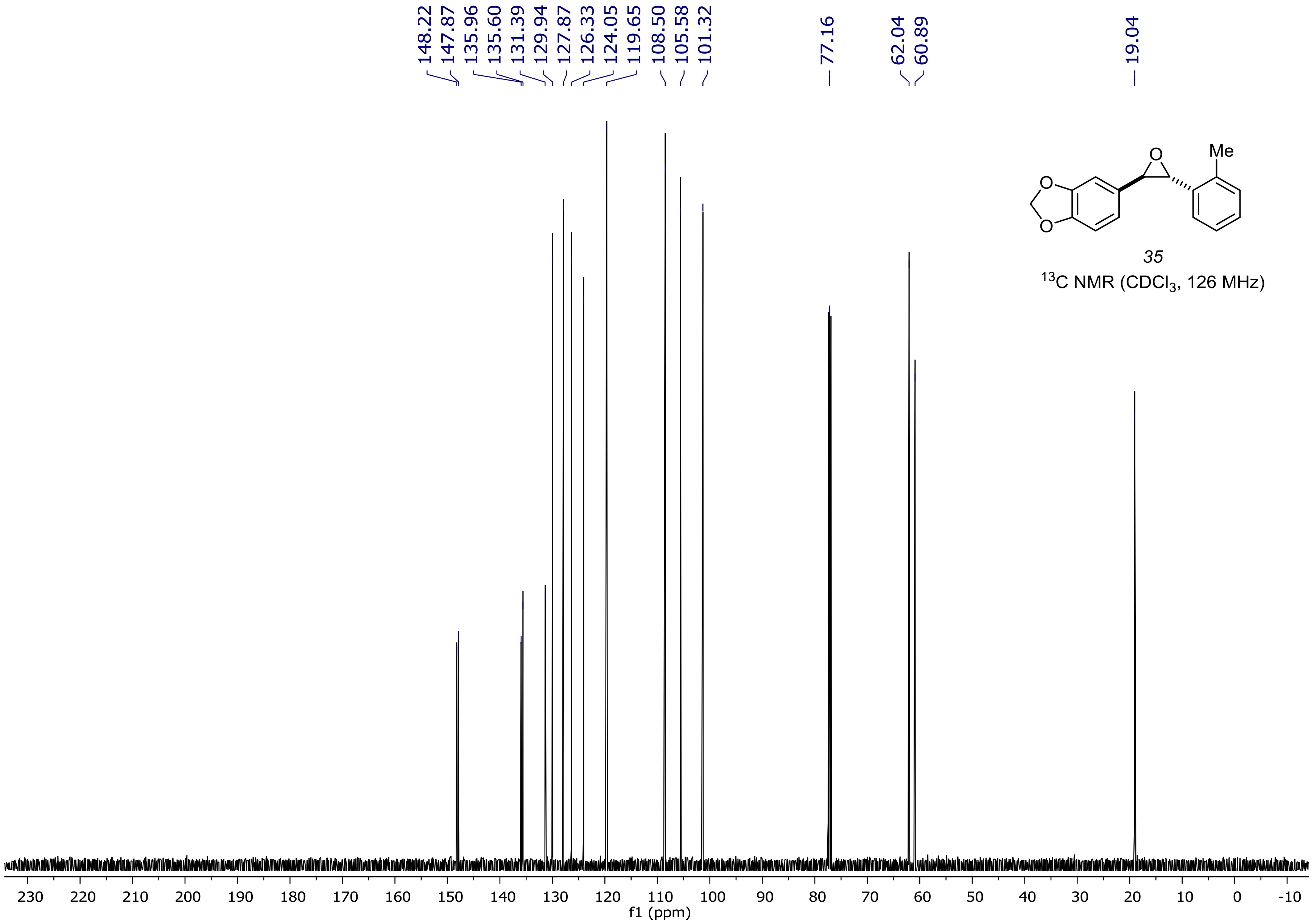
148.22
147.87
135.96
135.60
131.39
129.94
127.87
126.33
124.05
119.65
108.50
105.58
101.32
77.16
62.04
60.89
19.04
Me
O
35
$^{13}$C NMR ($CDCl_3$, 126 MHz)
230 220 210 200 190 180 170 160 150 140 130 120 110 100 90 80 70 60 50 40 30 20 10 0 -10
f1 (ppm)

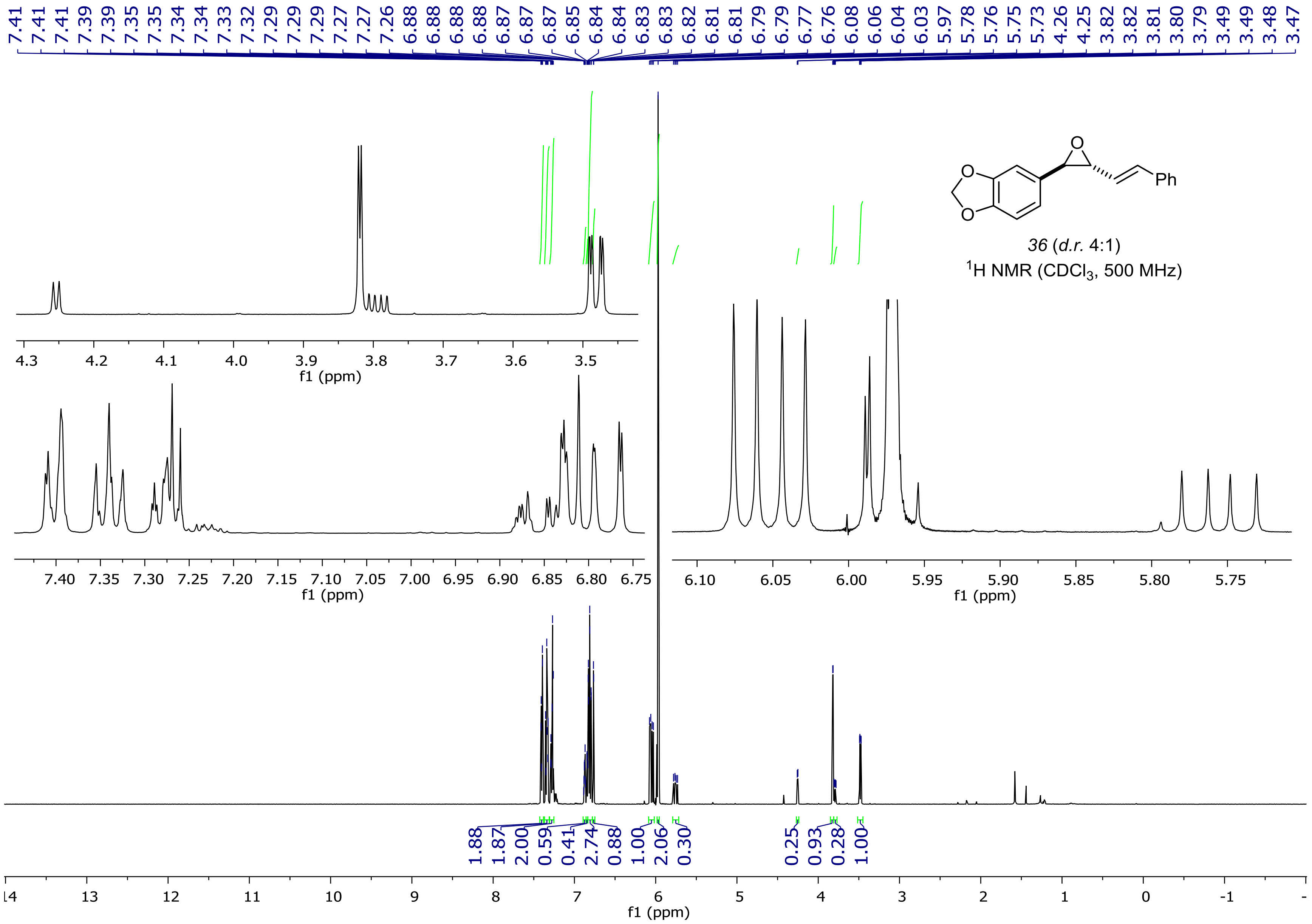
36 (d.r. 4:1)
$^1$H NMR (CDCl$_3$, 500 MHz)
Ph
f1 (ppm)

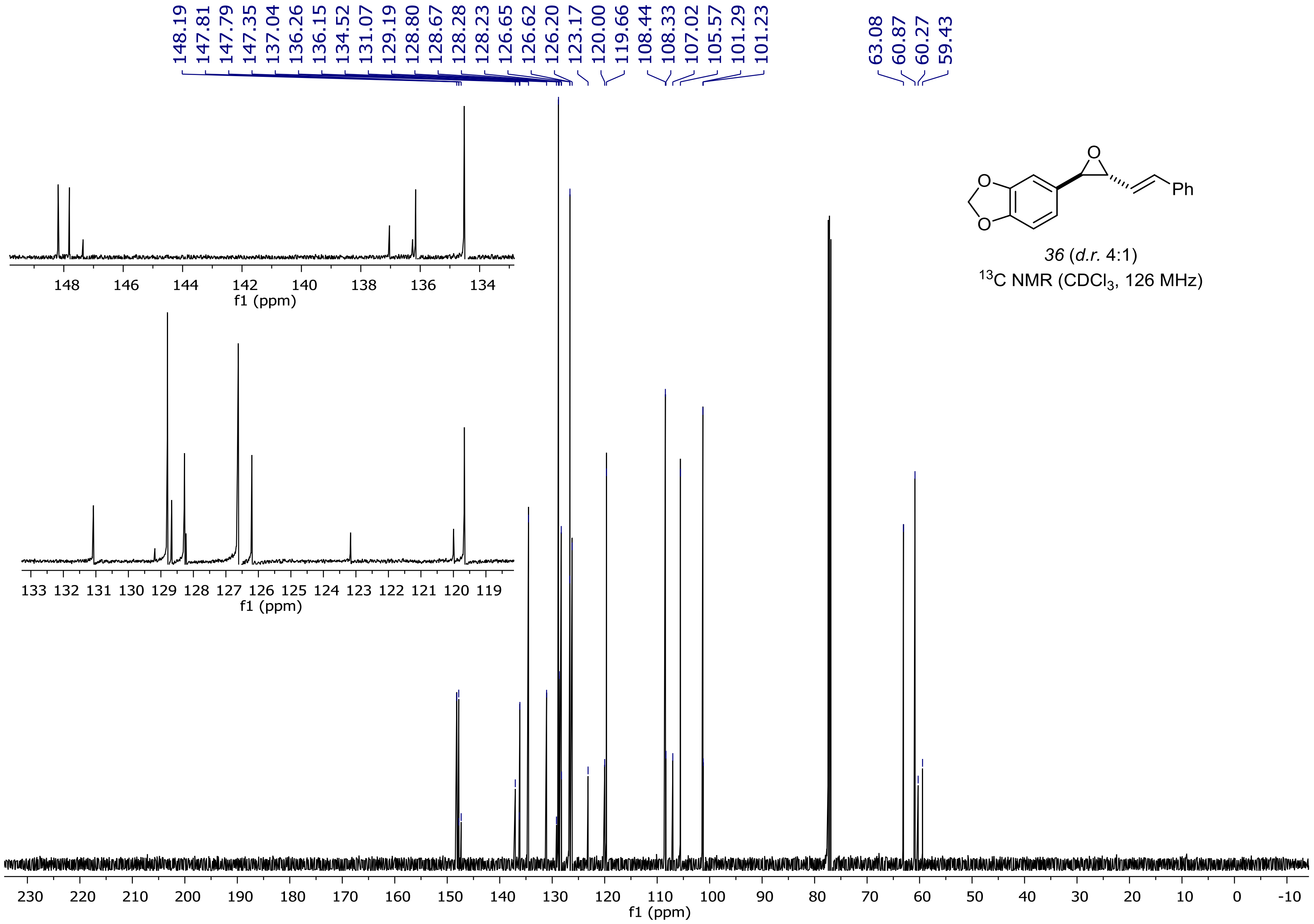


*36* (*d.r.* 4:1)

$^{13}$C NMR ($CDCl_3$, 126 MHz)

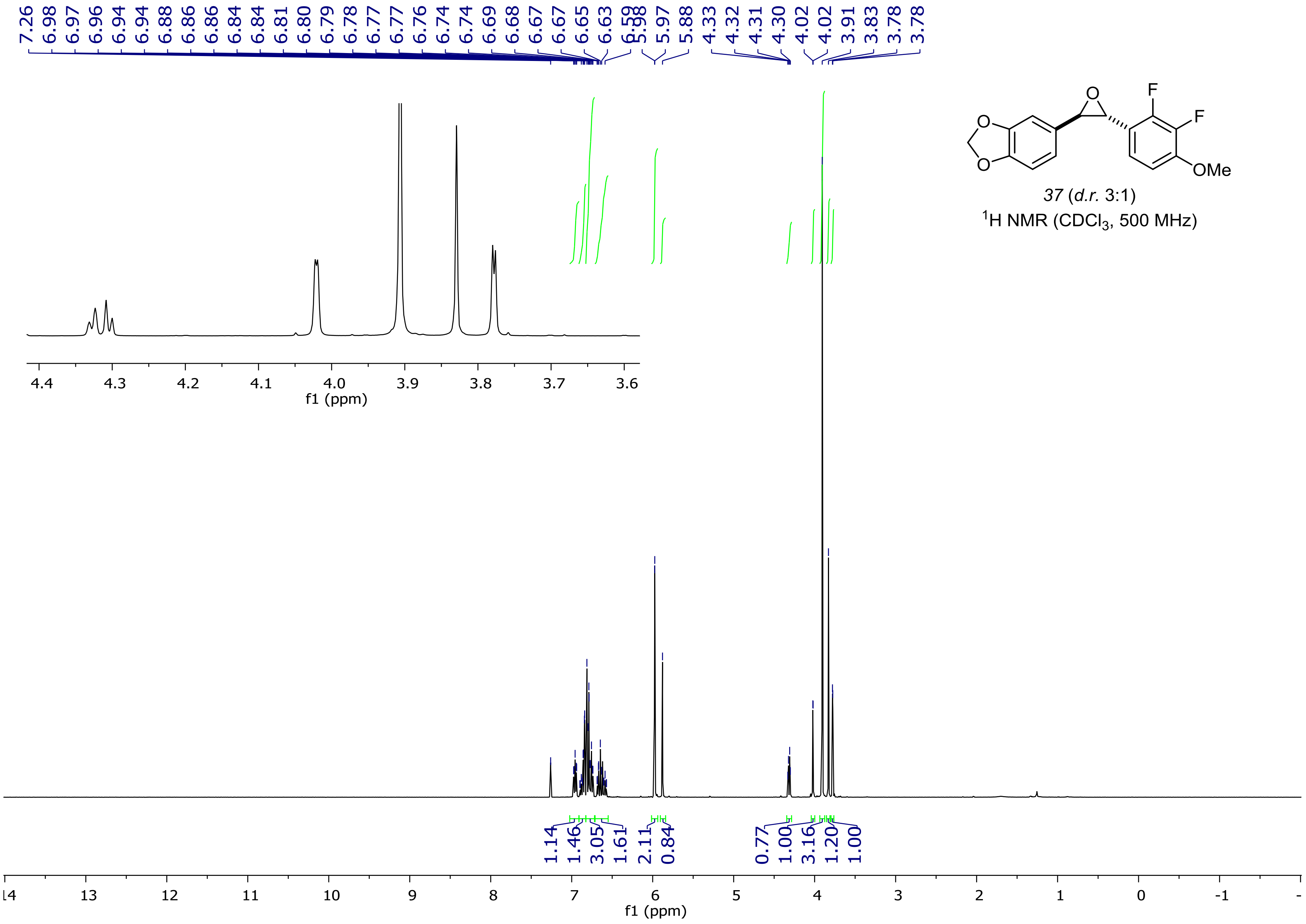

37 (d.r. 3:1)
1H NMR (CDCl3, 500 MHz)
OMe
f1 (ppm)

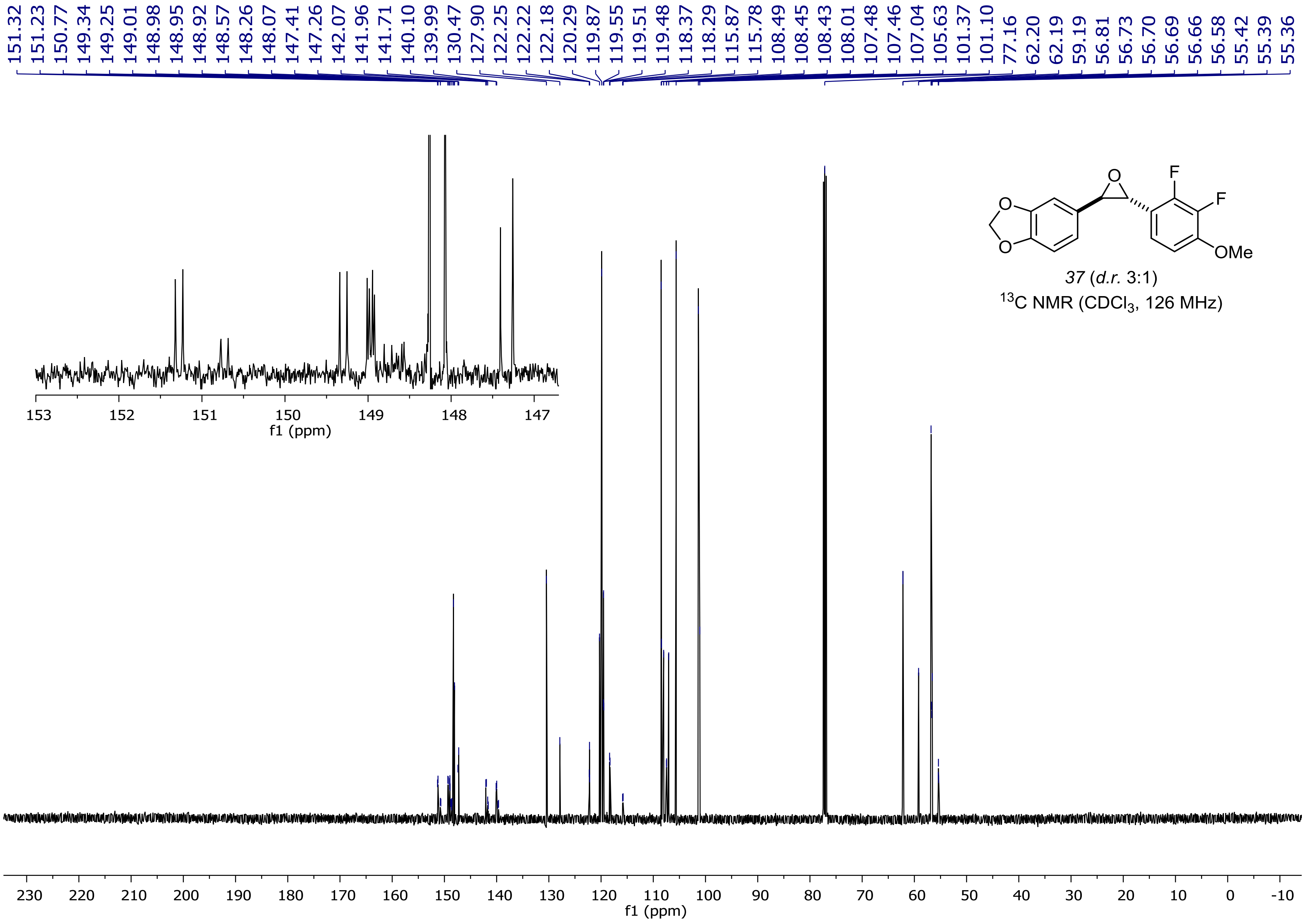

151.32
151.23
150.77
149.34
149.25
149.01
148.98
148.95
148.92
148.57
148.26
148.07
147.41
147.26
142.07
141.96
141.71
140.10
139.99
130.47
127.90
122.25
122.22
122.18
120.29
119.87
119.55
119.51
119.48
118.37
118.29
115.87
115.78
108.49
108.45
108.43
108.01
107.48
107.46
107.04
105.63
101.37
101.10
77.16
62.20
62.19
59.19
56.81
56.73
56.70
56.69
56.66
56.58
55.42
55.39
55.36
O
O
O
F
F
OMe
37 (d.r. 3:1)
$^{13}$C NMR (CDCl$_3$, 126 MHz)
153
152
151
150
149
148
147
f1 (ppm)
230
220
210
200
190
180
170
160
150
140
130
120
110
100
90
80
70
60
50
40
30
20
10
0
-10
f1 (ppm)

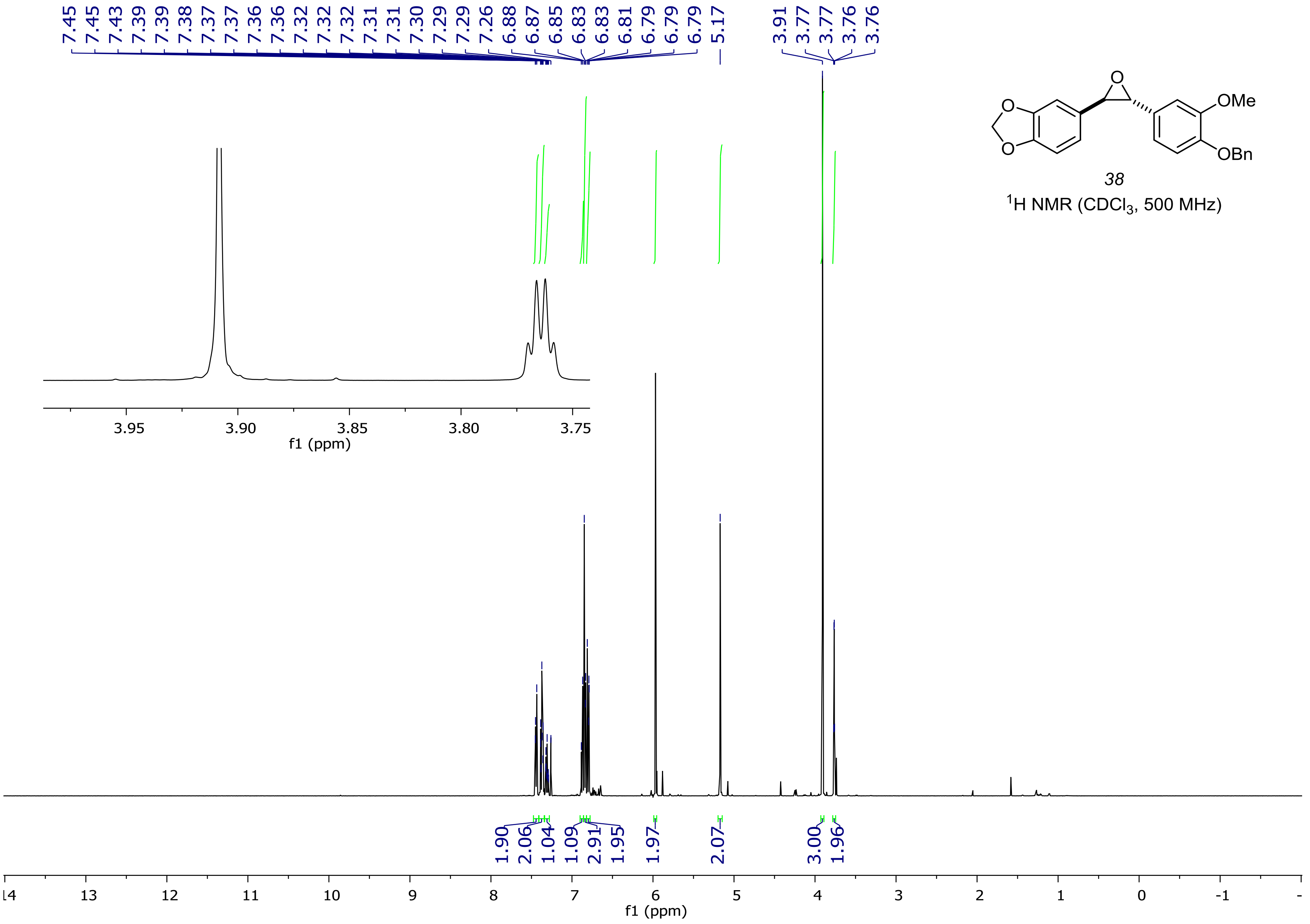
7.45 7.45 7.43 7.39 7.39 7.38 7.37 7.37 7.36 7.36 7.32 7.32 7.32 7.31 7.31 7.30 7.29 7.29 7.26 6.88 6.87 6.85 6.83 6.83 6.81 6.79 6.79 6.79 6.79 5.17
3.91 3.77 3.77 3.76 3.76
OMe
OBn
O
38
$^1$H NMR (CDCl$_3$, 500 MHz)
3.95 3.90 3.85 3.80 3.75
f1 (ppm)
1.90 2.06 1.04 1.09 2.91 1.95 1.97 2.07 3.00 1.96
14 13 12 11 10 9 8 7 6 5 4 3 2 1 0 -1
f1 (ppm)

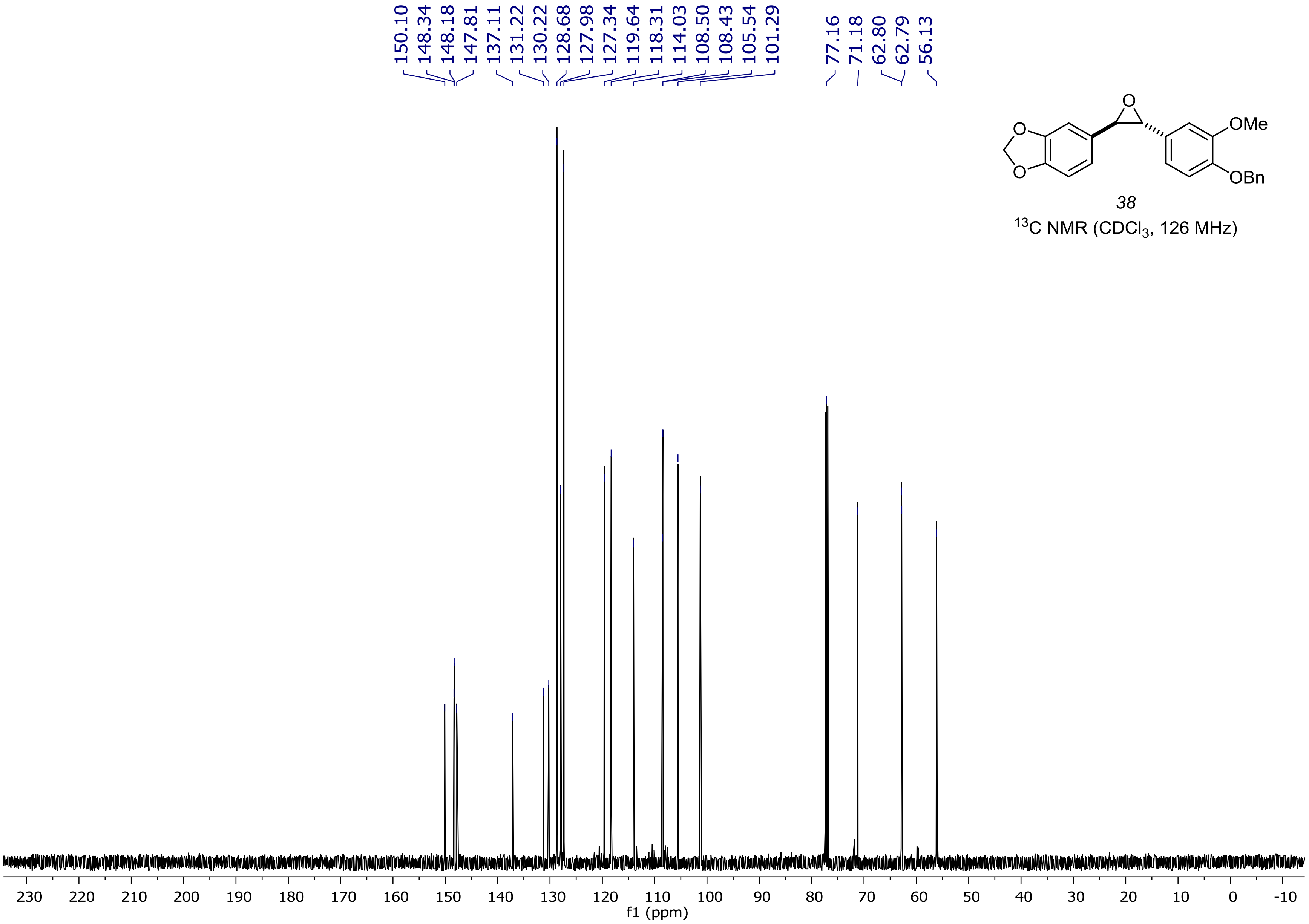


*38*

$^{13}$C NMR ($CDCl_3$, 126 MHz)

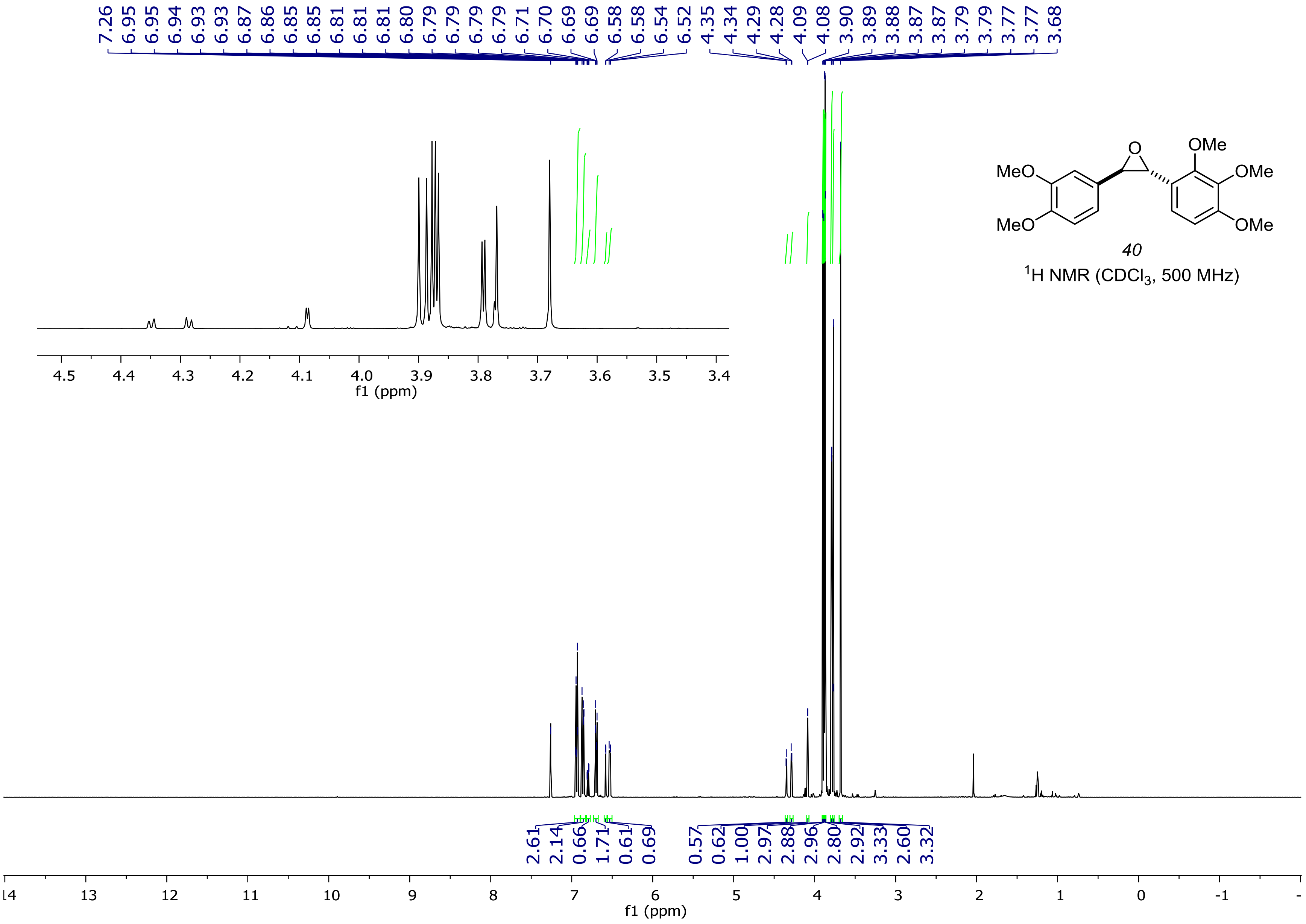
7.26 6.95 6.95 6.94 6.93 6.93 6.87 6.86 6.85 6.85 6.81 6.81 6.81 6.80 6.79 6.79 6.79 6.79 6.71 6.70 6.69 6.69 6.58 6.58 6.54 6.52 4.35 4.34 4.29 4.28 4.09 4.08 3.90 3.89 3.88 3.87 3.87 3.79 3.79 3.77 3.77 3.68
MeO
MeO
O
OMe
OMe
OMe
40
¹H NMR (CDCl3, 500 MHz)
f1 (ppm)
2.61 2.14 0.66 1.71 0.61 0.69 0.57 0.62 1.00 2.97 2.88 2.96 2.80 2.92 3.33 2.60 3.32
f1 (ppm)

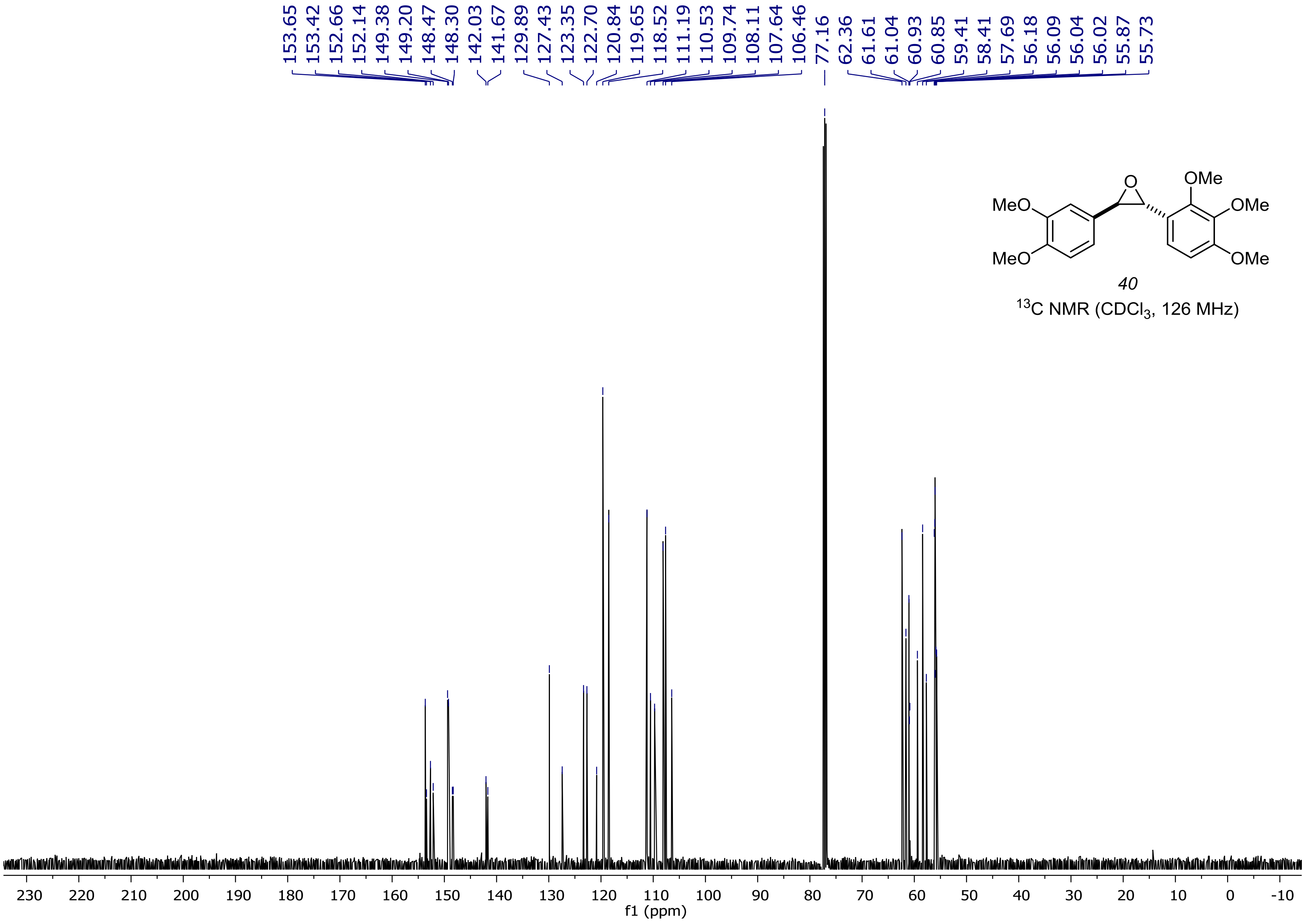
153.65
153.42
152.66
152.14
149.38
149.20
148.47
148.30
142.03
141.67
129.89
127.43
123.35
122.70
120.84
119.65
118.52
111.19
110.53
109.74
108.11
107.64
106.46
77.16
62.36
61.61
61.04
60.93
60.85
59.41
58.41
57.69
56.18
56.09
56.04
56.02
55.87
55.73
MeO
MeO
O
OMe
OMe
OMe
40
$^{13}$C NMR ($CDCl_3$, 126 MHz)
230 220 210 200 190 180 170 160 150 140 130 120 110 100 90 80 70 60 50 40 30 20 10 0 -10
f1 (ppm)

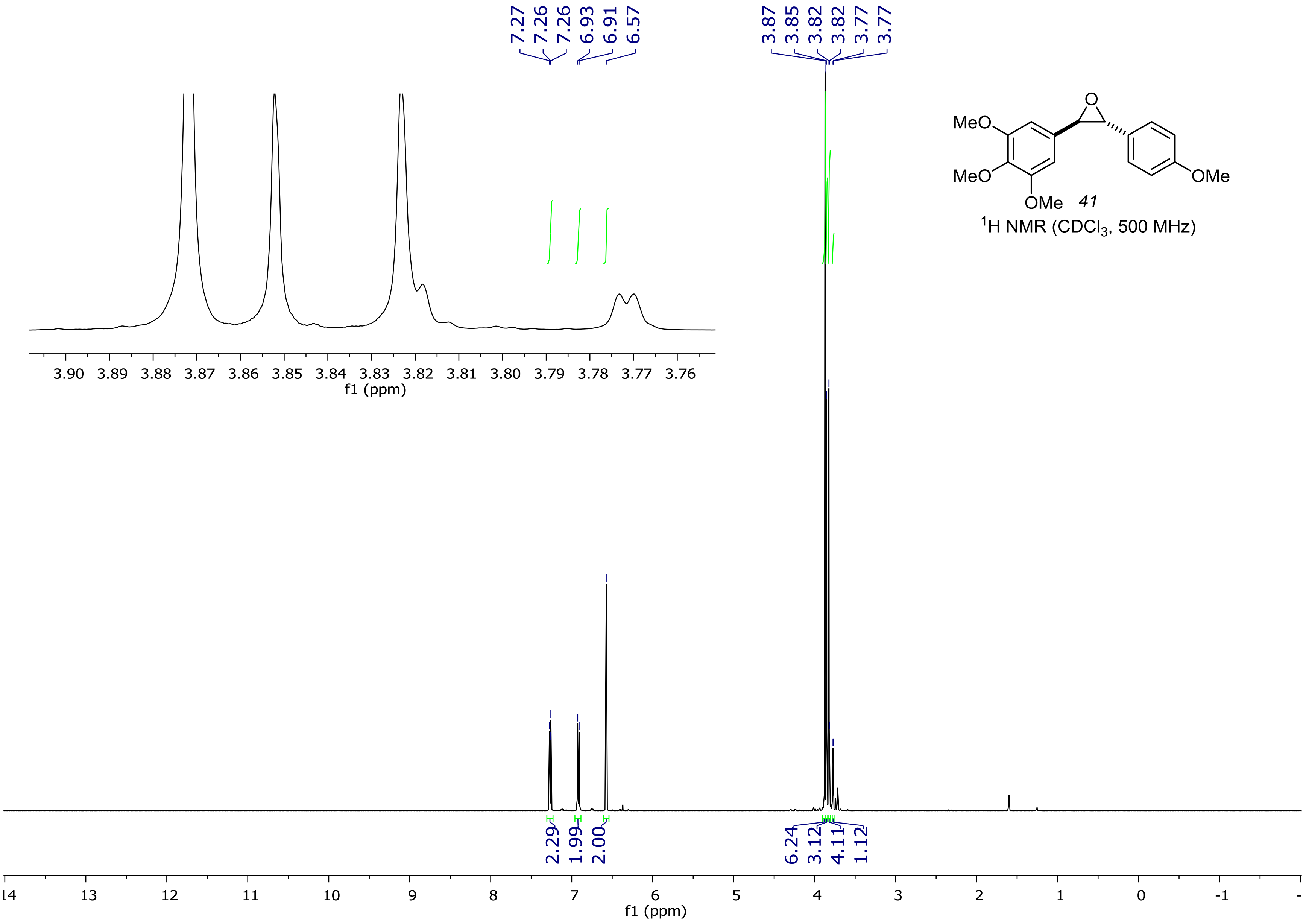
7.27
7.26
7.26
6.93
6.91
6.57
3.87
3.85
3.82
3.82
3.77
3.77
MeO
MeO
OMe
OMe
O
41
$^1$H NMR ($CDCl_3$, 500 MHz)
3.90 3.89 3.88 3.87 3.86 3.85 3.84 3.83 3.82 3.81 3.80 3.79 3.78 3.77 3.76
f1 (ppm)
2.29
1.99
2.00
6.24
3.12
4.11
1.12
13 12 11 10 9 8 7 6 5 4 3 2 1 0 -1
f1 (ppm)

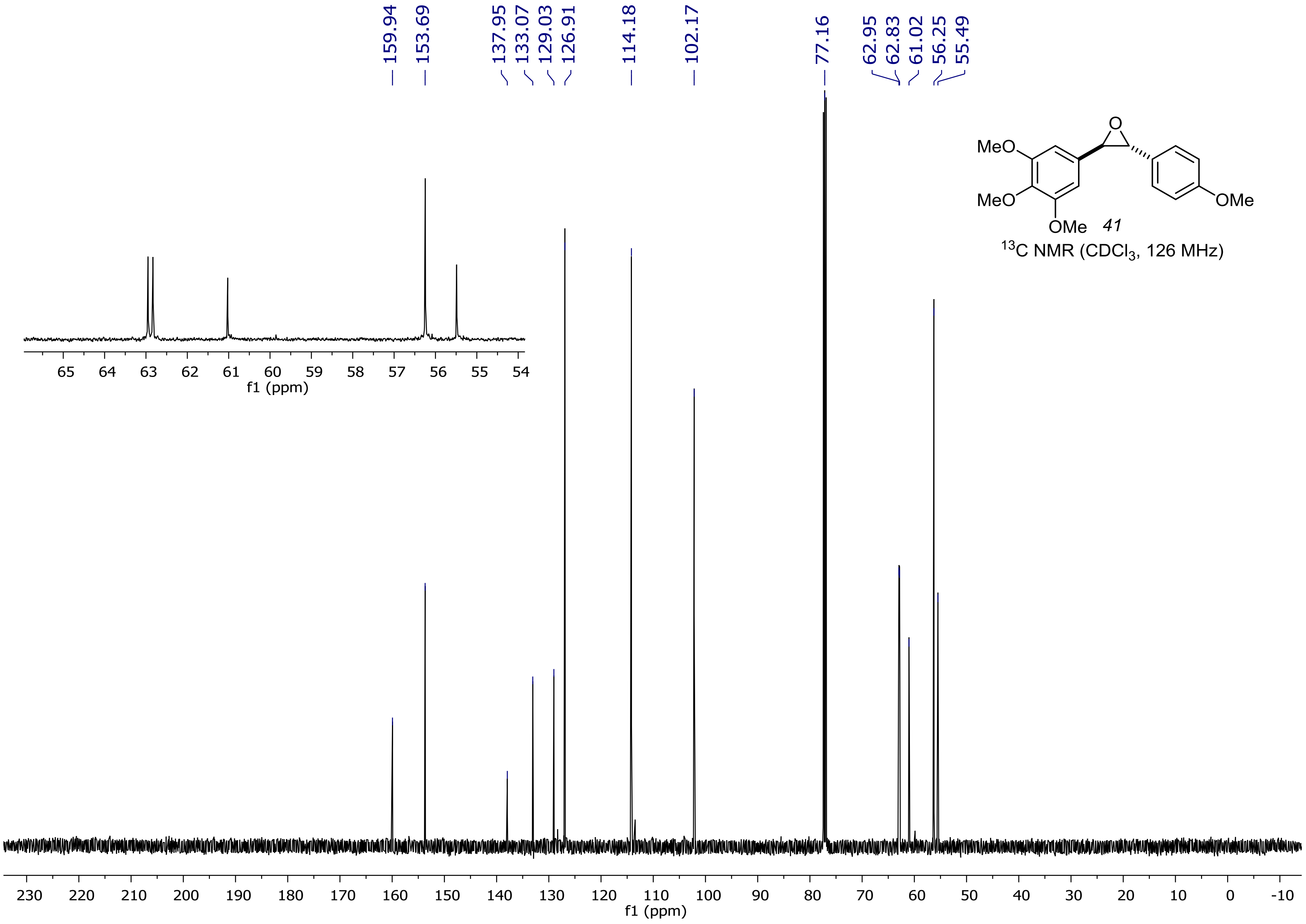

159.94
153.69
137.95
133.07
129.03
126.91
114.18
102.17
77.16
62.95
62.83
61.02
56.25
55.49
MeO
MeO
OMe
OMe
O
41
$^{13}$C NMR (CDCl$_3$, 126 MHz)
65 64 63 62 61 60 59 58 57 56 55 54
f1 (ppm)
230 220 210 200 190 180 170 160 150 140 130 120 110 100 90 80 70 60 50 40 30 20 10 0 -10
f1 (ppm)

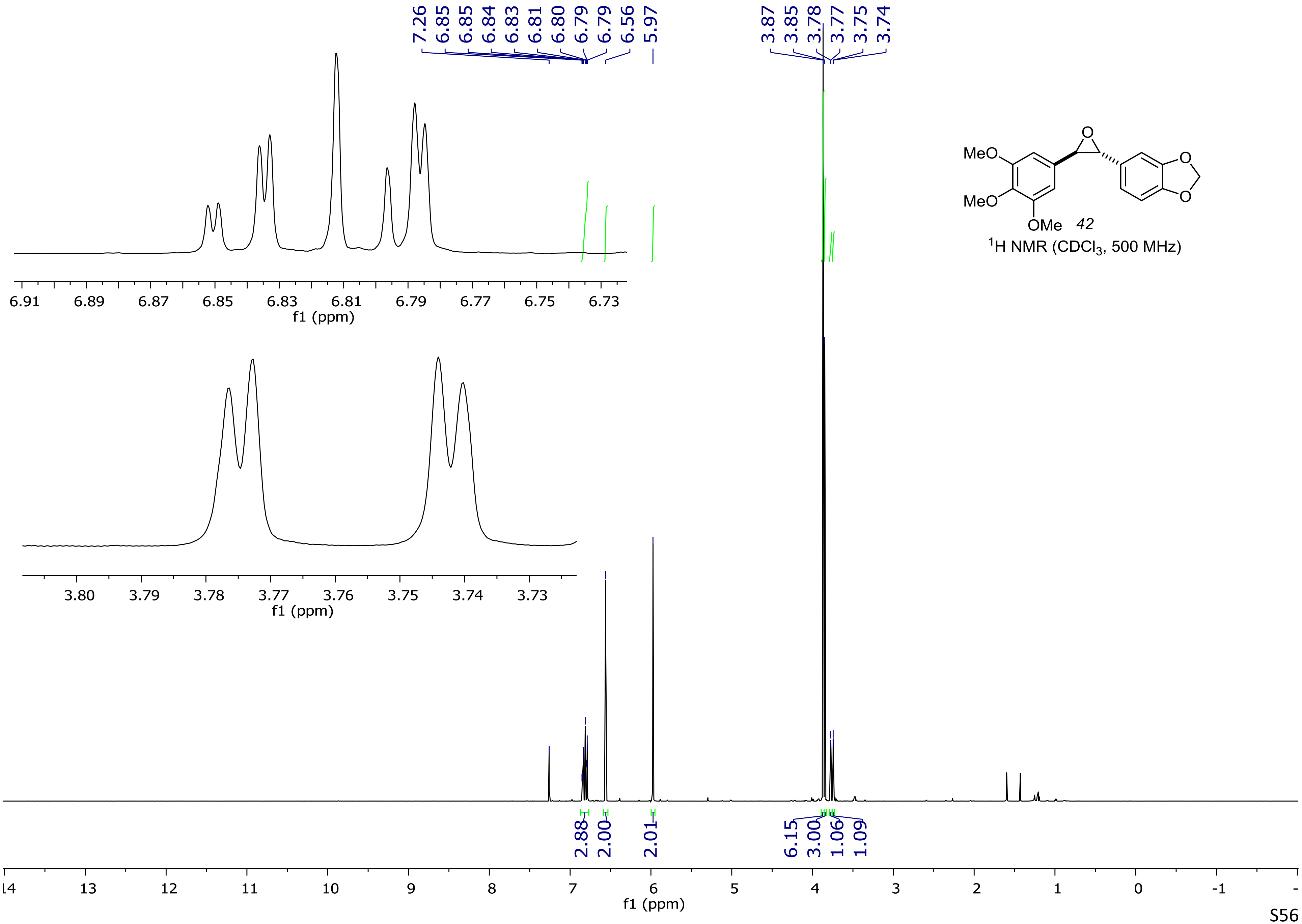


$^{1}$H NMR (CDCl$_3$, 500 MHz)

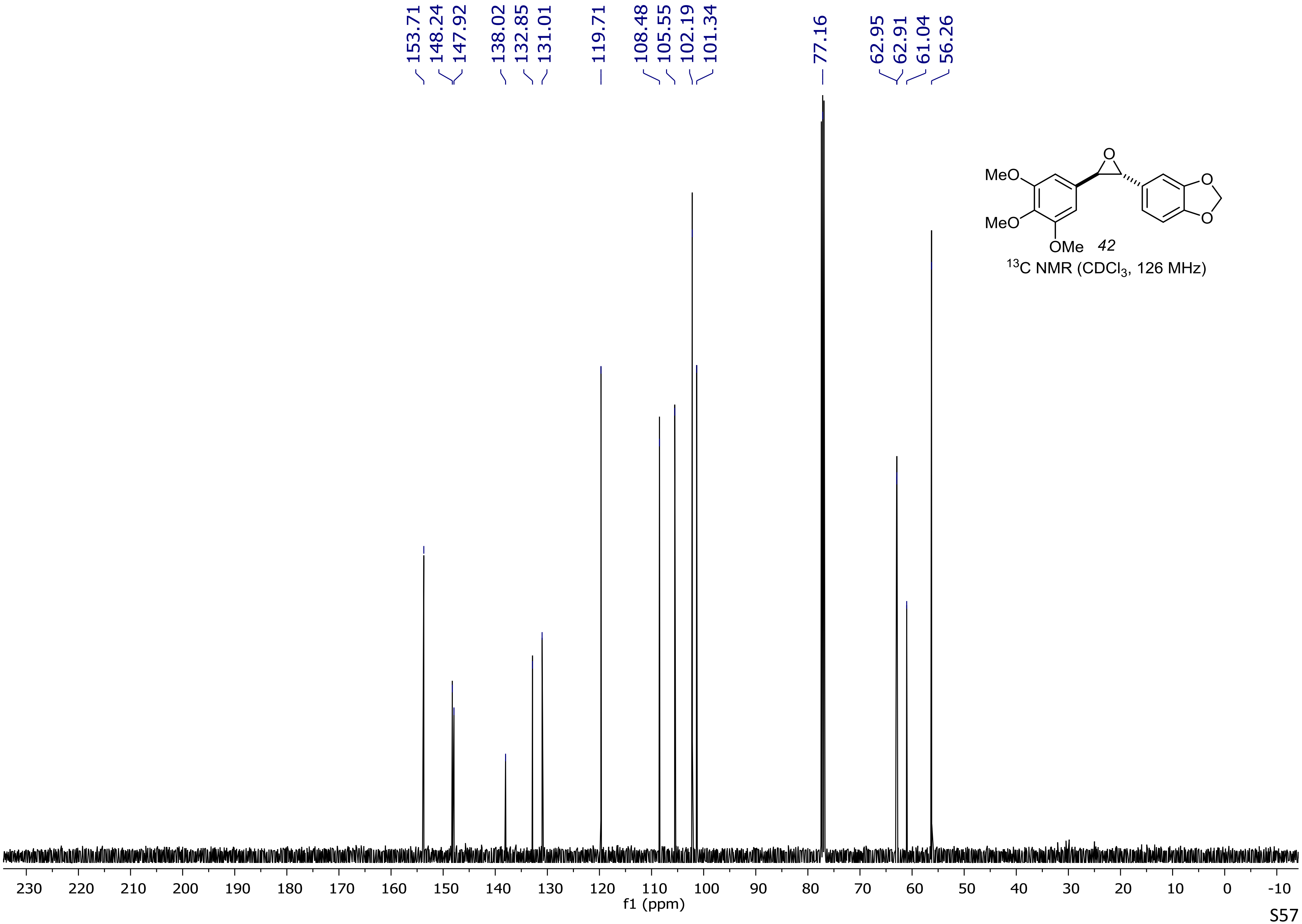
153.71
148.24
147.92
138.02
132.85
131.01
119.71
108.48
105.55
102.19
101.34
77.16
62.95
62.91
61.04
56.26
MeO
MeO
OMe
O
O
O
42
13C NMR (CDCl3, 126 MHz)
230 220 210 200 190 180 170 160 150 140 130 120 110 100 90 80 70 60 50 40 30 20 10 0 -10
f1 (ppm)

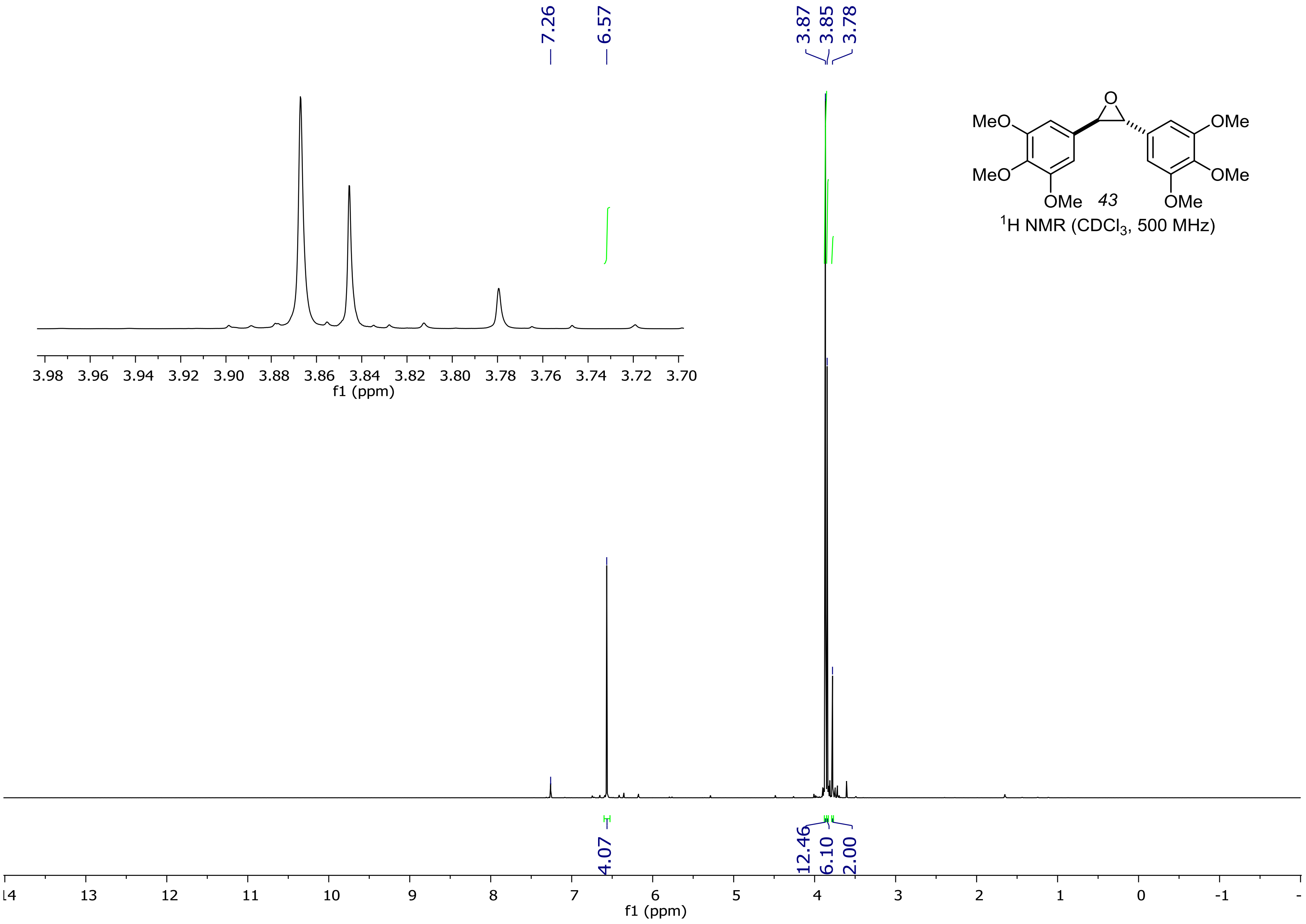
7.26
6.57
3.87
3.85
3.78
MeO
MeO
OMe
OMe
OMe
OMe
O
43
¹H NMR (CDCl₃, 500 MHz)
3.98 3.96 3.94 3.92 3.90 3.88 3.86 3.84 3.82 3.80 3.78 3.76 3.74 3.72 3.70
f1 (ppm)
4.07
12.46
6.10
2.00
13 12 11 10 9 8 7 6 5 4 3 2 1 0 -1
f1 (ppm)

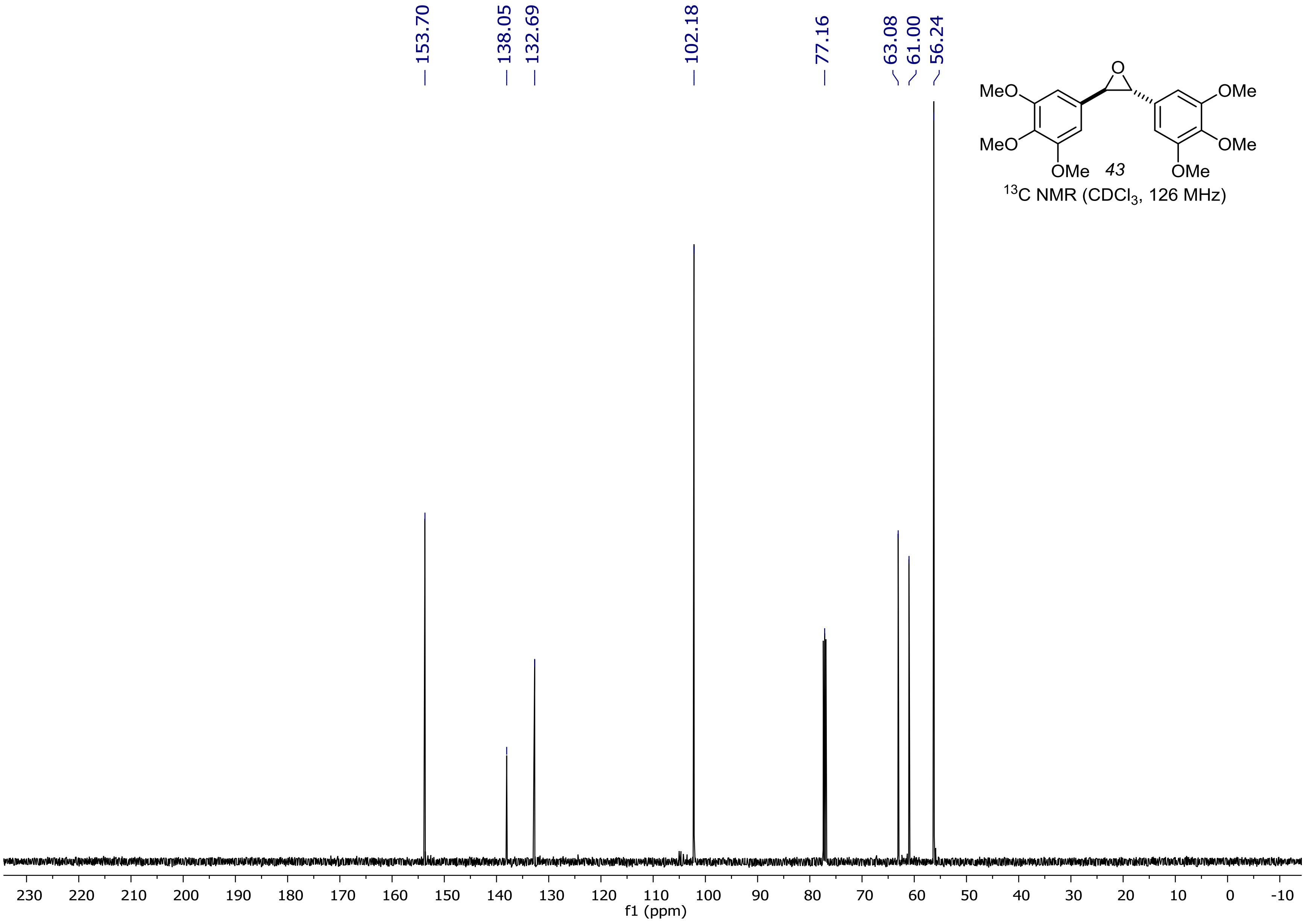
153.70
138.05
132.69
102.18
77.16
63.08
61.00
56.24
MeO
MeO
OMe
OMe
OMe
OMe
O
43
$^{13}$C NMR (CDCl$_3$, 126 MHz)
230 220 210 200 190 180 170 160 150 140 130 120 110 100 90 80 70 60 50 40 30 20 10 0 -10
f1 (ppm)

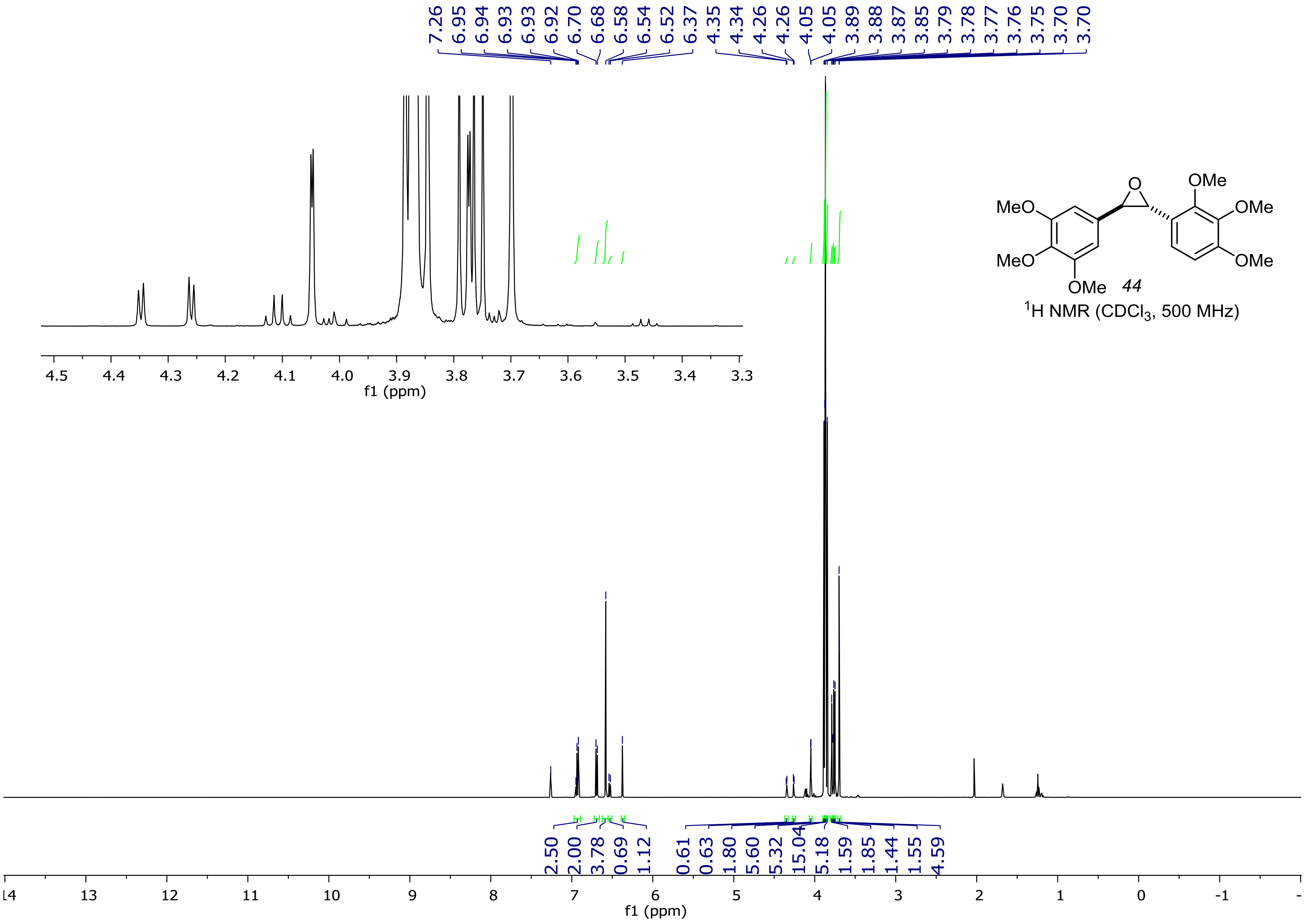
7.26 6.95 6.94 6.93 6.93 6.92 6.70 6.68 6.58 6.54 6.52 6.37 4.35 4.34 4.26 4.26 4.05 4.05 3.89 3.88 3.87 3.85 3.79 3.78 3.77 3.76 3.75 3.70 3.70
4.5 4.4 4.3 4.2 4.1 4.0 3.9 3.8 3.7 3.6 3.5 3.4 3.3
f1 (ppm)
MeO
MeO
OMe
O
OMe
OMe
OMe
44
¹H NMR (CDCl₃, 500 MHz)
2.50 2.00 3.78 0.69 1.12 0.61 0.63 1.80 5.60 5.32 15.04 5.18 1.59 1.85 1.44 1.55 4.59
14 13 12 11 10 9 8 7 6 5 4 3 2 1 0 -1
f1 (ppm)

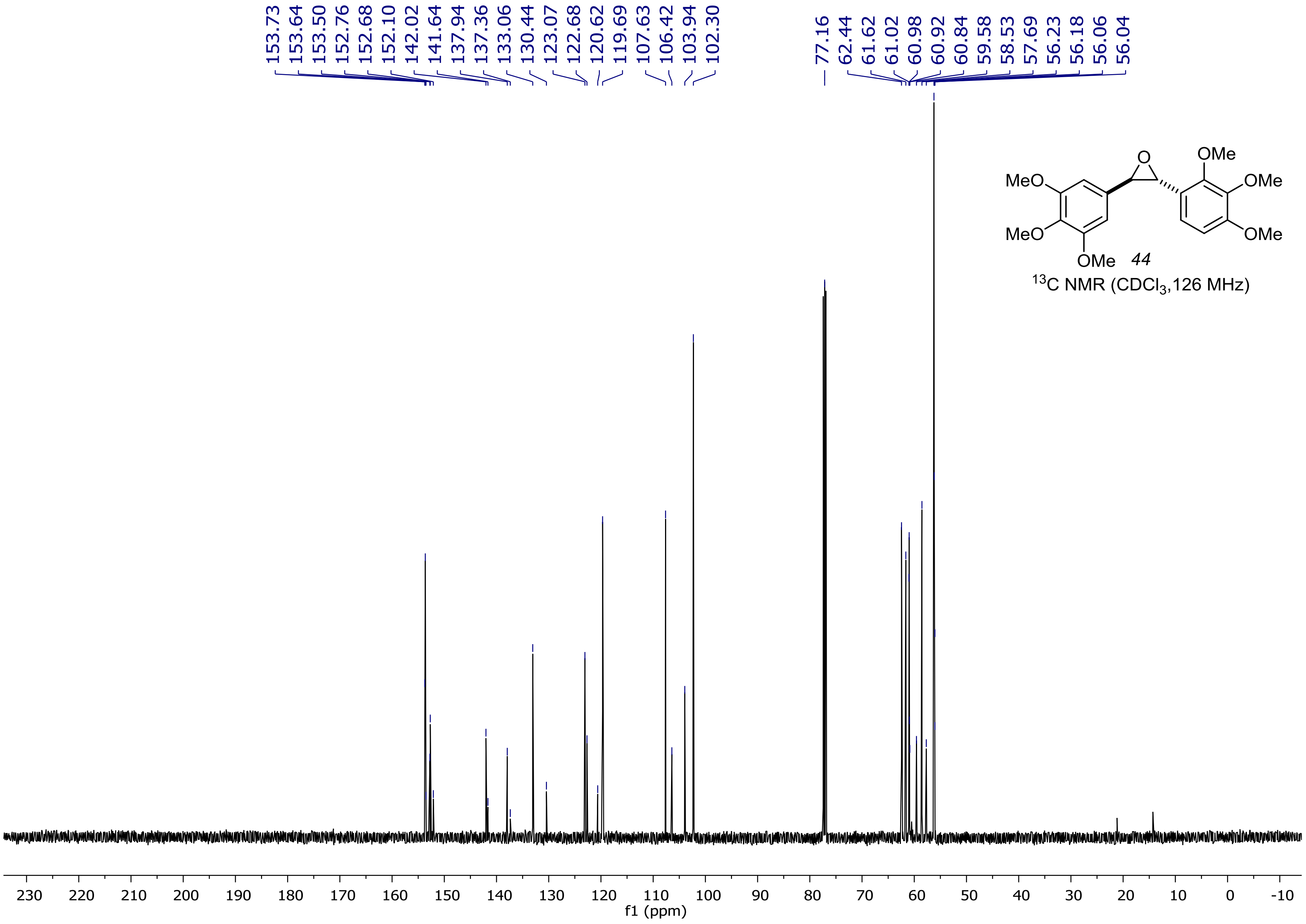
153.73
153.64
153.50
152.76
152.68
152.10
142.02
141.64
137.94
137.36
133.06
130.44
123.07
122.68
120.62
119.69
107.63
106.42
103.94
102.30
77.16
62.44
61.62
61.02
60.98
60.92
60.84
59.58
58.53
57.69
56.23
56.18
56.06
56.04
MeO
MeO
OMe
O
OMe
OMe
OMe
44
$^{13}$C NMR (CDCl$_3$,126 MHz)
230 220 210 200 190 180 170 160 150 140 130 120 110 100 90 80 70 60 50 40 30 20 10 0 -10
f1 (ppm)
